# Finding regulatory elements and regulatory motifs: a general probabilistic framework

**DOI:** 10.1186/1471-2105-8-S6-S4

**Published:** 2007-09-27

**Authors:** Erik van Nimwegen

**Affiliations:** 1 Biozentrum, University of Basel, and Swiss Institute of Bioinformatics, Klingelbergstrasse 50/70, Basel, Switzerland

## Abstract

Over the last two decades a large number of algorithms has been developed for regulatory motif finding. Here we show how many of these algorithms, especially those that model binding specificities of regulatory factors with position specific weight matrices (WMs), naturally arise within a general Bayesian probabilistic framework. We discuss how WMs are constructed from sets of regulatory sites, how sites for a given WM can be discovered by scanning of large sequences, how to cluster WMs, and more generally how to cluster large sets of sites from different WMs into clusters. We discuss how 'regulatory modules', clusters of sites for subsets of WMs, can be found in large intergenic sequences, and we discuss different methods for *ab initio *motif finding, including expectation maximization (EM) algorithms, and motif sampling algorithms. Finally, we extensively discuss how module finding methods and *ab initio *motif finding methods can be extended to take phylogenetic relations between the input sequences into account, i.e. we show how motif finding and phylogenetic footprinting can be integrated in a rigorous probabilistic framework. The article is intended for readers with a solid background in applied mathematics, and preferably with some knowledge of general Bayesian probabilistic methods. The main purpose of the article is to elucidate that all these methods are not a disconnected set of individual algorithmic recipes, but that they are just different facets of a single integrated probabilistic theory.

## The weight matrix representation of regulatory sites

The first step in any algorithm for identifying regulatory sites in DNA or RNA is to decide on a mathematical representation of the binding sites. For definiteness, let us assume we are considering a DNA binding factor which, when bound to DNA, covers a DNA segment of *l *base pairs long. For any length-*l *sequence *s *there will be a well-defined (but generally unknown) binding free-energy *E*(*s*) to the regulatory factor. A key assumption [1] that is introduced at this point is that the energy *E*(*s*) can be written as the sum of independent contributions *E*_*i*_(*s*_*i*_) from each of the bases *s*_*i *_in segment *s*, i.e.

E(s)=∑i=1lEi(si).
 MathType@MTEF@5@5@+=feaafiart1ev1aaatCvAUfKttLearuWrP9MDH5MBPbIqV92AaeXatLxBI9gBaebbnrfifHhDYfgasaacH8akY=wiFfYdH8Gipec8Eeeu0xXdbba9frFj0=OqFfea0dXdd9vqai=hGuQ8kuc9pgc9s8qqaq=dirpe0xb9q8qiLsFr0=vr0=vr0dc8meaabaqaciaacaGaaeqabaqabeGadaaakeaacqWGfbqrcqGGOaakcqWGZbWCcqGGPaqkcqGH9aqpdaaeWbqaaiabdweafnaaBaaaleaacqWGPbqAaeqaaOGaeiikaGIaem4Cam3aaSbaaSqaaiabdMgaPbqabaGccqGGPaqkcqGGUaGlaSqaaiabdMgaPjabg2da9iabigdaXaqaaiabdYgaSbqdcqGHris5aaaa@4114@

This assumption of course generally only holds to some extent. Large-scale *in vitro *studies have shown that the binding energies can deviate from this simple additivity assumption [2]. However, these deviations are typically small, and moreover they seem generally restricted to segments with low binding-energy [3]. At this point it is not yet clear to what extent and for what fraction of regulatory factors, the additivity assumption holds. Some researchers believe that, at least for some factors, functional binding sites deviate significantly from this assumption, and this may well be the case. However, it is this author's experience that in collections of experimentally determined binding sites there is little evidence of correlations between the nucleotides occurring at different positions which, as we will see below, supports the additivity assumption for functional binding sites.

The crucial assumption which underlies the whole idea of 'finding regulatory sites' is that the set of all 4^*l *^possible segments *s *can be meaningfully divided into 'binding sites' and all other sequences. Since this is not *a priori *clear at all, it is good to consider what this assumption entails. At a given concentration *c *of the regulatory factor, the probability that a sequence segment *s *will be bound by the factor is given by an expression of the following form [4,5]

Pbound(s)=ceβE(s)ceβE(s)+K,
 MathType@MTEF@5@5@+=feaafiart1ev1aaatCvAUfKttLearuWrP9MDH5MBPbIqV92AaeXatLxBI9gBaebbnrfifHhDYfgasaacH8akY=wiFfYdH8Gipec8Eeeu0xXdbba9frFj0=OqFfea0dXdd9vqai=hGuQ8kuc9pgc9s8qqaq=dirpe0xb9q8qiLsFr0=vr0=vr0dc8meaabaqaciaacaGaaeqabaqabeGadaaakeaacqWGqbaudaWgaaWcbaGaeeOyaiMaee4Ba8MaeeyDauNaeeOBa4MaeeizaqgabeaakiabcIcaOiabdohaZjabcMcaPiabg2da9maalaaabaGaem4yamMaemyzau2aaWbaaSqabeaaiiGacqWFYoGycqWGfbqrcqGGOaakcqWGZbWCcqGGPaqkaaaakeaacqWGJbWycqWGLbqzdaahaaWcbeqaaiab=j7aIjabdweafjabcIcaOiabdohaZjabcMcaPaaakiabgUcaRiabdUealbaacqGGSaalaaa@4D54@

where *β *= 1/(*kT*) is the inverse temperature, and *K *is a constant. (We here ignore the fact that the factor may bind at segments that overlap *s*, which would prevent the factor from binding at *s*. Below we will derive the general solution that takes this complication into account.) The expression (2) is an s-shaped function that goes from 0 to 1 as *ce*^*βE*(*s*) ^goes from much smaller than *K *to much larger than *K*. Therefore, at a given concentration *c *one can naturally separate sequences *s *into binders, i.e. those with *E*(*s*) > log(*K/c*)/*β *and non-binders with *E*(*s*) < log(*K/c*)/*β*. If the concentration of the (active) regulatory factor were to vary continuously between different cellular states, then the set of sites bound by the factor would also vary continuously and it would not make much sense to divide segments *s *into binders and non-binders. However, if in physiological conditions the regulatory factor primarily switches between an 'off' state, i.e. low concentration *c*_off_, and an 'on' state, i.e. high concentration *c*_on _than there would be a well defined set of sites that are bound when the factor is 'on' and unbound when the factor is 'off', i.e. those with energies in the range

1βlog⁡(Kcon)<E(s)<1βlog⁡(Kcoff).
 MathType@MTEF@5@5@+=feaafiart1ev1aaatCvAUfKttLearuWrP9MDH5MBPbIqV92AaeXatLxBI9gBaebbnrfifHhDYfgasaacH8akY=wiFfYdH8Gipec8Eeeu0xXdbba9frFj0=OqFfea0dXdd9vqai=hGuQ8kuc9pgc9s8qqaq=dirpe0xb9q8qiLsFr0=vr0=vr0dc8meaabaqaciaacaGaaeqabaqabeGadaaakeaadaWcaaqaaiabigdaXaqaaGGaciab=j7aIbaacyGGSbaBcqGGVbWBcqGGNbWzdaqadaqaamaalaaabaGaem4saSeabaGaem4yam2aaSbaaSqaaiabb+gaVjabb6gaUbqabaaaaaGccaGLOaGaayzkaaGaeyipaWJaemyrauKaeiikaGIaem4CamNaeiykaKIaeyipaWZaaSaaaeaacqaIXaqmaeaacqWFYoGyaaGagiiBaWMaei4Ba8Maei4zaC2aaeWaaeaadaWcaaqaaiabdUealbqaaiabdogaJnaaBaaaleaacqqGVbWBcqqGMbGzcqqGMbGzaeqaaaaaaOGaayjkaiaawMcaaiabc6caUaaa@5099@

Therefore, this set of binding sites may be characterized by a typical energy that lies somewhere in the middle of this range.The assumption that is thus generally made [1] is that binding sites are characterized by an average binding energy E¯
 MathType@MTEF@5@5@+=feaafiart1ev1aaatCvAUfKttLearuWrP9MDH5MBPbIqV92AaeXatLxBI9gBaebbnrfifHhDYfgasaacH8akY=wiFfYdH8Gipec8Eeeu0xXdbba9frFj0=OqFfea0dXdd9vqai=hGuQ8kuc9pgc9s8qqaq=dirpe0xb9q8qiLsFr0=vr0=vr0dc8meaabaqaciaacaGaaeqabaqabeGadaaakeaacuWGfbqrgaqeaaaa@2DD7@. We now want to derive the probability *P*(*s*) that a randomly chosen binding site will have sequence *s*, given only the constraint that the average energy of the sites is E¯
 MathType@MTEF@5@5@+=feaafiart1ev1aaatCvAUfKttLearuWrP9MDH5MBPbIqV92AaeXatLxBI9gBaebbnrfifHhDYfgasaacH8akY=wiFfYdH8Gipec8Eeeu0xXdbba9frFj0=OqFfea0dXdd9vqai=hGuQ8kuc9pgc9s8qqaq=dirpe0xb9q8qiLsFr0=vr0=vr0dc8meaabaqaciaacaGaaeqabaqabeGadaaakeaacuWGfbqrgaqeaaaa@2DD7@. The maximum entropy formalism [6], i.e. as applied in statistical mechanics, prescribes that distribution *P*(*s*) is given by

P(s)=eλE(s)∑s′eλE(s′)=∏i=1l[eλEi(si)∑αeλEi(α)],
 MathType@MTEF@5@5@+=feaafiart1ev1aaatCvAUfKttLearuWrP9MDH5MBPbIqV92AaeXatLxBI9gBaebbnrfifHhDYfgasaacH8akY=wiFfYdH8Gipec8Eeeu0xXdbba9frFj0=OqFfea0dXdd9vqai=hGuQ8kuc9pgc9s8qqaq=dirpe0xb9q8qiLsFr0=vr0=vr0dc8meaabaqaciaacaGaaeqabaqabeGadaaakeaacqWGqbaucqGGOaakcqWGZbWCcqGGPaqkcqGH9aqpdaWcaaqaaiabdwgaLnaaCaaaleqabaacciGae83UdWMaemyrauKaeiikaGIaem4CamNaeiykaKcaaaGcbaWaaabeaeaacqWGLbqzdaahaaWcbeqaaiab=T7aSjabdweafjabcIcaOiqbdohaZzaafaGaeiykaKcaaaqaaiqbdohaZzaafaaabeqdcqGHris5aaaakiabg2da9maarahabaWaamWaaeaadaWcaaqaaiabdwgaLnaaCaaaleqabaGae83UdWMaemyrau0aaSbaaWqaaiabdMgaPbqabaWccqGGOaakcqWGZbWCdaWgaaadbaGaemyAaKgabeaaliabcMcaPaaaaOqaamaaqababaGaemyzau2aaWbaaSqabeaacqWF7oaBcqWGfbqrdaWgaaadbaGaemyAaKgabeaaliabcIcaOiab=f7aHjabcMcaPaaaaeaacqWFXoqyaeqaniabggHiLdaaaaGccaGLBbGaayzxaaaaleaacqWGPbqAcqGH9aqpcqaIXaqmaeaacqWGSbaBa0Gaey4dIunakiabcYcaSaaa@6656@

where the sum over *s' *is over all length-*l *sequences, and the sum over *α *is over the four bases. The Langrangian multiplier *λ *is chosen such that <*E*> = ∑_*s *_*E*(*s*) *P*(*s*) = E¯
 MathType@MTEF@5@5@+=feaafiart1ev1aaatCvAUfKttLearuWrP9MDH5MBPbIqV92AaeXatLxBI9gBaebbnrfifHhDYfgasaacH8akY=wiFfYdH8Gipec8Eeeu0xXdbba9frFj0=OqFfea0dXdd9vqai=hGuQ8kuc9pgc9s8qqaq=dirpe0xb9q8qiLsFr0=vr0=vr0dc8meaabaqaciaacaGaaeqabaqabeGadaaakeaacuWGfbqrgaqeaaaa@2DD7@. Note that this is the same functional form as the well-known Boltzmann distribution. To avoid confusion, note also that equations (2) and (4) are probability distributions over entirely different spaces. The former takes a fixed sequence segment *s *and compares the probabilities of the bound and unbound states for this sequence segment, whereas the latter assigns the probabilities that a binding site will take on any of the 4^*l *^possible sequences. In equation (4) the bases at different positions are independent, i.e. P(s)=∏i=1lPi(si)
MathType@MTEF@5@5@+=feaafiart1ev1aaatCvAUfKttLearuWrP9MDH5MBPbIqV92AaeXatLxBI9gBaebbnrfifHhDYfgasaacH8akY=wiFfYdH8Gipec8Eeeu0xXdbba9frFj0=OqFfea0dXdd9vqai=hGuQ8kuc9pgc9s8qqaq=dirpe0xb9q8qiLsFr0=vr0=vr0dc8meaabaqaciaacaGaaeqabaqabeGadaaakeaacqWGqbaucqGGOaakcqWGZbWCcqGGPaqkcqGH9aqpdaqeWaqaaiabdcfaqnaaBaaaleaacqWGPbqAaeqaaOGaeiikaGIaem4Cam3aaSbaaSqaaiabdMgaPbqabaGccqGGPaqkaSqaaiabdMgaPjabg2da9iabigdaXaqaaiabdYgaSbqdcqGHpis1aaaa@400B@ with

Pi(si)=eλEi(si)∑αeλEi(α).
 MathType@MTEF@5@5@+=feaafiart1ev1aaatCvAUfKttLearuWrP9MDH5MBPbIqV92AaeXatLxBI9gBaebbnrfifHhDYfgasaacH8akY=wiFfYdH8Gipec8Eeeu0xXdbba9frFj0=OqFfea0dXdd9vqai=hGuQ8kuc9pgc9s8qqaq=dirpe0xb9q8qiLsFr0=vr0=vr0dc8meaabaqaciaacaGaaeqabaqabeGadaaakeaacqWGqbaudaWgaaWcbaGaemyAaKgabeaakiabcIcaOiabdohaZnaaBaaaleaacqWGPbqAaeqaaOGaeiykaKIaeyypa0ZaaSaaaeaacqWGLbqzdaahaaWcbeqaaGGaciab=T7aSjabdweafnaaBaaameaacqWGPbqAaeqaaSGaeiikaGIaem4Cam3aaSbaaWqaaiabdMgaPbqabaWccqGGPaqkaaaakeaadaaeqaqaaiabdwgaLnaaCaaaleqabaGae83UdWMaemyrau0aaSbaaWqaaiabdMgaPbqabaWccqGGOaakcqWFXoqycqGGPaqkaaaabaGae8xSdegabeqdcqGHris5aaaakiabc6caUaaa@4D4E@

This property allows us to define a *position specific weight matrix *(WM) *w *with components

wαi=wλEi(α)∑α′eλEi(α′).
 MathType@MTEF@5@5@+=feaafiart1ev1aaatCvAUfKttLearuWrP9MDH5MBPbIqV92AaeXatLxBI9gBaebbnrfifHhDYfgasaacH8akY=wiFfYdH8Gipec8Eeeu0xXdbba9frFj0=OqFfea0dXdd9vqai=hGuQ8kuc9pgc9s8qqaq=dirpe0xb9q8qiLsFr0=vr0=vr0dc8meaabaqaciaacaGaaeqabaqabeGadaaakeaacqWG3bWDdaqhaaWcbaacciGae8xSdegabaGaemyAaKgaaOGaeyypa0ZaaSaaaeaacqWG3bWDdaahaaWcbeqaaiab=T7aSjabdweafnaaBaaameaacqWGPbqAaeqaaSGaeiikaGIae8xSdeMaeiykaKcaaaGcbaWaaabeaeaacqWGLbqzdaahaaWcbeqaaiab=T7aSjabdweafnaaBaaameaacqWGPbqAaeqaaSGaeiikaGIaf8xSdeMbauaacqGGPaqkaaaabaGaf8xSdeMbauaaaeqaniabggHiLdaaaOGaeiOla4caaa@4959@

That is, we can represent regulatory sites by WMs, and find the following expression for the probability that a binding site has sequence *s*:

P(s|w)=∏i=1lwsii.
 MathType@MTEF@5@5@+=feaafiart1ev1aaatCvAUfKttLearuWrP9MDH5MBPbIqV92AaeXatLxBI9gBaebbnrfifHhDYfgasaacH8akY=wiFfYdH8Gipec8Eeeu0xXdbba9frFj0=OqFfea0dXdd9vqai=hGuQ8kuc9pgc9s8qqaq=dirpe0xb9q8qiLsFr0=vr0=vr0dc8meaabaqaciaacaGaaeqabaqabeGadaaakeaacqWGqbaucqGGOaakcqWGZbWCcqGG8baFcqWG3bWDcqGGPaqkcqGH9aqpdaqeWbqaaiabdEha3naaDaaaleaacqWGZbWCdaWgaaadbaGaemyAaKgabeaaaSqaaiabdMgaPbaaaeaacqWGPbqAcqGH9aqpcqaIXaqmaeaacqWGSbaBa0Gaey4dIunakiabc6caUaaa@42BA@

Finally, note that *P*(*s*|*w*) gives the probability that a given binding site will have sequence *s*, which should be carefully distinguished from the probability *P*(*w*|*s*) that a sequence segment *s *is a binding site for *w*. The latter cannot be calculated without specifying how likely *s *is to arise under alternative hypotheses as will be discussed in detail below.

Weight matrices are probably the most commonly used representation of regulatory sites and, as has just been shown, can be derived under the assumptions that the contribution to the binding energy from bases at different positions in the site are independent, and that functional binding sites are characterized by a given average binding energy. In this chapter we will focus on regulatory motif finding methods that use WMs. It should be noted, however, that in some circumstances regulatory sites can be adequately represented by either specific DNA words, i.e. when the regulatory factor recognizes essentially only a single sequence segment, or by regular expressions, and there is a substantial amount of work on motif finding in this context. There is also a moderate amount of work on more complex representations of regulatory sites, such as hidden Markov models that allow sites of varying length and correlations between bases at neighboring positions [2,7].

## Finding WM matches

Assume that we are in possession of a WM *w *that summarizes the binding specificity of a regulatory factor. One of the simplest applications is to 'scan' one or more sequences for 'matches' to this WM. Let *s *denote some sequence of length *L*, where *L *is typically much larger than the length *l *of the WM. We now want to infer if one or more sites for this WM occur in this sequence. Probabilistic inference always [6] takes the following general form

1. Enumerate all possible hypotheses *H *that could have accounted for the data *D*.

2. Assign prior probabilities *P*(*H*) to each of these hypotheses.

3. Define a likelihood model that gives the probability *P*(*D*|*H*) of producing the entire data *D *under each of the hypotheses *H*.

4. The posterior probability *P*(*H*|*D*)for each of the hypotheses is then given by Bayes' theorem:

P(H|D)=P(D|H)P(H)∑H˜P(D|H˜)P(H˜).
 MathType@MTEF@5@5@+=feaafiart1ev1aaatCvAUfKttLearuWrP9MDH5MBPbIqV92AaeXatLxBI9gBaebbnrfifHhDYfgasaacH8akY=wiFfYdH8Gipec8Eeeu0xXdbba9frFj0=OqFfea0dXdd9vqai=hGuQ8kuc9pgc9s8qqaq=dirpe0xb9q8qiLsFr0=vr0=vr0dc8meaabaqaciaacaGaaeqabaqabeGadaaakeaacqWGqbaucqGGOaakcqWGibascqGG8baFcqWGebarcqGGPaqkcqGH9aqpdaWcaaqaaiabdcfaqjabcIcaOiabdseaejabcYha8jabdIeaijabcMcaPiabdcfaqjabcIcaOiabdIeaijabcMcaPaqaamaaqababaGaemiuaaLaeiikaGIaemiraqKaeiiFaWNafmisaGKbaGaacqGGPaqkcqWGqbaucqGGOaakcuWGibasgaacaiabcMcaPaWcbaGafmisaGKbaGaaaeqaniabggHiLdaaaOGaeiOla4caaa@4D50@

For example, assume that we have prior information that precisely one site for WM *w *occurs in *s *and that the other bases in *s *were drawn from a background model *b*. For simplicity we will assume that under this background model *b*, each letter has a probability *b*_*α *_to be base *α*. In this situation all possible hypotheses are simply all possible locations *i *at which the binding site might start. If we have no information to suggest that the site is more likely to occur at some places than others we use an uniform prior *P*(*i*) = constant. The likelihood *P*(*D*|*i*) of the data, i.e. sequence *s*, given the corresponding hypothesis is given by the product of probabilities that the bases from 1 up to *i *derive from the background model, that the segment from *i *+ 1 through *i *+ *l *derives from the WM *w*, and that bases *i *+ *l *+ 1 through *L *again derive from the background model.

**Figure 1:**
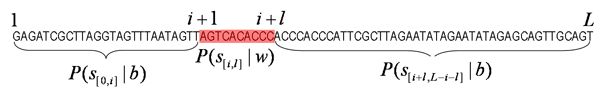
A dataset *D *consisting of a single sequence *s *of length *L*, with a single site hypothesized immediately after position *i*.

The probability *P*(*D*|*i*), as illustrated in Fig. 1, is given by

*P*(*D*|*i*) = *P*(*s*_[0,*i*]_|*b*)*P*(*s*_[*i*,*l*]_|*w*)*P*(*s*_[*i*+*l*,*L*-*i*-*l*]_|*b*),

where *s*_[*i*,*l*] _= *s*_*i*+1_*s*_*i*+2_...*s*_*i*+*l *_is the length-*l *segment in *s *starting after position *i *with

P(s[i,l]|w)=∏k=1lwsi+kk,
 MathType@MTEF@5@5@+=feaafiart1ev1aaatCvAUfKttLearuWrP9MDH5MBPbIqV92AaeXatLxBI9gBaebbnrfifHhDYfgasaacH8akY=wiFfYdH8Gipec8Eeeu0xXdbba9frFj0=OqFfea0dXdd9vqai=hGuQ8kuc9pgc9s8qqaq=dirpe0xb9q8qiLsFr0=vr0=vr0dc8meaabaqaciaacaGaaeqabaqabeGadaaakeaacqWGqbaucqGGOaakcqWGZbWCdaWgaaWcbaGaei4waSLaemyAaKMaeiilaWIaemiBaWMaeiyxa0fabeaakiabcYha8jabdEha3jabcMcaPiabg2da9maarahabaGaem4DaC3aa0baaSqaaiabdohaZnaaBaaameaacqWGPbqAcqGHRaWkcqWGRbWAaeqaaaWcbaGaem4AaSgaaaqaaiabdUgaRjabg2da9iabigdaXaqaaiabdYgaSbqdcqGHpis1aOGaeiilaWcaaa@4B51@

and the background probabilities are given by

P(s[0,i]|b)=∏k=1ibskP(s[i+l,L−i−l]|b)=∏k=i+l+1Lbsk.
 MathType@MTEF@5@5@+=feaafiart1ev1aaatCvAUfKttLearuWrP9MDH5MBPbIqV92AaeXatLxBI9gBaebbnrfifHhDYfgasaacH8akY=wiFfYdH8Gipec8Eeeu0xXdbba9frFj0=OqFfea0dXdd9vqai=hGuQ8kuc9pgc9s8qqaq=dirpe0xb9q8qiLsFr0=vr0=vr0dc8meaabaqaciaacaGaaeqabaqabeGadaaakeaafaqaceGabaaabaGaemiuaaLaeiikaGIaem4Cam3aaSbaaSqaaiabcUfaBjabicdaWiabcYcaSiabdMgaPjabc2faDbqabaGccqGG8baFcqWGIbGycqGGPaqkcqGH9aqpdaqeWbqaaiabdkgaInaaBaaaleaacqWGZbWCdaWgaaadbaGaem4AaSgabeaaaSqabaaabaGaem4AaSMaeyypa0JaeGymaedabaGaemyAaKganiabg+GivdaakeaacqWGqbaucqGGOaakcqWGZbWCdaWgaaWcbaGaei4waSLaemyAaKMaey4kaSIaemiBaWMaeiilaWIaemitaWKaeyOeI0IaemyAaKMaeyOeI0IaemiBaWMaeiyxa0fabeaakiabcYha8jabdkgaIjabcMcaPiabg2da9maarahabaGaemOyai2aaSbaaSqaaiabdohaZnaaBaaameaacqWGRbWAaeqaaaWcbeaaaeaacqWGRbWAcqGH9aqpcqWGPbqAcqGHRaWkcqWGSbaBcqGHRaWkcqaIXaqmaeaacqWGmbata0Gaey4dIunakiabc6caUaaaaaa@6BA6@

With the uniform prior, the posterior probability *P*(*i*|*D*) that the site occurs at *i *is

P(i|D=P(D|i)∑j=0L−lP(D|j).
 MathType@MTEF@5@5@+=feaafiart1ev1aaatCvAUfKttLearuWrP9MDH5MBPbIqV92AaeXatLxBI9gBaebbnrfifHhDYfgasaacH8akY=wiFfYdH8Gipec8Eeeu0xXdbba9frFj0=OqFfea0dXdd9vqai=hGuQ8kuc9pgc9s8qqaq=dirpe0xb9q8qiLsFr0=vr0=vr0dc8meaabaqaciaacaGaaeqabaqabeGadaaakeaacqWGqbaucqGGOaakcqWGPbqAcqGG8baFcqWGebarcqGH9aqpdaWcaaqaaiabdcfaqjabcIcaOiabdseaejabcYha8jabdMgaPjabcMcaPaqaamaaqadabaGaemiuaaLaeiikaGIaemiraqKaeiiFaWNaemOAaOMaeiykaKcaleaacqWGQbGAcqGH9aqpcqaIWaamaeaacqWGmbatcqGHsislcqWGSbaBa0GaeyyeIuoaaaGccqGGUaGlaaa@4AEF@

**Figure 2:**
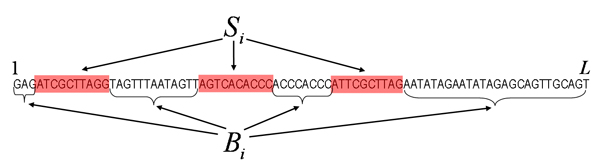
**A configuration *i *with 3 hypothesizes sites.***S*_*i *_denotes the set of hypothesized sites and *B*_*i *_the background bases.

In general we of course do not know that there is precisely one site in *s*. Therefore, we generally want to consider the extended set of hypotheses that consists of all possible configurations of binding sites that can be assigned to sequence *s*. Figure 2 shows a possible configuration containing 3 hypothesized binding sites.

Generally, each possible configuration with *n *sites can be denoted by a vector *i *= (*i*_1_, *i*_2_,...,*i*_*n*_) which denotes the positions at which the binding sites occur. The probability of the data given a configuration *i *is now given by

P(D|i)=[∏σ∈Bibσ]∏s∈SiP(s|w),
 MathType@MTEF@5@5@+=feaafiart1ev1aaatCvAUfKttLearuWrP9MDH5MBPbIqV92AaeXatLxBI9gBaebbnrfifHhDYfgasaacH8akY=wiFfYdH8Gipec8Eeeu0xXdbba9frFj0=OqFfea0dXdd9vqai=hGuQ8kuc9pgc9s8qqaq=dirpe0xb9q8qiLsFr0=vr0=vr0dc8meaabaqaciaacaGaaeqabaqabeGadaaakeaacqWGqbaucqGGOaakcqWGebarcqGG8baFcqWGPbqAcqGGPaqkcqGH9aqpdaWadaqaamaarafabaGaemOyai2aaSbaaSqaaGGaciab=n8aZbqabaaabaGae83WdmNaeyicI4SaemOqai0aaSbaaWqaaiabdMgaPbqabaaaleqaniabg+GivdaakiaawUfacaGLDbaadaqeqbqaaiabdcfaqjabcIcaOiabdohaZjabcYha8jabdEha3jabcMcaPiabcYcaSaWcbaGaem4CamNaeyicI4Saem4uam1aaSbaaWqaaiabdMgaPbqabaaaleqaniabg+Givdaaaa@5189@

where *B*_*i *_is the set of background bases and *S*_*i *_is the set of hypothesized sites in configuration *i*.

To assign prior probabilities *P*(*i*) to all possible configurations one generally assumes that the data *D *was produced through a stochastic process where at each step with probability (1 - *π*) a single background base is emitted, and with probability *π *a length-*l *binding site is emitted. Under this model the prior probability *P*(*i*) for a configuration *i *depends only on the number of sites *n*(*i*) that occurs in the configuration, and is given by

*P*(*i*) ∝ *π*^*n*(*i*) ^(1 - *π*)^*L*-*ln*(*i*)^

Using this the posterior probability of configuration *i *given the data becomes

P(i|D)=P(D|i)πn(i)(1−π)L−ln(i)∑jP(D|j)πn(j)(1−π)L−ln(j),
 MathType@MTEF@5@5@+=feaafiart1ev1aaatCvAUfKttLearuWrP9MDH5MBPbIqV92AaeXatLxBI9gBaebbnrfifHhDYfgasaacH8akY=wiFfYdH8Gipec8Eeeu0xXdbba9frFj0=OqFfea0dXdd9vqai=hGuQ8kuc9pgc9s8qqaq=dirpe0xb9q8qiLsFr0=vr0=vr0dc8meaabaqaciaacaGaaeqabaqabeGadaaakeaacqWGqbaucqGGOaakcqWGPbqAcqGG8baFcqWGebarcqGGPaqkcqGH9aqpdaWcaaqaaiabdcfaqjabcIcaOiabdseaejabcYha8jabdMgaPjabcMcaPGGaciab=b8aWnaaCaaaleqabaGaemOBa4MaeiikaGIaemyAaKMaeiykaKcaaOGaeiikaGIaeGymaeJaeyOeI0Iae8hWdaNaeiykaKYaaWbaaSqabeaacqWGmbatcqGHsislieGacqGFSbaBcqGFUbGBcqGGOaakcqWGPbqAcqGGPaqkaaaakeaadaaeqaqaaiabdcfaqjabcIcaOiabdseaejabcYha8jabdQgaQjabcMcaPiab=b8aWnaaCaaaleqabaGaemOBa4MaeiikaGIaemOAaOMaeiykaKcaaOGaeiikaGIaeGymaeJaeyOeI0Iae8hWdaNaeiykaKYaaWbaaSqabeaacqWGmbatcqGHsislcqGFSbaBcqGFUbGBcqGGOaakcqWGQbGAcqGGPaqkaaaabaGaemOAaOgabeqdcqGHris5aaaakiabcYcaSaaa@6DB5@

where the sum in the denominator is over all possible binding site configurations *j*.

Even though the total number of configurations grows faster than exponential with the sequence length *L*, the sum in the denominator can be easily calculated using dynamic programming as follows. Let *F*_*n *_denote the sum of the likelihoods of all configurations up to position *n *in *s*. We have the recurrence relation

**Figure 3:**
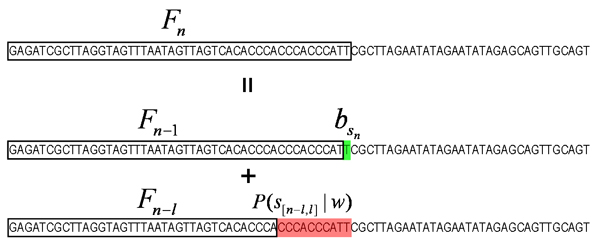
**Illustration of equation (16)**. The black rectangle indicates the sum *F*_*n *_of probabilities *P*(*D*|*i*) for all binding site configurations *i *for the sequence within the rectangle. Any configuration in *F*_*n *_is obtained either through adding a single background base at *n *to any of the configurations in *F*_*n *- 1_, or by adding a site from *n *- *l *+ 1 through *n *to any configuration in *F*_*n *- *l*_.

*F*_*n *_= *F*_*n*-1_(1 - *π*) bsn
 MathType@MTEF@5@5@+=feaafiart1ev1aaatCvAUfKttLearuWrP9MDH5MBPbIqV92AaeXatLxBI9gBaebbnrfifHhDYfgasaacH8akY=wiFfYdH8Gipec8Eeeu0xXdbba9frFj0=OqFfea0dXdd9vqai=hGuQ8kuc9pgc9s8qqaq=dirpe0xb9q8qiLsFr0=vr0=vr0dc8meaabaqaciaacaGaaeqabaqabeGadaaakeaacqWGIbGydaWgaaWcbaGaem4Cam3aaSbaaWqaaiabd6gaUbqabaaaleqaaaaa@3131@ + *F*_*n*-*l*_*πP*(*s*_[*n*-*l*, *l*]_|*w*),

as illustrated in Fig 3.

Notice that the sum over all configurations is just *F*_*L*_, i.e. *F*_*L *_= ∑_*j *_*P*(*D*|*j*)*P*(*j*), which can be calculated in a time *O*(*L*) using the above recurrence relation. Similarly, we can move backward from the end of the sequence to have a recurrence relation for the sum of likelihoods of all configurations of positions *n *through *L *of *s*:

*R*_*n *_= bsn
 MathType@MTEF@5@5@+=feaafiart1ev1aaatCvAUfKttLearuWrP9MDH5MBPbIqV92AaeXatLxBI9gBaebbnrfifHhDYfgasaacH8akY=wiFfYdH8Gipec8Eeeu0xXdbba9frFj0=OqFfea0dXdd9vqai=hGuQ8kuc9pgc9s8qqaq=dirpe0xb9q8qiLsFr0=vr0=vr0dc8meaabaqaciaacaGaaeqabaqabeGadaaakeaacqWGIbGydaWgaaWcbaGaem4Cam3aaSbaaWqaaiabd6gaUbqabaaaleqaaaaa@3131@ (1 - *π*)*R*_*n*+1 _+ *P*(*s*_[*n*-1, *l*]_|*w*)*πR*_*n*+*l*_.

Finally, instead of calculating the posterior *P*(*i*|*D*) for a particular configuration *i*, we can also calculate the posterior probability that a site occurs at a given position, independent of the rest of the configuration. Let us denote by {*n*} the set of all configurations that have a site at segment *s*_[*n*,*l*] _.The posterior probability *P*({*n*}|*D*) is given by the sum of posterior probabilities of all configurations in {*n*}, i.e.

P({n}|D)=∑i∈{n}P(i|D).
 MathType@MTEF@5@5@+=feaafiart1ev1aaatCvAUfKttLearuWrP9MDH5MBPbIqV92AaeXatLxBI9gBaebbnrfifHhDYfgasaacH8akY=wiFfYdH8Gipec8Eeeu0xXdbba9frFj0=OqFfea0dXdd9vqai=hGuQ8kuc9pgc9s8qqaq=dirpe0xb9q8qiLsFr0=vr0=vr0dc8meaabaqaciaacaGaaeqabaqabeGadaaakeaacqWGqbaucqGGOaakcqGG7bWEcqWGUbGBcqGG9bqFcqGG8baFcqWGebarcqGGPaqkcqGH9aqpdaaeqbqaaiabdcfaqjabcIcaOiabdMgaPjabcYha8jabdseaejabcMcaPaWcbaGaemyAaKMaeyicI4Saei4EaSNaemOBa4MaeiyFa0habeqdcqGHris5aOGaeiOla4caaa@489F@

It is easy to see that this sum can be expressed in terms of *F*_*n *_and *R*_*n *_as follows:

P({n}|D)=FnP(s[n,l]|w)πRn+l+1FL,
 MathType@MTEF@5@5@+=feaafiart1ev1aaatCvAUfKttLearuWrP9MDH5MBPbIqV92AaeXatLxBI9gBaebbnrfifHhDYfgasaacH8akY=wiFfYdH8Gipec8Eeeu0xXdbba9frFj0=OqFfea0dXdd9vqai=hGuQ8kuc9pgc9s8qqaq=dirpe0xb9q8qiLsFr0=vr0=vr0dc8meaabaqaciaacaGaaeqabaqabeGadaaakeaacqWGqbaucqGGOaakcqGG7bWEcqWGUbGBcqGG9bqFcqGG8baFcqWGebarcqGGPaqkcqGH9aqpdaWcaaqaaiabdAeagnaaBaaaleaacqWGUbGBaeqaaOGaemiuaaLaeiikaGIaem4Cam3aaSbaaSqaaiabcUfaBjabd6gaUjabcYcaSiabdYgaSjabc2faDbqabaGccqGG8baFcqWG3bWDcqGGPaqkiiGacqWFapaCcqWGsbGudaWgaaWcbaGaemOBa4Maey4kaSIaemiBaWMaey4kaSIaeGymaedabeaaaOqaaiabdAeagnaaBaaaleaacqWGmbataeqaaaaakiabcYcaSaaa@53CD@

where the numerator corresponds to the sum over all configurations that have a site at *s*_[*n*,*l*]_.

Here it is useful to note that, formally speaking, the model that we have introduced is a hidden Markov model and that the expressions (16), (17), and (19) are essentially the same as the so-called forward-backward algorithms of hidden Markov model theory [8,9]. Researchers with a background in statistical physics tend to think of *F*_*L *_as a *partition sum *and the recurrence relations are essentially what is known as the *transfer matrix *technique.

Given the WM *w *for a regulatory factor we may use equation (19) to scan any sequence *s *for positions at which functional binding sites for the factor are likely to occur. The likelihood of success of this procedure critically depends on the density of true sites in the input sequence *s*. That is, even in a sequence generated entirely from the background model, segments that are indistinguishable from binding sites will occur by chance at a certain rate. For example, let's assume that such chance 'binding site lookalikes' occur once every 500 bps on average, and assume that we are looking for between 1 and 3 functional binding sites in an intergenic region of length 250 which stems from a bacterial genome. In this case we expect less than 1 binding site to occur by chance, and so we will likely be able to accurately determine the location of the 1 to 3 functional sites. In contrast, assume we are looking for 1 to 3 functional sites in the introns and upstream regions of a human gene, which together might contain as many as 100,000 bps of non-coding DNA. It is clear that in this case the functional sites will 'drown' in a sea of about 200 binding site lookalikes.

At this point the reader may ask how *the cell *distinguishes functional binding sites from mere 'lookalikes'. Comparing equation (2) with (6) and (7), we see that *P*_bound_(*s*) can be written in terms of *P*(*s*|*w*) and *c*. In other words, two segments *s *and *s' *for which *P*(*s*|*w*) = *P*(*s'*|*w*) necessarily have *P*_bound_(*s*) = *P*_bound_(*s'*), and one may thus wonder why two segments that are equally likely to be bound by the regulatory factor are not equally functional. There are a number of reasons. First, in eukaryotic genomes DNA is wrapped up in chromatin and so different sites may have different accessibility to the regulatory factor. Second, binding of the regulator may by itself not guarantee functionality, i.e. a regulatory effect. A number of additional constraints typically have to be satisfied. The site may need to occur in the vicinity of specific other regulatory sites, e.g. to mediate interactions between different factors bound at the different sites. The site may need to occur at a particular distance from the basal promoter and in a particular orientation to be able to interact with the basal machinery, and other constraints currently not yet understood. When we want to look for functional sites in long sequences we thus generally have to use information that goes beyond the probabilities *P*(*s*|*w*) of the individual sequence segments given individual WMs *w*. One type of additional information that can be used is that in some cases functional binding sites are known to cluster on the genome. We now discuss approaches to incorporating this information.

### Finding clusters of binding sites: regulatory modules

It has been well-established that in higher eukaryotic organisms transcription regulation is often implemented through regulatory 'modules' in which multiple binding sites for multiple regulatory factors cluster together relatively tightly in intergenic regions [10]. In some cases one may even know the subsets of regulatory factors that tend to cooperate in regulatory modules for particular biological pathways. For example, a large body of work has identified the sets of transcription factors that are involved in segmentation of the early Drosophila embryo, e.g. see [11].

One approach to distinguishing functional binding sites from nonfunctional ones is to look for such regulatory modules. That is, the idea is to start with a set of WMs {*w*}, preferably from a set of regulatory factors that are believed to interact in regulatory modules, and to look for relatively short genomic segments in which there is a surprisingly high density of sites for the WMs from {*w*}. As far as this author is aware, this general idea was introduced around the same time by a number of groups [12-15]. The implementation we discuss here is most closely-related to the approaches of refs. [14,15].

The first thing to note is that the dynamic programming solution introduced in the previous section can be easily extended to multiple WMs *w *(potentially of different lengths). We now assume that the data is produced through a stochastic process where at each step with probability *π*_bg _a background base is generated, and with probability *π*_*w *_a WM segment from WM *w *with length *l*_*w *_is generated. The priors of course satisfy the normalization *π*_bg _+ ∑_*w*_*π*_*w *_= 1. For notational simplicity we can consider the background *b *to just be one of the WMs (with length *l *= 1) in the set {*w*}. In this more general model the recurrence relation for *F*_*n *_becomes

Fn=∑wFn−lwπwP(s[n−lw,lw]|w),
 MathType@MTEF@5@5@+=feaafiart1ev1aaatCvAUfKttLearuWrP9MDH5MBPbIqV92AaeXatLxBI9gBaebbnrfifHhDYfgasaacH8akY=wiFfYdH8Gipec8Eeeu0xXdbba9frFj0=OqFfea0dXdd9vqai=hGuQ8kuc9pgc9s8qqaq=dirpe0xb9q8qiLsFr0=vr0=vr0dc8meaabaqaciaacaGaaeqabaqabeGadaaakeaacqWGgbGrdaWgaaWcbaGaemOBa4gabeaakiabg2da9maaqafabaGaemOray0aaSbaaSqaaiabd6gaUjabgkHiTiabdYgaSnaaBaaameaacqWG3bWDaeqaaaWcbeaaiiGakiab=b8aWnaaBaaaleaacqWG3bWDaeqaaOGaemiuaaLaeiikaGIaem4Cam3aaSbaaSqaaiabcUfaBjabd6gaUjabgkHiTiabdYgaSnaaBaaameaacqWG3bWDaeqaaSGaeiilaWIaemiBaW2aaSbaaWqaaiabdEha3bqabaWccqGGDbqxaeqaaOGaeiiFaWNaem4DaCNaeiykaKcaleaacqWG3bWDaeqaniabggHiLdGccqGGSaalaaa@524D@

where the background *b *is now one of the WMs *w*.

The second thing to note is that the sum over all configurations *F*_*L *_= ∑_*c*_*P*(*D*|*c*)*P*(*c*|{*π*_*w*_}) is formally the likelihood of the data *D *under our entire set of hypotheses *c*, that is, it is the probability to obtain the data under the assumed stochastic model. Note that in this expression we have indicated explicitly that this probability depends on the priors {*π*_*w*_}. The quantity *F*_*L *_thus summarizes how well the sequence can be explained in terms of the set of WMs in the model. The basic idea of the regulatory module detecting algorithms in [14,15] is to identify putative regulatory modules with sequence segments that have a high value for the sum *F*_*L *_of probabilities of all binding site configurations in the segment.

The procedure works as follows. One starts with an intergenic region upstream of a gene of interest in a higher eukaryotic genome. Such intergenic regions are typically quite large, i.e. from 10 Kbps in flies to over 100 Kbps in humans. One then slides a windows of length between 200 and 500 bps or so over this long intergenic region. For each window one then determines the set of priors {*π*_*w*_} that maximize *F*_*L *_for the sequence *σ *in the window, and calculates the value of *F*_*L *_at this maximum. One also calculates the probability *P*(*σ*|*b*) for the sequence in the window deriving entirely from the background model. The ratio *X *= *F*_*L*_/*P*(*σ*|*b*) then quantifies the 'score' for the window in question. Finally, the predicted regulatory models are all windows for which *X *is larger than some prespecified cut-off, and for which the score *X *is larger than the score for any other window overlapping it.

A key step in this procedure is maximizing *F*_*L *_with respect to the prior {*π*_*w*_}. Different regulatory modules may have different densities of sites and we thus want to allow for different priors {*π*_*w*_} within different windows. Since we do not know the {*π*_*w*_} for each segment, from the point of view of probability theory one should strictly speaking not maximize with respect to {*π*_*w*_} but rather integrate over all possible priors {*π*_*w*_}. However, the resulting expressions no longer allow for an effective dynamic programming solution and this would thus make the problem computationally intractable. However, if the function *F*_*L *_has a sharp peak with respect to the {*π*_*w*_} then the height of the maximum is representative for the value of the integral and one can thus think of the maximization of *F*_*L *_with respect to the {*π*_*w*_} as an approximation to doing the full integral.

Assuming that segment *σ *is of length *L *the set of equations specifying the maximum with respect to the {*π*_*w*_} are

constant=dlog⁡(Z)dπw=dlog⁡(FL)dπw=〈n(w)〉πw,
 MathType@MTEF@5@5@+=feaafiart1ev1aaatCvAUfKttLearuWrP9MDH5MBPbIqV92AaeXatLxBI9gBaebbnrfifHhDYfgasaacH8akY=wiFfYdH8Gipec8Eeeu0xXdbba9frFj0=OqFfea0dXdd9vqai=hGuQ8kuc9pgc9s8qqaq=dirpe0xb9q8qiLsFr0=vr0=vr0dc8meaabaqaciaacaGaaeqabaqabeGadaaakeaatCvAUfeBSjuyZL2yd9gzLbvyNv2CaeHbuLwBLnhiov2DGi1BTfMBaGabaiaa=ngacaWFVbGaa8NBaiaa=nhacaWF0bGaa8xyaiaa=5gacaWF0bGaeyypa0ZaaSaaaeaacqWGKbazcyGGSbaBcqGGVbWBcqGGNbWzcqGGOaakcqWGAbGwcqGGPaqkaeaacqWGKbaziiGacqGFapaCdaWgaaWcbaGaem4DaChabeaaaaGccqGH9aqpdaWcaaqaaiabdsgaKjGbcYgaSjabc+gaVjabcEgaNjabcIcaOiabdAeagnaaBaaaleaacqWGmbataeqaaOGaeiykaKcabaGaemizaqMae4hWda3aaSbaaSqaaiabdEha3bqabaaaaOGaeyypa0ZaaSaaaeaacqGHPms4cqWGUbGBcqGGOaakcqWG3bWDcqGGPaqkcqGHQms8aeaacqGFapaCdaWgaaWcbaGaem4DaChabeaaaaGccqGGSaalaaa@6C49@

where <*n*(*w*)> is the expected number of binding sites for WM *w *averaged over all configurations, each weighted by its probability. The last equation follows from the fact that, the prior *P*(*c*|{*π*_*w*_}) is given by

P(c|{πw})=∏w(πw)n(w,c),
 MathType@MTEF@5@5@+=feaafiart1ev1aaatCvAUfKttLearuWrP9MDH5MBPbIqV92AaeXatLxBI9gBaebbnrfifHhDYfgasaacH8akY=wiFfYdH8Gipec8Eeeu0xXdbba9frFj0=OqFfea0dXdd9vqai=hGuQ8kuc9pgc9s8qqaq=dirpe0xb9q8qiLsFr0=vr0=vr0dc8meaabaqaciaacaGaaeqabaqabeGadaaakeaacqWGqbaucqGGOaakcqWGJbWycqGG8baFcqGG7bWEiiGacqWFapaCdaWgaaWcbaGaem4DaChabeaakiabc2ha9jabcMcaPiabg2da9maarafabaGaeiikaGIae8hWda3aaSbaaSqaaiabdEha3bqabaGccqGGPaqkdaahaaWcbeqaaiabd6gaUjabcIcaOiabdEha3jabcYcaSiabdogaJjabcMcaPaaaaeaacqWG3bWDaeqaniabg+GivdGccqGGSaalaaa@4A36@

where *n*(*w*, *c*) is the number of sites for *w *in configuration *c*. The derivative then becomes

d∑cP(σ|c)P(c|{πw})dπw=1πw∑cn(w,c)P(σ|c)P(c|{πw}).
 MathType@MTEF@5@5@+=feaafiart1ev1aaatCvAUfKttLearuWrP9MDH5MBPbIqV92AaeXatLxBI9gBaebbnrfifHhDYfgasaacH8akY=wiFfYdH8Gipec8Eeeu0xXdbba9frFj0=OqFfea0dXdd9vqai=hGuQ8kuc9pgc9s8qqaq=dirpe0xb9q8qiLsFr0=vr0=vr0dc8meaabaqaciaacaGaaeqabaqabeGadaaakeaadaWcaaqaaiabdsgaKnaaqababaGaemiuaaLaeiikaGccciGae83WdmNaeiiFaWNaem4yamMaeiykaKIaemiuaaLaeiikaGIaem4yamMaeiiFaWNaei4EaSNae8hWda3aaSbaaSqaaiabdEha3bqabaGccqGG9bqFcqGGPaqkaSqaaiabdogaJbqab0GaeyyeIuoaaOqaaiabdsgaKjab=b8aWnaaBaaaleaacqWG3bWDaeqaaaaakiabg2da9maalaaabaGaeGymaedabaGae8hWda3aaSbaaSqaaiabdEha3bqabaaaaOWaaabuaeaacqWGUbGBcqGGOaakcqWG3bWDcqGGSaalcqWGJbWycqGGPaqkcqWGqbaucqGGOaakcqWFdpWCcqGG8baFcqWGJbWycqGGPaqkcqWGqbaucqGGOaakcqWGJbWycqGG8baFcqGG7bWEcqWFapaCdaWgaaWcbaGaem4DaChabeaakiabc2ha9jabcMcaPaWcbaGaem4yamgabeqdcqGHris5aOGaeiOla4caaa@6D81@

Thus, from (21) and that fact that the *π*_*w *_are normalized to sum to 1 we have

πw=〈n(w)〉∑w˜〈n(w˜)〉.
 MathType@MTEF@5@5@+=feaafiart1ev1aaatCvAUfKttLearuWrP9MDH5MBPbIqV92AaeXatLxBI9gBaebbnrfifHhDYfgasaacH8akY=wiFfYdH8Gipec8Eeeu0xXdbba9frFj0=OqFfea0dXdd9vqai=hGuQ8kuc9pgc9s8qqaq=dirpe0xb9q8qiLsFr0=vr0=vr0dc8meaabaqaciaacaGaaeqabaqabeGadaaakeaaiiGacqWFapaCdaWgaaWcbaGaem4DaChabeaakiabg2da9maalaaabaGaeyykJeUaemOBa4MaeiikaGIaem4DaCNaeiykaKIaeyOkJepabaWaaabeaeaacqGHPms4cqWGUbGBcqGGOaakcuWG3bWDgaacaiabcMcaPiabgQYiXdWcbaGafm4DaCNbaGaaaeqaniabggHiLdaaaOGaeiOla4caaa@45BB@

Typically this maximum is found through *expectation maximization *(EM). Starting from an initial guess of the {*π*_*w*_} we calculate <*n*(*w*)> for all *w *and set a new set of priors {*π*_*w*_} using equation (24). Under iteration this is guaranteed to lead to an optimum in *F*_*L*_, although not necessarily the global optimum.

## Motif finding

Up to now we have assumed that we are in possession of the WMs *w *representing the sequence-specificities of the regulatory factors. However, unless one has experimental data that directly measures binding affinities of different sequence segments we generally do not possess such detailed information. Typically the best situation encountered is that we have a collection *S *of sequences that have been determined to be functional binding sites for the regulatory factor. So we now ask what we know about the WM *w *given such a set of sequences *S*, i.e. we aim to calculate *P*(*w*|*S*).

Equation (7) gives the probability that a binding site for *w *will have sequence *s*. This can be trivially extended to *sets *of sequences. That is, the probability to obtain the set of *n *length-*l *sequences *S *when sampling *n *sequences from the WM *w *is given by

P(S|w)=∏s∈SP(s|w)=∏i=1l∏α(wαi)nαi(S),
 MathType@MTEF@5@5@+=feaafiart1ev1aaatCvAUfKttLearuWrP9MDH5MBPbIqV92AaeXatLxBI9gBaebbnrfifHhDYfgasaacH8akY=wiFfYdH8Gipec8Eeeu0xXdbba9frFj0=OqFfea0dXdd9vqai=hGuQ8kuc9pgc9s8qqaq=dirpe0xb9q8qiLsFr0=vr0=vr0dc8meaabaqaciaacaGaaeqabaqabeGadaaakeaacqWGqbaucqGGOaakcqWGtbWucqGG8baFcqWG3bWDcqGGPaqkcqGH9aqpdaqeqbqaaiabdcfaqjabcIcaOiabdohaZjabcYha8jabdEha3jabcMcaPaWcbaGaem4CamNaeyicI4Saem4uamfabeqdcqGHpis1aOGaeyypa0ZaaebCaeaadaqeqbqaaiabcIcaOiabdEha3naaDaaaleaaiiGacqWFXoqyaeaacqWGPbqAaaGccqGGPaqkdaahaaWcbeqaaiabd6gaUnaaDaaameaacqWFXoqyaeaacqWGPbqAaaWccqGGOaakcqWGtbWucqGGPaqkaaaabaGae8xSdegabeqdcqGHpis1aaWcbaGaemyAaKMaeyypa0JaeGymaedabaGaemiBaWganiabg+GivdGccqGGSaalaaa@5CA8@

where in the last equation we have defined nαi
 MathType@MTEF@5@5@+=feaafiart1ev1aaatCvAUfKttLearuWrP9MDH5MBPbIqV92AaeXatLxBI9gBaebbnrfifHhDYfgasaacH8akY=wiFfYdH8Gipec8Eeeu0xXdbba9frFj0=OqFfea0dXdd9vqai=hGuQ8kuc9pgc9s8qqaq=dirpe0xb9q8qiLsFr0=vr0=vr0dc8meaabaqaciaacaGaaeqabaqabeGadaaakeaacqWGUbGBdaqhaaWcbaacciGae8xSdegabaGaemyAaKgaaaaa@313F@(*S*) as the number of times the letter *α *occurs at position *i *in the sequences *S*. Thus, the probability to obtain sequences *S *when sampling from the WM *w *depends only on the counts nαi
 MathType@MTEF@5@5@+=feaafiart1ev1aaatCvAUfKttLearuWrP9MDH5MBPbIqV92AaeXatLxBI9gBaebbnrfifHhDYfgasaacH8akY=wiFfYdH8Gipec8Eeeu0xXdbba9frFj0=OqFfea0dXdd9vqai=hGuQ8kuc9pgc9s8qqaq=dirpe0xb9q8qiLsFr0=vr0=vr0dc8meaabaqaciaacaGaaeqabaqabeGadaaakeaacqWGUbGBdaqhaaWcbaacciGae8xSdegabaGaemyAaKgaaaaa@313F@(*S*).

Using Bayes' theorem the posterior probability *P*(*w*|*S*) for the WM given the set of sites *S *is formally given by

P(w|S)=P(S|w)P(w)P(S).
 MathType@MTEF@5@5@+=feaafiart1ev1aaatCvAUfKttLearuWrP9MDH5MBPbIqV92AaeXatLxBI9gBaebbnrfifHhDYfgasaacH8akY=wiFfYdH8Gipec8Eeeu0xXdbba9frFj0=OqFfea0dXdd9vqai=hGuQ8kuc9pgc9s8qqaq=dirpe0xb9q8qiLsFr0=vr0=vr0dc8meaabaqaciaacaGaaeqabaqabeGadaaakeaacqWGqbaucqGGOaakcqWG3bWDcqGG8baFcqWGtbWucqGGPaqkcqGH9aqpdaWcaaqaaiabdcfaqjabcIcaOiabdofatjabcYha8jabdEha3jabcMcaPiabdcfaqjabcIcaOiabdEha3jabcMcaPaqaaiabdcfaqjabcIcaOiabdofatjabcMcaPaaacqGGUaGlaaa@4504@

In this equation *P*(*w*) is the *prior *probability that the WM is given by *w*. The denominator is a normalizing constant, which does not depend on the WM (we discuss its meaning in a minute). The prior *P*(*w*) represents our prior information about the WM *w *before we see any sites. As will become clear below, the computations are analytically most easily tractable if we use so-called Dirichlet priors that have the following general form

P(w)=∏i=1lP(wi)=∏i=1lci∏α(wαi)γαi−1,
 MathType@MTEF@5@5@+=feaafiart1ev1aaatCvAUfKttLearuWrP9MDH5MBPbIqV92AaeXatLxBI9gBaebbnrfifHhDYfgasaacH8akY=wiFfYdH8Gipec8Eeeu0xXdbba9frFj0=OqFfea0dXdd9vqai=hGuQ8kuc9pgc9s8qqaq=dirpe0xb9q8qiLsFr0=vr0=vr0dc8meaabaqaciaacaGaaeqabaqabeGadaaakeaacqWGqbaucqGGOaakcqWG3bWDcqGGPaqkcqGH9aqpdaqeWbqaaiabdcfaqjabcIcaOiabdEha3naaBaaaleaacqWGPbqAaeqaaOGaeiykaKcaleaacqWGPbqAcqGH9aqpcqaIXaqmaeaacqWGSbaBa0Gaey4dIunakiabg2da9maarahabaGaem4yam2aaSbaaSqaaiabdMgaPbqabaaabaGaemyAaKMaeyypa0JaeGymaedabaGaemiBaWganiabg+GivdGcdaqeqbqaaiabcIcaOiabdEha3naaDaaaleaaiiGacqWFXoqyaeaacqWGPbqAaaGccqGGPaqkdaahaaWcbeqaaiab=n7aNnaaDaaameaacqWFXoqyaeaacqWGPbqAaaWccqGHsislcqaIXaqmaaaabaGae8xSdegabeqdcqGHpis1aOGaeiilaWcaaa@5B58@

where *c*_*i *_is a normalization constant for column *i*, and the γαi
 MathType@MTEF@5@5@+=feaafiart1ev1aaatCvAUfKttLearuWrP9MDH5MBPbIqV92AaeXatLxBI9gBaebbnrfifHhDYfgasaacH8akY=wiFfYdH8Gipec8Eeeu0xXdbba9frFj0=OqFfea0dXdd9vqai=hGuQ8kuc9pgc9s8qqaq=dirpe0xb9q8qiLsFr0=vr0=vr0dc8meaabaqaciaacaGaaeqabaqabeGadaaakeaaiiGacqWFZoWzdaqhaaWcbaGae8xSdegabaGaemyAaKgaaaaa@317C@ are constants that determine the prior. Notice that for the particular choice γαi
 MathType@MTEF@5@5@+=feaafiart1ev1aaatCvAUfKttLearuWrP9MDH5MBPbIqV92AaeXatLxBI9gBaebbnrfifHhDYfgasaacH8akY=wiFfYdH8Gipec8Eeeu0xXdbba9frFj0=OqFfea0dXdd9vqai=hGuQ8kuc9pgc9s8qqaq=dirpe0xb9q8qiLsFr0=vr0=vr0dc8meaabaqaciaacaGaaeqabaqabeGadaaakeaaiiGacqWFZoWzdaqhaaWcbaGae8xSdegabaGaemyAaKgaaaaa@317C@ = 1 we obtain a *uniform *prior that makes all WMs a priori equally likely, which can be argued to reflect a state of complete ignorance about the WM. In reality, however, we know that for most positions in the site, regulatory factors tend to have distinct preferences for certain bases. That is, we a priori know that a WM column *w*^*i *^= (0.25, 0.25, 0.25, 0.25) is not very likely. To reflect this information we can choose γαi
 MathType@MTEF@5@5@+=feaafiart1ev1aaatCvAUfKttLearuWrP9MDH5MBPbIqV92AaeXatLxBI9gBaebbnrfifHhDYfgasaacH8akY=wiFfYdH8Gipec8Eeeu0xXdbba9frFj0=OqFfea0dXdd9vqai=hGuQ8kuc9pgc9s8qqaq=dirpe0xb9q8qiLsFr0=vr0=vr0dc8meaabaqaciaacaGaaeqabaqabeGadaaakeaaiiGacqWFZoWzdaqhaaWcbaGae8xSdegabaGaemyAaKgaaaaa@317C@ < 1. This will put more weight on WM columns that are 'skewed', i.e. giving low probability to some bases and high probabilities to others. Sometimes we have even more pertinent information. It has, for example, been argued recently that groups of related TFs show the same pattern of highly and less skewed columns [16]. If we are inferring the WM of such a TF we can thus reflect that information by setting γαi
 MathType@MTEF@5@5@+=feaafiart1ev1aaatCvAUfKttLearuWrP9MDH5MBPbIqV92AaeXatLxBI9gBaebbnrfifHhDYfgasaacH8akY=wiFfYdH8Gipec8Eeeu0xXdbba9frFj0=OqFfea0dXdd9vqai=hGuQ8kuc9pgc9s8qqaq=dirpe0xb9q8qiLsFr0=vr0=vr0dc8meaabaqaciaacaGaaeqabaqabeGadaaakeaaiiGacqWFZoWzdaqhaaWcbaGae8xSdegabaGaemyAaKgaaaaa@317C@ small for those positions *i *that are known to be highly skewed and γαi
 MathType@MTEF@5@5@+=feaafiart1ev1aaatCvAUfKttLearuWrP9MDH5MBPbIqV92AaeXatLxBI9gBaebbnrfifHhDYfgasaacH8akY=wiFfYdH8Gipec8Eeeu0xXdbba9frFj0=OqFfea0dXdd9vqai=hGuQ8kuc9pgc9s8qqaq=dirpe0xb9q8qiLsFr0=vr0=vr0dc8meaabaqaciaacaGaaeqabaqabeGadaaakeaaiiGacqWFZoWzdaqhaaWcbaGae8xSdegabaGaemyAaKgaaaaa@317C@ ≈ 1 for columns that are known not to be very skewed (for example because TFs of that family do not touch the DNA at that position).

With a Dirichlet prior of the form (27) equation (26) becomes

P(w|S)=C∏i=1l∏α(wαi)nαi(S)+γαi−1,
 MathType@MTEF@5@5@+=feaafiart1ev1aaatCvAUfKttLearuWrP9MDH5MBPbIqV92AaeXatLxBI9gBaebbnrfifHhDYfgasaacH8akY=wiFfYdH8Gipec8Eeeu0xXdbba9frFj0=OqFfea0dXdd9vqai=hGuQ8kuc9pgc9s8qqaq=dirpe0xb9q8qiLsFr0=vr0=vr0dc8meaabaqaciaacaGaaeqabaqabeGadaaakeaacqWGqbaucqGGOaakcqWG3bWDcqGG8baFcqWGtbWucqGGPaqkcqGH9aqpcqWGdbWqdaqeWbqaamaarafabaGaeiikaGIaem4DaC3aa0baaSqaaGGaciab=f7aHbqaaiabdMgaPbaakiabcMcaPmaaCaaaleqabaGaemOBa42aa0baaWqaaiab=f7aHbqaaiabdMgaPbaaliabcIcaOiabdofatjabcMcaPiabgUcaRiab=n7aNnaaDaaameaacqWFXoqyaeaacqWGPbqAaaWccqGHsislcqaIXaqmaaaabaGae8xSdegabeqdcqGHpis1aaWcbaGaemyAaKMaeyypa0JaeGymaedabaGaemiBaWganiabg+GivdGccqGGSaalaaa@56C1@

where *C *is an overall normalization constant. Equation (28) shows why the γαi
 MathType@MTEF@5@5@+=feaafiart1ev1aaatCvAUfKttLearuWrP9MDH5MBPbIqV92AaeXatLxBI9gBaebbnrfifHhDYfgasaacH8akY=wiFfYdH8Gipec8Eeeu0xXdbba9frFj0=OqFfea0dXdd9vqai=hGuQ8kuc9pgc9s8qqaq=dirpe0xb9q8qiLsFr0=vr0=vr0dc8meaabaqaciaacaGaaeqabaqabeGadaaakeaaiiGacqWFZoWzdaqhaaWcbaGae8xSdegabaGaemyAaKgaaaaa@317C@ are often called pseudocounts. Increasing γαi
 MathType@MTEF@5@5@+=feaafiart1ev1aaatCvAUfKttLearuWrP9MDH5MBPbIqV92AaeXatLxBI9gBaebbnrfifHhDYfgasaacH8akY=wiFfYdH8Gipec8Eeeu0xXdbba9frFj0=OqFfea0dXdd9vqai=hGuQ8kuc9pgc9s8qqaq=dirpe0xb9q8qiLsFr0=vr0=vr0dc8meaabaqaciaacaGaaeqabaqabeGadaaakeaaiiGacqWFZoWzdaqhaaWcbaGae8xSdegabaGaemyAaKgaaaaa@317C@ by 1 has the same effect on the posterior *P*(*w*|*S*) as adding 1 to the number of times nαi
 MathType@MTEF@5@5@+=feaafiart1ev1aaatCvAUfKttLearuWrP9MDH5MBPbIqV92AaeXatLxBI9gBaebbnrfifHhDYfgasaacH8akY=wiFfYdH8Gipec8Eeeu0xXdbba9frFj0=OqFfea0dXdd9vqai=hGuQ8kuc9pgc9s8qqaq=dirpe0xb9q8qiLsFr0=vr0=vr0dc8meaabaqaciaacaGaaeqabaqabeGadaaakeaacqWGUbGBdaqhaaWcbaacciGae8xSdegabaGaemyAaKgaaaaa@313F@(*S*) that letter *α *was observed at position *i*. Put another way, the posterior *P*(*w*|*S*) has exactly the same functional form as the prior *P*(*w*), i.e. both are of the form ∏α(wα)xα
MathType@MTEF@5@5@+=feaafiart1ev1aaatCvAUfKttLearuWrP9MDH5MBPbIqV92AaeXatLxBI9gBaebbnrfifHhDYfgasaacH8akY=wiFfYdH8Gipec8Eeeu0xXdbba9frFj0=OqFfea0dXdd9vqai=hGuQ8kuc9pgc9s8qqaq=dirpe0xb9q8qiLsFr0=vr0=vr0dc8meaabaqaciaacaGaaeqabaqabeGadaaakeaadaqeqaqaaiabcIcaOiabdEha3naaBaaaleaaiiGacqWFXoqyaeqaaOGaeiykaKYaaWbaaSqabeaacqWG4baEdaWgaaadbaGae8xSdegabeaaaaaaleaacqWFXoqyaeqaniabg+Givdaaaa@388A@ with *x*_*α *_the 'count' of base *α*. Priors that have this property are called *conjugate *priors. In this particular case it means that one may think of the posterior *P*(*w*|*S*) as the prior for another problem with 'pseudocounts' γ˜αi=nαi(S)+γαi
 MathType@MTEF@5@5@+=feaafiart1ev1aaatCvAUfKttLearuWrP9MDH5MBPbIqV92AaeXatLxBI9gBaebbnrfifHhDYfgasaacH8akY=wiFfYdH8Gipec8Eeeu0xXdbba9frFj0=OqFfea0dXdd9vqai=hGuQ8kuc9pgc9s8qqaq=dirpe0xb9q8qiLsFr0=vr0=vr0dc8meaabaqaciaacaGaaeqabaqabeGadaaakeaaiiGacuWFZoWzgaacamaaDaaaleaacqWFXoqyaeaacqWGPbqAaaGccqGH9aqpcqWGUbGBdaqhaaWcbaGae8xSdegabaGaemyAaKgaaOGaeiikaGIaem4uamLaeiykaKIaey4kaSIae83SdC2aa0baaSqaaiab=f7aHbqaaiabdMgaPbaaaaa@3FB3@. How to use the distribution *P*(*w*|*S*) in practice? In order to estimate the WM one could for example determine the WM *w *that maximizes *P*(*w*|*S*). This maximum posterior probability WM has components

wαi=nαi(S)+γαi−1ni(S)+γi−4,
 MathType@MTEF@5@5@+=feaafiart1ev1aaatCvAUfKttLearuWrP9MDH5MBPbIqV92AaeXatLxBI9gBaebbnrfifHhDYfgasaacH8akY=wiFfYdH8Gipec8Eeeu0xXdbba9frFj0=OqFfea0dXdd9vqai=hGuQ8kuc9pgc9s8qqaq=dirpe0xb9q8qiLsFr0=vr0=vr0dc8meaabaqaciaacaGaaeqabaqabeGadaaakeaacqWG3bWDdaqhaaWcbaacciGae8xSdegabaGaemyAaKgaaOGaeyypa0ZaaSaaaeaacqWGUbGBdaqhaaWcbaGae8xSdegabaGaemyAaKgaaOGaeiikaGIaem4uamLaeiykaKIaey4kaSIae83SdC2aa0baaSqaaiab=f7aHbqaaiabdMgaPbaakiabgkHiTiabigdaXaqaaiabd6gaUnaaCaaaleqabaGaemyAaKgaaOGaeiikaGIaem4uamLaeiykaKIaey4kaSIae83SdC2aaWbaaSqabeaacqWGPbqAaaGccqGHsislcqaI0aanaaGaeiilaWcaaa@4E21@

with *n*^*i*^(*S*) = ∑_*α *_nαi
 MathType@MTEF@5@5@+=feaafiart1ev1aaatCvAUfKttLearuWrP9MDH5MBPbIqV92AaeXatLxBI9gBaebbnrfifHhDYfgasaacH8akY=wiFfYdH8Gipec8Eeeu0xXdbba9frFj0=OqFfea0dXdd9vqai=hGuQ8kuc9pgc9s8qqaq=dirpe0xb9q8qiLsFr0=vr0=vr0dc8meaabaqaciaacaGaaeqabaqabeGadaaakeaacqWGUbGBdaqhaaWcbaacciGae8xSdegabaGaemyAaKgaaaaa@313F@(*S*) and *γ*^*i *^= ∑_*α*_γαi
 MathType@MTEF@5@5@+=feaafiart1ev1aaatCvAUfKttLearuWrP9MDH5MBPbIqV92AaeXatLxBI9gBaebbnrfifHhDYfgasaacH8akY=wiFfYdH8Gipec8Eeeu0xXdbba9frFj0=OqFfea0dXdd9vqai=hGuQ8kuc9pgc9s8qqaq=dirpe0xb9q8qiLsFr0=vr0=vr0dc8meaabaqaciaacaGaaeqabaqabeGadaaakeaaiiGacqWFZoWzdaqhaaWcbaGae8xSdegabaGaemyAaKgaaaaa@317C@. Note that with a uniform prior γαi
 MathType@MTEF@5@5@+=feaafiart1ev1aaatCvAUfKttLearuWrP9MDH5MBPbIqV92AaeXatLxBI9gBaebbnrfifHhDYfgasaacH8akY=wiFfYdH8Gipec8Eeeu0xXdbba9frFj0=OqFfea0dXdd9vqai=hGuQ8kuc9pgc9s8qqaq=dirpe0xb9q8qiLsFr0=vr0=vr0dc8meaabaqaciaacaGaaeqabaqabeGadaaakeaaiiGacqWFZoWzdaqhaaWcbaGae8xSdegabaGaemyAaKgaaaaa@317C@ = 1 the maximum occurs when the WM entries match the observed frequencies. This means, for example, that if a given base *α *is not observed at all at some position *i*, i.e. nαi
 MathType@MTEF@5@5@+=feaafiart1ev1aaatCvAUfKttLearuWrP9MDH5MBPbIqV92AaeXatLxBI9gBaebbnrfifHhDYfgasaacH8akY=wiFfYdH8Gipec8Eeeu0xXdbba9frFj0=OqFfea0dXdd9vqai=hGuQ8kuc9pgc9s8qqaq=dirpe0xb9q8qiLsFr0=vr0=vr0dc8meaabaqaciaacaGaaeqabaqabeGadaaakeaacqWGUbGBdaqhaaWcbaacciGae8xSdegabaGaemyAaKgaaaaa@313F@(*S*) = 0, we will assume that it is *impossible *for *α *to occur at position *i*. This is true even if the set *S *contains only very few sites.

Alternatively we may estimate the wαi
 MathType@MTEF@5@5@+=feaafiart1ev1aaatCvAUfKttLearuWrP9MDH5MBPbIqV92AaeXatLxBI9gBaebbnrfifHhDYfgasaacH8akY=wiFfYdH8Gipec8Eeeu0xXdbba9frFj0=OqFfea0dXdd9vqai=hGuQ8kuc9pgc9s8qqaq=dirpe0xb9q8qiLsFr0=vr0=vr0dc8meaabaqaciaacaGaaeqabaqabeGadaaakeaacqWG3bWDdaqhaaWcbaacciGae8xSdegabaGaemyAaKgaaaaa@3151@ by their expected values under the distribution *P*(*w*|*S*). To calculate these expectation values we have to integrate *P*(*w*|*S*) over all possible WMs. That is, for each position *i *the integral is over the simplex:

∑αwαi=1,wαi≥0∀α.
 MathType@MTEF@5@5@+=feaafiart1ev1aaatCvAUfKttLearuWrP9MDH5MBPbIqV92AaeXatLxBI9gBaebbnrfifHhDYfgasaacH8akY=wiFfYdH8Gipec8Eeeu0xXdbba9frFj0=OqFfea0dXdd9vqai=hGuQ8kuc9pgc9s8qqaq=dirpe0xb9q8qiLsFr0=vr0=vr0dc8meaabaqaciaacaGaaeqabaqabeGadaaakeaadaaeqbqaaiabdEha3naaDaaaleaaiiGacqWFXoqyaeaacqWGPbqAaaGccqGH9aqpcqaIXaqmcqGGSaalcqWG3bWDdaqhaaWcbaGae8xSdegabaGaemyAaKgaaOGaeyyzImRaeGimaaJaeyiaIiIae8xSdegaleaacqWFXoqyaeqaniabggHiLdGccqGGUaGlaaa@429D@

The solution to such integrals is given by the following general identity

∫(w1)x1−1...(wn)xn−1dw1...dwn=∏i=1nΓ(xi)Γ(∑i=1nxi),
MathType@MTEF@5@5@+=feaafiart1ev1aaatCvAUfKttLearuWrP9MDH5MBPbIqV92AaeXatLxBI9gBaebbnrfifHhDYfgasaacH8akY=wiFfYdH8Gipec8Eeeu0xXdbba9frFj0=OqFfea0dXdd9vqai=hGuQ8kuc9pgc9s8qqaq=dirpe0xb9q8qiLsFr0=vr0=vr0dc8meaabaqaciaacaGaaeqabaqabeGadaaakeaadaWdbaqaaiabcIcaOiabdEha3naaBaaaleaacqaIXaqmaeqaaOGaeiykaKYaaWbaaSqabeaacqWG4baEdaWgaaadbaGaeGymaedabeaaliabgkHiTiabigdaXaaaaeqabeqdcqGHRiI8aOGaeiOla4IaeiOla4IaeiOla4IaeiikaGIaem4DaC3aaSbaaSqaaiabd6gaUbqabaGccqGGPaqkdaahaaWcbeqaaiabdIha4naaBaaameaacqWGUbGBaeqaaSGaeyOeI0IaeGymaedaaOGaemizaqMaem4DaC3aaSbaaSqaaiabigdaXaqabaGccqGGUaGlcqGGUaGlcqGGUaGlcqWGKbazcqWG3bWDdaWgaaWcbaGaemOBa4gabeaakiabg2da9maalaaabaWaaebmaeaacqqHtoWrcqGGOaakcqWG4baEdaWgaaWcbaGaemyAaKgabeaakiabcMcaPaWcbaGaemyAaKMaeyypa0JaeGymaedabaGaemOBa4ganiabg+GivdaakeaacqqHtoWrcqGGOaakdaaeWaqaaiabdIha4naaBaaaleaacqWGPbqAaeqaaaqaaiabdMgaPjabg2da9iabigdaXaqaaiabd6gaUbqdcqGHris5aOGaeiykaKcaaiabcYcaSaaa@6AD3@

where the integral is over the simplex ∑i=1nwi=1
 MathType@MTEF@5@5@+=feaafiart1ev1aaatCvAUfKttLearuWrP9MDH5MBPbIqV92AaeXatLxBI9gBaebbnrfifHhDYfgasaacH8akY=wiFfYdH8Gipec8Eeeu0xXdbba9frFj0=OqFfea0dXdd9vqai=hGuQ8kuc9pgc9s8qqaq=dirpe0xb9q8qiLsFr0=vr0=vr0dc8meaabaqaciaacaGaaeqabaqabeGadaaakeaadaaeWaqaaiabdEha3naaBaaaleaacqWGPbqAaeqaaOGaeyypa0JaeGymaedaleaacqWGPbqAcqGH9aqpcqaIXaqmaeaacqWGUbGBa0GaeyyeIuoaaaa@3862@. Using this identity we first find the normalization constant of equation (28). That is, by demanding that ∫*P*(*w*|*S*)*dw *= 1 we obtain

1C=∏i=1l∏αΓ(nαi(S)+γαi)Γ(ni(S)+γi),
MathType@MTEF@5@5@+=feaafiart1ev1aaatCvAUfKttLearuWrP9MDH5MBPbIqV92AaeXatLxBI9gBaebbnrfifHhDYfgasaacH8akY=wiFfYdH8Gipec8Eeeu0xXdbba9frFj0=OqFfea0dXdd9vqai=hGuQ8kuc9pgc9s8qqaq=dirpe0xb9q8qiLsFr0=vr0=vr0dc8meaabaqaciaacaGaaeqabaqabeGadaaakeaadaWcaaqaaiabigdaXaqaaiabdoeadbaacqGH9aqpdaqeWbqaamaalaaabaWaaebeaeaacqqHtoWrcqGGOaakcqWGUbGBdaqhaaWcbaacciGae8xSdegabaGaemyAaKgaaOGaeiikaGIaem4uamLaeiykaKIaey4kaSIae83SdC2aa0baaSqaaiab=f7aHbqaaiabdMgaPbaakiabcMcaPaWcbaGae8xSdegabeqdcqGHpis1aaGcbaGaeu4KdCKaeiikaGIaemOBa42aaWbaaSqabeaacqWGPbqAaaGccqGGOaakcqWGtbWucqGGPaqkcqGHRaWkcqWFZoWzdaahaaWcbeqaaiabdMgaPbaakiabcMcaPaaaaSqaaiabdMgaPjabg2da9iabigdaXaqaaiabdYgaSbqdcqGHpis1aOGaeiilaWcaaa@5864@

and using this (plus the general identity Γ(*x *+ 1) = *x*Γ(*x*)) we find for the expectation values

〈wαi〉=∫wαiP(w|S)dw=nαi(S)+γαini(S)+γi.
 MathType@MTEF@5@5@+=feaafiart1ev1aaatCvAUfKttLearuWrP9MDH5MBPbIqV92AaeXatLxBI9gBaebbnrfifHhDYfgasaacH8akY=wiFfYdH8Gipec8Eeeu0xXdbba9frFj0=OqFfea0dXdd9vqai=hGuQ8kuc9pgc9s8qqaq=dirpe0xb9q8qiLsFr0=vr0=vr0dc8meaabaqaciaacaGaaeqabaqabeGadaaakeaacqGHPms4cqWG3bWDdaqhaaWcbaacciGae8xSdegabaGaemyAaKgaaOGaeyOkJeVaeyypa0Zaa8qaaeaacqWG3bWDdaqhaaWcbaGae8xSdegabaGaemyAaKgaaOGaemiuaaLaeiikaGIaem4DaCNaeiiFaWNaem4uamLaeiykaKIaemizaqMaem4DaChaleqabeqdcqGHRiI8aOGaeyypa0ZaaSaaaeaacqWGUbGBdaqhaaWcbaGae8xSdegabaGaemyAaKgaaOGaeiikaGIaem4uamLaeiykaKIaey4kaSIae83SdC2aa0baaSqaaiab=f7aHbqaaiabdMgaPbaaaOqaaiabd6gaUnaaCaaaleqabaGaemyAaKgaaOGaeiikaGIaem4uamLaeiykaKIaey4kaSIae83SdC2aaWbaaSqabeaacqWGPbqAaaaaaOGaeiOla4caaa@5F5F@

Note that in this estimate of the wαi
 MathType@MTEF@5@5@+=feaafiart1ev1aaatCvAUfKttLearuWrP9MDH5MBPbIqV92AaeXatLxBI9gBaebbnrfifHhDYfgasaacH8akY=wiFfYdH8Gipec8Eeeu0xXdbba9frFj0=OqFfea0dXdd9vqai=hGuQ8kuc9pgc9s8qqaq=dirpe0xb9q8qiLsFr0=vr0=vr0dc8meaabaqaciaacaGaaeqabaqabeGadaaakeaacqWG3bWDdaqhaaWcbaacciGae8xSdegabaGaemyAaKgaaaaa@3151@ no component gets probability zero if we use a prior with γαi
 MathType@MTEF@5@5@+=feaafiart1ev1aaatCvAUfKttLearuWrP9MDH5MBPbIqV92AaeXatLxBI9gBaebbnrfifHhDYfgasaacH8akY=wiFfYdH8Gipec8Eeeu0xXdbba9frFj0=OqFfea0dXdd9vqai=hGuQ8kuc9pgc9s8qqaq=dirpe0xb9q8qiLsFr0=vr0=vr0dc8meaabaqaciaacaGaaeqabaqabeGadaaakeaaiiGacqWFZoWzdaqhaaWcbaGae8xSdegabaGaemyAaKgaaaaa@317C@ > 0 for all *i *and *α*.

In the previous section we repeatedly made use of the expression *P*(*s*|*w*), i.e. the probability to obtain sequence *s *when sampling from the WM. We now calculate an analogous expression *P*(*s*|*S*) = ∫ *P*(*s*|*w*)*P*(*w*|*S*)*dw*, which is the probability to obtain sequence *s *when sampling from the same WM as the one from which the set *S *derived (without ever specifying precisely what this WM is, i.e. we integrate over all possible *w*). Using again the general identity (31) we obtain

P(s|S)=∏i=1lnsii(S)+γsiini(S)+γi=∏i=1l〈wsii〉.
 MathType@MTEF@5@5@+=feaafiart1ev1aaatCvAUfKttLearuWrP9MDH5MBPbIqV92AaeXatLxBI9gBaebbnrfifHhDYfgasaacH8akY=wiFfYdH8Gipec8Eeeu0xXdbba9frFj0=OqFfea0dXdd9vqai=hGuQ8kuc9pgc9s8qqaq=dirpe0xb9q8qiLsFr0=vr0=vr0dc8meaabaqaciaacaGaaeqabaqabeGadaaakeaacqWGqbaucqGGOaakcqWGZbWCcqGG8baFcqWGtbWucqGGPaqkcqGH9aqpdaqeWbqaamaalaaabaGaemOBa42aa0baaSqaaiabdohaZnaaBaaameaacqWGPbqAaeqaaaWcbaGaemyAaKgaaOGaeiikaGIaem4uamLaeiykaKIaey4kaSccciGae83SdC2aa0baaSqaaiabdohaZnaaBaaameaacqWGPbqAaeqaaaWcbaGaemyAaKgaaaGcbaGaemOBa42aaWbaaSqabeaacqWGPbqAaaGccqGGOaakcqWGtbWucqGGPaqkcqGHRaWkcqWFZoWzdaahaaWcbeqaaiabdMgaPbaaaaaabaGaemyAaKMaeyypa0JaeGymaedabaGaemiBaWganiabg+GivdGccqGH9aqpdaqeWbqaaiabgMYiHlabdEha3naaDaaaleaacqWGZbWCdaWgaaadbaGaemyAaKgabeaaaSqaaiabdMgaPbaakiabgQYiXdWcbaGaemyAaKMaeyypa0JaeGymaedabaGaemiBaWganiabg+GivdGccqGGUaGlaaa@67E4@

That is, we find that *P*(*s*|*S*) is precisely the probability that would be obtained from expression *P*(*s*|*w*) when using the expectation values <wαi
 MathType@MTEF@5@5@+=feaafiart1ev1aaatCvAUfKttLearuWrP9MDH5MBPbIqV92AaeXatLxBI9gBaebbnrfifHhDYfgasaacH8akY=wiFfYdH8Gipec8Eeeu0xXdbba9frFj0=OqFfea0dXdd9vqai=hGuQ8kuc9pgc9s8qqaq=dirpe0xb9q8qiLsFr0=vr0=vr0dc8meaabaqaciaacaGaaeqabaqabeGadaaakeaacqWG3bWDdaqhaaWcbaacciGae8xSdegabaGaemyAaKgaaaaa@3151@> as an estimate for the WM *w*.

Up to now we assumed that we were given a set *S *of length-*l *sequences that were sampled from the WM. Except in cases where we have, for example DNase footprinting data that give the precise locations of the regulatory sites, such specific data are again generally rare. It is much more common that we have a set of *n *longer sequences that we know (or strongly suspect) to contain one (or more) regulatory site(s) each for a common regulatory factor. In this situation we simultaneously need to infer *where *in the sequences the sites occur and what the WM is from which they derive.

To be explicit, let's assume we have a dataset *D *that consists of *n *length-*L *sequences, and we know that each sequence contains precisely one binding site of length *l *for a common regulatory factor. The set of hypotheses for this problem then corresponds to all combinations (*w*,*i*) of a WM *w *and a vector *i *= (*i*_1_, *i*_2_,... *i*_*n*_) that denotes the positions where the regulatory sites occur, i.e *i*_1 _is the position of the site in the first sequence, *i*_2 _the position of the site in the second sequence, etcetera. We now first calculate the probability *P*(*D*|*w*, *i*) of the data given (*w*, *i*). Let *S*_*i *_denote the set of *n *length-*l *segments that make up the hypothesized binding sites with positions *i *and let *B*_*i *_denote all background nucleotides in the data *D outside *of these segments. In analogy with equation (13) the probability *P*(*D*|*w*, *i*) is then given by

P(D|w,i)=[∏σ∈Bibσ]∏s∈SiP(s|w),
 MathType@MTEF@5@5@+=feaafiart1ev1aaatCvAUfKttLearuWrP9MDH5MBPbIqV92AaeXatLxBI9gBaebbnrfifHhDYfgasaacH8akY=wiFfYdH8Gipec8Eeeu0xXdbba9frFj0=OqFfea0dXdd9vqai=hGuQ8kuc9pgc9s8qqaq=dirpe0xb9q8qiLsFr0=vr0=vr0dc8meaabaqaciaacaGaaeqabaqabeGadaaakeaacqWGqbaucqGGOaakcqWGebarcqGG8baFcqWG3bWDcqGGSaalcqWGPbqAcqGGPaqkcqGH9aqpdaWadaqaamaarafabaGaemOyai2aaSbaaSqaaGGaciab=n8aZbqabaaabaGae83WdmNaeyicI4SaemOqai0aaSbaaWqaaiabdMgaPbqabaaaleqaniabg+GivdaakiaawUfacaGLDbaadaqeqbqaaiabdcfaqjabcIcaOiabdohaZjabcYha8jabdEha3jabcMcaPaWcbaGaem4CamNaeyicI4Saem4uam1aaSbaaWqaaiabdMgaPbqabaaaleqaniabg+GivdGccqGGSaalaaa@53EA@

where the first product is over all nucleotides outside of the hypothesized binding sites, and the second product is over all hypothesized binding sites *s*.

At this point there are two possible approaches. In the first approach one calculates the probability *P*(*D*|*w*) of the data given the weight matrix only by summing over all possible binding site configurations *i*:

P(D|w)=∑iP(D|w,i)P(i),
 MathType@MTEF@5@5@+=feaafiart1ev1aaatCvAUfKttLearuWrP9MDH5MBPbIqV92AaeXatLxBI9gBaebbnrfifHhDYfgasaacH8akY=wiFfYdH8Gipec8Eeeu0xXdbba9frFj0=OqFfea0dXdd9vqai=hGuQ8kuc9pgc9s8qqaq=dirpe0xb9q8qiLsFr0=vr0=vr0dc8meaabaqaciaacaGaaeqabaqabeGadaaakeaacqWGqbaucqGGOaakcqWGebarcqGG8baFcqWG3bWDcqGGPaqkcqGH9aqpdaaeqbqaaiabdcfaqjabcIcaOiabdseaejabcYha8jabdEha3jabcYcaSiabdMgaPjabcMcaPiabdcfaqjabcIcaOiabdMgaPjabcMcaPaWcbaGaemyAaKgabeqdcqGHris5aOGaeiilaWcaaa@4651@

where *P*(*i*) is a prior probability distribution over vectors of site assignments, and the sum is over all possible vectors. One then next searches the space of all possible WMs *w *for those with high *P*(*D*|*w*). In the second approach one calculates the probability *P*(*D*|*i*) of the data given the vector of site positions only by integrating over all possible weight matrices. Formally [6] this probability is given by

*P*(*D*|*i*) = ∫ *P*(*D*, *w*|*i*)*dw *= ∫ *P*(*D*|*w*, *i*)*P*(*w*)*dw*,

and next the set of all site positions *i *is searched for those with high *P*(*D*|*i*). We now discuss these approaches in turn.

### Maximizing *P*(*D*|*w*) through Expectation Maximization

In the first approach one attempts to find the weight matrix *w *that maximizes the probability of the data *P*(*D*|*w*). Note that, as we have seen in section "Finding WM matches", the sum over all possible site configurations *i *can be easily performed through dynamic programming once the matrix *w *is given. For the particular case we are considering, i.e. assuming precisely one site per sequence, the probability *P*(*D*|*w*) is given by the product of the probabilities for the individual sequences

P(D|w)=∏m=1nP(Dm|w),
 MathType@MTEF@5@5@+=feaafiart1ev1aaatCvAUfKttLearuWrP9MDH5MBPbIqV92AaeXatLxBI9gBaebbnrfifHhDYfgasaacH8akY=wiFfYdH8Gipec8Eeeu0xXdbba9frFj0=OqFfea0dXdd9vqai=hGuQ8kuc9pgc9s8qqaq=dirpe0xb9q8qiLsFr0=vr0=vr0dc8meaabaqaciaacaGaaeqabaqabeGadaaakeaacqWGqbaucqGGOaakcqWGebarcqGG8baFcqWG3bWDcqGGPaqkcqGH9aqpdaqeWbqaaiabdcfaqjabcIcaOiabdseaenaaBaaaleaacqWGTbqBaeqaaOGaeiiFaWNaem4DaCNaeiykaKcaleaacqWGTbqBcqGH9aqpcqaIXaqmaeaacqWGUbGBa0Gaey4dIunakiabcYcaSaaa@44EA@

with

P(Dm|w)=1L−l+1∑im[P(s[im,l]|w)∏σ∉s[im,l]bσ],
 MathType@MTEF@5@5@+=feaafiart1ev1aaatCvAUfKttLearuWrP9MDH5MBPbIqV92AaeXatLxBI9gBaebbnrfifHhDYfgasaacH8akY=wiFfYdH8Gipec8Eeeu0xXdbba9frFj0=OqFfea0dXdd9vqai=hGuQ8kuc9pgc9s8qqaq=dirpe0xb9q8qiLsFr0=vr0=vr0dc8meaabaqaciaacaGaaeqabaqabeGadaaakeaacqWGqbaucqGGOaakcqWGebardaWgaaWcbaGaemyBa0gabeaakiabcYha8jabdEha3jabcMcaPiabg2da9maalaaabaGaeGymaedabaGaemitaWKaeyOeI0IaemiBaWMaey4kaSIaeGymaedaamaaqafabaWaamWaaeaacqWGqbaucqGGOaakcqWGZbWCdaWgaaWcbaGaei4waSLaemyAaK2aaSbaaWqaaiabd2gaTbqabaWccqGGSaalcqWGSbaBcqGGDbqxaeqaaOGaeiiFaWNaem4DaCNaeiykaKYaaebuaeaacqWGIbGydaWgaaWcbaacciGae83WdmhabeaaaeaacqWFdpWCcqGHjiYZcqWGZbWCdaWgaaadbaGaei4waSLaemyAaK2aaSbaaeaacqWGTbqBaeqaaiabcYcaSiabdYgaSjabc2faDbqabaaaleqaniabg+GivdaakiaawUfacaGLDbaaaSqaaiabdMgaPnaaBaaameaacqWGTbqBaeqaaaWcbeqdcqGHris5aOGaeiilaWcaaa@6571@

where *D*_*m *_is the *m*th sequence, the product over *σ *is over all bases outside of the site (i.e. the background), and we have used the uniform prior *P*(*i*_*m*_) = 1/(*L *- *l *+ 1) over the binding binding site position *i*_*m*_.

To find the WM *w *that maximizes *P*(*D*|*w*) we proceed analogously as we did for finding the set of priors {*π*_*w*_} in equations (21) through (24). For each column *k *of the WM we have the four equations

constant=dlog⁡[P(D|w)]dwαk=〈nαk〉wαk,
 MathType@MTEF@5@5@+=feaafiart1ev1aaatCvAUfKttLearuWrP9MDH5MBPbIqV92AaeXatLxBI9gBaebbnrfifHhDYfgasaacH8akY=wiFfYdH8Gipec8Eeeu0xXdbba9frFj0=OqFfea0dXdd9vqai=hGuQ8kuc9pgc9s8qqaq=dirpe0xb9q8qiLsFr0=vr0=vr0dc8meaabaqaciaacaGaaeqabaqabeGadaaakeaaieaacqWFJbWycqWFVbWBcqWFUbGBcqWFZbWCcqWF0baDcqWFHbqycqWFUbGBcqWF0baDcqGH9aqpdaWcaaqaaiabdsgaKjGbcYgaSjabc+gaVjabcEgaNjabcUfaBjabdcfaqjabcIcaOiabdseaejabcYha8jabdEha3jabcMcaPiabc2faDbqaaiabdsgaKjabdEha3naaDaaaleaaiiGacqGFXoqyaeaacqWGRbWAaaaaaOGaeyypa0ZaaSaaaeaacqGHPms4cqWGUbGBdaqhaaWcbaGamaiJ+f7aHbqaaiabdUgaRbaakiabgQYiXdqaaiabdEha3naaDaaaleaacqGFXoqyaeaacqWGRbWAaaaaaOGaeiilaWcaaa@5D63@

where <nαk
 MathType@MTEF@5@5@+=feaafiart1ev1aaatCvAUfKttLearuWrP9MDH5MBPbIqV92AaeXatLxBI9gBaebbnrfifHhDYfgasaacH8akY=wiFfYdH8Gipec8Eeeu0xXdbba9frFj0=OqFfea0dXdd9vqai=hGuQ8kuc9pgc9s8qqaq=dirpe0xb9q8qiLsFr0=vr0=vr0dc8meaabaqaciaacaGaaeqabaqabeGadaaakeaacqWGUbGBdaqhaaWcbaacciGae8xSdegabaGaem4AaSgaaaaa@3143@> is the number of times letter *α *is expected to occur at position *k *of the regulatory sites under posterior distribution *P*(*i*|*D*,*w*).

To derive the last equality, first note that derivative is a sum of independent terms

dlog⁡[P(D|w)]dwαk=∑m=1ndlog⁡[P(Dm|w)]dwαk,
 MathType@MTEF@5@5@+=feaafiart1ev1aaatCvAUfKttLearuWrP9MDH5MBPbIqV92AaeXatLxBI9gBaebbnrfifHhDYfgasaacH8akY=wiFfYdH8Gipec8Eeeu0xXdbba9frFj0=OqFfea0dXdd9vqai=hGuQ8kuc9pgc9s8qqaq=dirpe0xb9q8qiLsFr0=vr0=vr0dc8meaabaqaciaacaGaaeqabaqabeGadaaakeaadaWcaaqaaiabdsgaKjGbcYgaSjabc+gaVjabcEgaNjabcUfaBjabdcfaqjabcIcaOiabdseaejabcYha8jabdEha3jabcMcaPiabc2faDbqaaiabdsgaKjabdEha3naaDaaaleaaiiGacqWFXoqyaeaacqWGRbWAaaaaaOGaeyypa0ZaaabCaeaadaWcaaqaaiabdsgaKjGbcYgaSjabc+gaVjabcEgaNjabcUfaBjabdcfaqjabcIcaOiabdseaenaaBaaaleaacqWGTbqBaeqaaOGaeiiFaWNaem4DaCNaeiykaKIaeiyxa0fabaGaemizaqMaem4DaC3aa0baaSqaaiab=f7aHbqaaiabdUgaRbaaaaaabaGaemyBa0Maeyypa0JaeGymaedabaGaemOBa4ganiabggHiLdGccqGGSaalaaa@60E0@

and that each term is again a sum of independent terms

dlog⁡[P(Dm|w)]dwαk=(L−l+1)−1P(Dm|w)∑imdP(Dm|w,im)dwαk.
 MathType@MTEF@5@5@+=feaafiart1ev1aaatCvAUfKttLearuWrP9MDH5MBPbIqV92AaeXatLxBI9gBaebbnrfifHhDYfgasaacH8akY=wiFfYdH8Gipec8Eeeu0xXdbba9frFj0=OqFfea0dXdd9vqai=hGuQ8kuc9pgc9s8qqaq=dirpe0xb9q8qiLsFr0=vr0=vr0dc8meaabaqaciaacaGaaeqabaqabeGadaaakeaadaWcaaqaaiabdsgaKjGbcYgaSjabc+gaVjabcEgaNjabcUfaBjabdcfaqjabcIcaOiabdseaenaaBaaaleaacqWGTbqBaeqaaOGaeiiFaWNaem4DaCNaeiykaKIaeiyxa0fabaGaemizaqMaem4DaC3aa0baaSqaaGGaciab=f7aHbqaaiabdUgaRbaaaaGccqGH9aqpdaWcaaqaaiabcIcaOiabdYeamjabgkHiTiabdYgaSjabgUcaRiabigdaXiabcMcaPmaaCaaaleqabaGaeyOeI0IaeGymaedaaaGcbaGaemiuaaLaeiikaGIaemiraq0aaSbaaSqaaiabd2gaTbqabaGccqGG8baFcqWG3bWDcqGGPaqkaaWaaabuaeaadaWcaaqaaiabdsgaKjabdcfaqjabcIcaOiabdseaenaaBaaaleaacqWGTbqBaeqaaOGaeiiFaWNaem4DaCNaeiilaWIaemyAaK2aaSbaaSqaaiabd2gaTbqabaGccqGGPaqkaeaacqWGKbazcqWG3bWDdaqhaaWcbaGae8xSdegabaGaem4AaSgaaaaaaeaacqWGPbqAdaWgaaadbaGaemyBa0gabeaaaSqab0GaeyyeIuoakiabc6caUaaa@6F5F@

Now if the base *s*(*i*_*m *_+ *k*) at position *i*_*m *_+ *k *of sequence *m *is equal to *α*, then the last derivative on the right simply divides *P*(*D*_*m*_|*w*, *i*_*m*_) by wαk
 MathType@MTEF@5@5@+=feaafiart1ev1aaatCvAUfKttLearuWrP9MDH5MBPbIqV92AaeXatLxBI9gBaebbnrfifHhDYfgasaacH8akY=wiFfYdH8Gipec8Eeeu0xXdbba9frFj0=OqFfea0dXdd9vqai=hGuQ8kuc9pgc9s8qqaq=dirpe0xb9q8qiLsFr0=vr0=vr0dc8meaabaqaciaacaGaaeqabaqabeGadaaakeaacqWG3bWDdaqhaaWcbaacciGae8xSdegabaGaem4AaSgaaaaa@3155@, and else the derivative is zero. We thus have

dP(Dm|w,im)dwαk=δ(s(im+k),α)wαkP(Dm|w,im),
 MathType@MTEF@5@5@+=feaafiart1ev1aaatCvAUfKttLearuWrP9MDH5MBPbIqV92AaeXatLxBI9gBaebbnrfifHhDYfgasaacH8akY=wiFfYdH8Gipec8Eeeu0xXdbba9frFj0=OqFfea0dXdd9vqai=hGuQ8kuc9pgc9s8qqaq=dirpe0xb9q8qiLsFr0=vr0=vr0dc8meaabaqaciaacaGaaeqabaqabeGadaaakeaadaWcaaqaaiabdsgaKjabdcfaqjabcIcaOiabdseaenaaBaaaleaacqWGTbqBaeqaaOGaeiiFaWNaem4DaCNaeiilaWIaemyAaK2aaSbaaSqaaiabd2gaTbqabaGccqGGPaqkaeaacqWGKbazcqWG3bWDdaqhaaWcbaacciGae8xSdegabaGaem4AaSgaaaaakiabg2da9maalaaabaGae8hTdqMaeiikaGIaem4CamNaeiikaGIaemyAaK2aaSbaaSqaaiabd2gaTbqabaGccqGHRaWkcqWGRbWAcqGGPaqkcqGGSaalcqWFXoqycqGGPaqkaeaacqWG3bWDdaqhaaWcbaGae8xSdegabaGaem4AaSgaaaaakiabdcfaqjabcIcaOiabdseaenaaBaaaleaacqWGTbqBaeqaaOGaeiiFaWNaem4DaCNaeiilaWIaemyAaK2aaSbaaSqaaiabd2gaTbqabaGccqGGPaqkcqGGSaalaaa@6170@

where the delta-function is one if *s*(*i*_*m *_+ *k*) = *α *and zero otherwise. We thus find

dlog⁡[P(Dm|w)]dwαk=∑imδ(s(im+k),α)P(im|D,w)wαk.
 MathType@MTEF@5@5@+=feaafiart1ev1aaatCvAUfKttLearuWrP9MDH5MBPbIqV92AaeXatLxBI9gBaebbnrfifHhDYfgasaacH8akY=wiFfYdH8Gipec8Eeeu0xXdbba9frFj0=OqFfea0dXdd9vqai=hGuQ8kuc9pgc9s8qqaq=dirpe0xb9q8qiLsFr0=vr0=vr0dc8meaabaqaciaacaGaaeqabaqabeGadaaakeaadaWcaaqaaiabdsgaKjGbcYgaSjabc+gaVjabcEgaNjabcUfaBjabdcfaqjabcIcaOiabdseaenaaBaaaleaacqWGTbqBaeqaaOGaeiiFaWNaem4DaCNaeiykaKIaeiyxa0fabaGaemizaqMaem4DaC3aa0baaSqaaGGaciab=f7aHbqaaiabdUgaRbaaaaGccqGH9aqpdaWcaaqaamaaqababaGae8hTdqMaeiikaGIaem4CamNaeiikaGIaemyAaK2aaSbaaSqaaiabd2gaTbqabaGccqGHRaWkcqWGRbWAcqGGPaqkcqGGSaalcqWFXoqycqGGPaqkaSqaaiabdMgaPnaaBaaameaacqWGTbqBaeqaaaWcbeqdcqGHris5aOGaemiuaaLaeiikaGIaemyAaK2aaSbaaSqaaiabd2gaTbqabaGccqGG8baFcqWGebarcqGGSaalcqWG3bWDcqGGPaqkaeaacqWG3bWDdaqhaaWcbaGae8xSdegabaGaem4AaSgaaaaakiabc6caUaaa@6788@

Note that the numerator of the right-hand side of this equation is just the expected number of times letter *α *occurs at position *k *of the binding sites in *D*_*m *_under the posterior distribution *P*(*i*_*m*_|*D,w*). Summing over all sequences *m *we thus obtain

dlog⁡[P(D|w)]dwαk=〈nαk〉wαk.
 MathType@MTEF@5@5@+=feaafiart1ev1aaatCvAUfKttLearuWrP9MDH5MBPbIqV92AaeXatLxBI9gBaebbnrfifHhDYfgasaacH8akY=wiFfYdH8Gipec8Eeeu0xXdbba9frFj0=OqFfea0dXdd9vqai=hGuQ8kuc9pgc9s8qqaq=dirpe0xb9q8qiLsFr0=vr0=vr0dc8meaabaqaciaacaGaaeqabaqabeGadaaakeaadaWcaaqaaiabdsgaKjGbcYgaSjabc+gaVjabcEgaNjabcUfaBjabdcfaqjabcIcaOiabdseaejabcYha8jabdEha3jabcMcaPiabc2faDbqaaiabdsgaKjabdEha3naaDaaaleaaiiGacqWFXoqyaeaacqWGRbWAaaaaaOGaeyypa0ZaaSaaaeaacqGHPms4cqWGUbGBdaqhaaWcbaGae8xSdegabaGaem4AaSgaaOGaeyOkJepabaGaem4DaC3aa0baaSqaaiab=f7aHbqaaiabdUgaRbaaaaGccqGGUaGlaaa@504B@

Using the fact that the WM columns are normalized, we find that at the maximum of *P*(*D*|*w*) the weight matrix components obey the equalities

wαk=〈nαk〉n
 MathType@MTEF@5@5@+=feaafiart1ev1aaatCvAUfKttLearuWrP9MDH5MBPbIqV92AaeXatLxBI9gBaebbnrfifHhDYfgasaacH8akY=wiFfYdH8Gipec8Eeeu0xXdbba9frFj0=OqFfea0dXdd9vqai=hGuQ8kuc9pgc9s8qqaq=dirpe0xb9q8qiLsFr0=vr0=vr0dc8meaabaqaciaacaGaaeqabaqabeGadaaakeaacqWG3bWDdaqhaaWcbaacciGae8xSdegabaGaem4AaSgaaOGaeyypa0ZaaSaaaeaacqGHPms4cqWGUbGBdaqhaaWcbaGae8xSdegabaGaem4AaSgaaOGaeyOkJepabaGaemOBa4gaaaaa@3BF2@

As in section "Finding clusters of binding sites: regulatory modules" one can use EM to solve these equations. We start with a randomly chosen WM *w *and calculate <nαk
 MathType@MTEF@5@5@+=feaafiart1ev1aaatCvAUfKttLearuWrP9MDH5MBPbIqV92AaeXatLxBI9gBaebbnrfifHhDYfgasaacH8akY=wiFfYdH8Gipec8Eeeu0xXdbba9frFj0=OqFfea0dXdd9vqai=hGuQ8kuc9pgc9s8qqaq=dirpe0xb9q8qiLsFr0=vr0=vr0dc8meaabaqaciaacaGaaeqabaqabeGadaaakeaacqWGUbGBdaqhaaWcbaacciGae8xSdegabaGaem4AaSgaaaaa@3143@> for that WM. We then update the WM components using equation (46) and repeat until the WM no longer changes. This procedure is guaranteed to converge to a local optimum of *P*(*D*|*w*).

Note that in the above we assumed just one site per sequence but it is easy to extend these derivations to arbitrary configurations, using the identities derived in section "Finding WM matches". Probably the first algorithm developed to find regulatory motifs in this way is the well-known MEME algorithm [17], and by now there are quite a number of algorithms that have been developed using this general idea, e.g. MDScan [18].

Once an optimal WM w_* _is found it is straightforward, i.e. using equation (19), to calculate the posterior probabilities *P*(*i*_*m*_|*D*, w_*_) that a site occurs at position *i*_*m *_in sequence *m *and this allows one to distinguish between high confidence and low confidence sites. Programs that use the EM approach to motif finding often report such probabilities. Note, however, that the posterior probabilities *P*(*i*_*m*_|*D*, w_*_) should not be confused with the posterior probabilities *P*(*i*_*m*_|*D*) which give the posterior probability that a site occurs at *i*_*m *_*independent *of what the WM *w *is (we derive an expression for this probability below). The latter quantifies how much evidence there is in the data *D *that a site occurs at *i*_*m*_, whereas *P*(*i*_*m*_|D,w_*_) assumes in addition that the inferred WM w_* _is correct. Since in many cases there is a reasonably high probability that w_* _does not match precisely the WM from which the site derives, the probabilities *P*(*i*_*m*_|D, w_*_) will typically be significantly larger than *P*(*i*_*m*_|*D*).

Finally, it would even be straightforward to extend the EM approach to multiple WMs using the expressions of section "Finding clusters of binding sites: regulatory modules". One could then, in principle, simultaneously find the set of priors {*π*_*w*_} and the set of WMs {*w*} that maximize the overall probability *P*(*D*|{*w*}, {*π*_*w*_}) of the data. For each *WM w *the expectation-maximization update equation of the WM components would take on the form

wαk=〈nαk(w)〉〈n(w)〉,
 MathType@MTEF@5@5@+=feaafiart1ev1aaatCvAUfKttLearuWrP9MDH5MBPbIqV92AaeXatLxBI9gBaebbnrfifHhDYfgasaacH8akY=wiFfYdH8Gipec8Eeeu0xXdbba9frFj0=OqFfea0dXdd9vqai=hGuQ8kuc9pgc9s8qqaq=dirpe0xb9q8qiLsFr0=vr0=vr0dc8meaabaqaciaacaGaaeqabaqabeGadaaakeaacqWG3bWDdaqhaaWcbaacciGae8xSdegabaGaem4AaSgaaOGaeyypa0ZaaSaaaeaacqGHPms4cqWGUbGBdaqhaaWcbaGae8xSdegabaGaem4AaSgaaOGaeiikaGIaem4DaCNaeiykaKIaeyOkJepabaGaeyykJeUaemOBa4MaeiikaGIaem4DaCNaeiykaKIaeyOkJepaaiabcYcaSaaa@46A7@

where <*n*(*w*)> is the expected total number of sites for WM *w *that occur in *D *and <nαk
 MathType@MTEF@5@5@+=feaafiart1ev1aaatCvAUfKttLearuWrP9MDH5MBPbIqV92AaeXatLxBI9gBaebbnrfifHhDYfgasaacH8akY=wiFfYdH8Gipec8Eeeu0xXdbba9frFj0=OqFfea0dXdd9vqai=hGuQ8kuc9pgc9s8qqaq=dirpe0xb9q8qiLsFr0=vr0=vr0dc8meaabaqaciaacaGaaeqabaqabeGadaaakeaacqWGUbGBdaqhaaWcbaacciGae8xSdegabaGaem4AaSgaaaaa@3143@(*w*)> is the expected number of those sites that have a base *α *at position *k*. The problem with this approach is that EM will very often lead to a local rather than the global optimum (it roughly speaking moves uphill from the starting point to the nearest local optimum). So depending on the initial sets {*w*} and {*π*_*w*_} the EM procedure may lead to very different optima and the higher the dimension of the search-space, the more serious this problem becomes. Therefore, in practice algorithms such as MEME do not search for multiple WMs simultaneously but rather find one WM at a time. In addition, programs like MEME will start from many different initial WMs *w *and perform EM for each of them, reporting the best optimum found in any of these EMs.

### Motif sampling

The second approach to motif finding focuses on the probability *P*(*D*|*i*). To calculate (37) we substitute (35) for the likelihood and first note that it can be separated in a part *P*(*B*_*i*_|*b*, *i*) that depends only on the background, and a part *P*(*S*_*i*_|*i*) that is given by an integral, i.e. *P*(*D*|*i*) = *P*(*B*_*i*_|*b,i*)*P*(*S*_*i*_|*i*) with

P(Bi|b,i)=∏σ∈Bibσ=∏α(bα)nα(Bi),
 MathType@MTEF@5@5@+=feaafiart1ev1aaatCvAUfKttLearuWrP9MDH5MBPbIqV92AaeXatLxBI9gBaebbnrfifHhDYfgasaacH8akY=wiFfYdH8Gipec8Eeeu0xXdbba9frFj0=OqFfea0dXdd9vqai=hGuQ8kuc9pgc9s8qqaq=dirpe0xb9q8qiLsFr0=vr0=vr0dc8meaabaqaciaacaGaaeqabaqabeGadaaakeaacqWGqbaucqGGOaakcqWGcbGqdaWgaaWcbaGaemyAaKgabeaakiabcYha8jabdkgaIjabcYcaSiabdMgaPjabcMcaPiabg2da9maarafabaGaemOyai2aaSbaaSqaaGGaciab=n8aZbqabaaabaGae83WdmNaeyicI4SaemOqai0aaSbaaWqaaiabdMgaPbqabaaaleqaniabg+GivdGccqGH9aqpdaqeqbqaaiabcIcaOiabdkgaInaaBaaaleaacqWFXoqyaeqaaOGaeiykaKYaaWbaaSqabeaacqWGUbGBdaWgaaadbaGae8xSdegabeaaliabcIcaOiabdkeacnaaBaaameaacqWGPbqAaeqaaSGaeiykaKcaaaqaaiab=f7aHbqab0Gaey4dIunakiabcYcaSaaa@557F@

where *n*_*α*_(*B*_*i*_) is the number of times base *α *occurs in the background *B*_*i*_, and

P(Si|i)=∫P(Si|w,i)P(w)dw=∫P(w)∏s∈SiP(s|w)dw.
 MathType@MTEF@5@5@+=feaafiart1ev1aaatCvAUfKttLearuWrP9MDH5MBPbIqV92AaeXatLxBI9gBaebbnrfifHhDYfgasaacH8akY=wiFfYdH8Gipec8Eeeu0xXdbba9frFj0=OqFfea0dXdd9vqai=hGuQ8kuc9pgc9s8qqaq=dirpe0xb9q8qiLsFr0=vr0=vr0dc8meaabaqaciaacaGaaeqabaqabeGadaaakeaacqWGqbaucqGGOaakcqWGtbWudaWgaaWcbaGaemyAaKgabeaakiabcYha8jabdMgaPjabcMcaPiabg2da9maapeaabaGaemiuaaLaeiikaGIaem4uam1aaSbaaSqaaiabdMgaPbqabaGccqGG8baFcqWG3bWDcqGGSaalcqWGPbqAcqGGPaqkaSqabeqaniabgUIiYdGccqWGqbaucqGGOaakcqWG3bWDcqGGPaqkcqWGKbazcqWG3bWDcqGH9aqpdaWdbaqaaiabdcfaqjabcIcaOiabdEha3jabcMcaPmaarafabaGaemiuaaLaeiikaGIaem4CamNaeiiFaWNaem4DaCNaeiykaKIaemizaqMaem4DaChaleaacqWGZbWCcqGHiiIZcqWGtbWudaWgaaadbaGaemyAaKgabeaaaSqab0Gaey4dIunaaSqabeqaniabgUIiYdGccqGGUaGlaaa@6425@

For the prior *P*(*w*) we use a Dirichlet prior as in (27), and use the general identity (31) to calculate the integral, which results in

P(Si|i)=∏k=1l[Γ(γk)Γ(n+γk)∏αΓ(nαk(Si)+γαk)Γ(γαk)],
 MathType@MTEF@5@5@+=feaafiart1ev1aaatCvAUfKttLearuWrP9MDH5MBPbIqV92AaeXatLxBI9gBaebbnrfifHhDYfgasaacH8akY=wiFfYdH8Gipec8Eeeu0xXdbba9frFj0=OqFfea0dXdd9vqai=hGuQ8kuc9pgc9s8qqaq=dirpe0xb9q8qiLsFr0=vr0=vr0dc8meaabaqaciaacaGaaeqabaqabeGadaaakeaacqWGqbaucqGGOaakcqWGtbWudaWgaaWcbaGaemyAaKgabeaakiabcYha8jabdMgaPjabcMcaPiabg2da9maarahabaWaamWaaeaadaWcaaqaaiabfo5ahjabcIcaOGGaciab=n7aNnaaCaaaleqabaGaem4AaSgaaOGaeiykaKcabaGaeu4KdCKaeiikaGIaemOBa4Maey4kaSIae83SdC2aaWbaaSqabeaacqWGRbWAaaGccqGGPaqkaaWaaebuaeaadaWcaaqaaiabfo5ahjabcIcaOiabd6gaUjaaxcW7daqhaaWcbaGae8xSdegabaGaem4AaSgaaOGaeiikaGIaem4uam1aaSbaaSqaaiabdMgaPbqabaGccqGGPaqkcqGHRaWkcqWFZoWzdaqhaaWcbaGae8xSdegabaGaem4AaSgaaOGaeiykaKcabaGaeu4KdCKaeiikaGIae83SdC2aa0baaSqaaiab=f7aHbqaaiabdUgaRbaakiabcMcaPaaaaSqaaiab=f7aHbqab0Gaey4dIunaaOGaay5waiaaw2faaaWcbaGaem4AaSMaeyypa0JaeGymaedabaGaemiBaWganiabg+GivdGccqGGSaalaaa@6E01@

where nαk
 MathType@MTEF@5@5@+=feaafiart1ev1aaatCvAUfKttLearuWrP9MDH5MBPbIqV92AaeXatLxBI9gBaebbnrfifHhDYfgasaacH8akY=wiFfYdH8Gipec8Eeeu0xXdbba9frFj0=OqFfea0dXdd9vqai=hGuQ8kuc9pgc9s8qqaq=dirpe0xb9q8qiLsFr0=vr0=vr0dc8meaabaqaciaacaGaaeqabaqabeGadaaakeaacqWGUbGBdaqhaaWcbaacciGae8xSdegabaGaem4AaSgaaaaa@3143@(*S*_*i*_) is the number of times base *α *occurs at position *k *of the sites in *S*_*i*_, and the γαk
 MathType@MTEF@5@5@+=feaafiart1ev1aaatCvAUfKttLearuWrP9MDH5MBPbIqV92AaeXatLxBI9gBaebbnrfifHhDYfgasaacH8akY=wiFfYdH8Gipec8Eeeu0xXdbba9frFj0=OqFfea0dXdd9vqai=hGuQ8kuc9pgc9s8qqaq=dirpe0xb9q8qiLsFr0=vr0=vr0dc8meaabaqaciaacaGaaeqabaqabeGadaaakeaaiiGacqWFZoWzdaqhaaWcbaGae8xSdegabaGaem4AaSgaaaaa@3180@ are again the pseudocounts of the Dirichlet prior. The most common situation is that we know little about the WM that can be expected and in such situations either a uniform prior γαk
 MathType@MTEF@5@5@+=feaafiart1ev1aaatCvAUfKttLearuWrP9MDH5MBPbIqV92AaeXatLxBI9gBaebbnrfifHhDYfgasaacH8akY=wiFfYdH8Gipec8Eeeu0xXdbba9frFj0=OqFfea0dXdd9vqai=hGuQ8kuc9pgc9s8qqaq=dirpe0xb9q8qiLsFr0=vr0=vr0dc8meaabaqaciaacaGaaeqabaqabeGadaaakeaaiiGacqWFZoWzdaqhaaWcbaGae8xSdegabaGaem4AaSgaaaaa@3180@ = 1 or one that biases toward the corners of the simplex, e.g. γαk
 MathType@MTEF@5@5@+=feaafiart1ev1aaatCvAUfKttLearuWrP9MDH5MBPbIqV92AaeXatLxBI9gBaebbnrfifHhDYfgasaacH8akY=wiFfYdH8Gipec8Eeeu0xXdbba9frFj0=OqFfea0dXdd9vqai=hGuQ8kuc9pgc9s8qqaq=dirpe0xb9q8qiLsFr0=vr0=vr0dc8meaabaqaciaacaGaaeqabaqabeGadaaakeaaiiGacqWFZoWzdaqhaaWcbaGae8xSdegabaGaem4AaSgaaaaa@3180@ = 0.5, are reasonable choices. However, as we mentioned in the discussion of equation (28), if we already have a set of known sites *S*_known _for the motif, in which base *α *appears mαk
 MathType@MTEF@5@5@+=feaafiart1ev1aaatCvAUfKttLearuWrP9MDH5MBPbIqV92AaeXatLxBI9gBaebbnrfifHhDYfgasaacH8akY=wiFfYdH8Gipec8Eeeu0xXdbba9frFj0=OqFfea0dXdd9vqai=hGuQ8kuc9pgc9s8qqaq=dirpe0xb9q8qiLsFr0=vr0=vr0dc8meaabaqaciaacaGaaeqabaqabeGadaaakeaacqWGTbqBdaqhaaWcbaacciGae8xSdegabaGaem4AaSgaaaaa@3141@ times at position *k*, then the posterior probability for the WM has the same form as a prior with counts γαk=mαk+1
 MathType@MTEF@5@5@+=feaafiart1ev1aaatCvAUfKttLearuWrP9MDH5MBPbIqV92AaeXatLxBI9gBaebbnrfifHhDYfgasaacH8akY=wiFfYdH8Gipec8Eeeu0xXdbba9frFj0=OqFfea0dXdd9vqai=hGuQ8kuc9pgc9s8qqaq=dirpe0xb9q8qiLsFr0=vr0=vr0dc8meaabaqaciaacaGaaeqabaqabeGadaaakeaaiiGacqWFZoWzdaqhaaWcbaGae8xSdegabaGaem4AaSgaaOGaeyypa0JaemyBa02aa0baaSqaaiab=f7aHbqaaiabdUgaRbaakiabgUcaRiabigdaXaaa@38F5@. Using this posterior as a prior in equation (50) we can thus also calculate the probability of obtaining the sequence segments in *S*_*i *_when sampling from the same WM as the WM from which the set *S*_known _derived. That is, equation (50) easily allows for the incorporation of prior knowledge about the WM *w*.

#### The meaning of equation (50)

Since the expression (50) is central in all motif sampling strategies we will divert here to discuss its meaning in a little more detail. First, note that *P*(*S*_*i*_|*i*) is a product of independent factors for each column *k*. We thus focus on a single column only. In addition, we will assume a uniform prior over WMs, i.e. γαk
 MathType@MTEF@5@5@+=feaafiart1ev1aaatCvAUfKttLearuWrP9MDH5MBPbIqV92AaeXatLxBI9gBaebbnrfifHhDYfgasaacH8akY=wiFfYdH8Gipec8Eeeu0xXdbba9frFj0=OqFfea0dXdd9vqai=hGuQ8kuc9pgc9s8qqaq=dirpe0xb9q8qiLsFr0=vr0=vr0dc8meaabaqaciaacaGaaeqabaqabeGadaaakeaaiiGacqWFZoWzdaqhaaWcbaGae8xSdegabaGaem4AaSgaaaaa@3180@ = 1. The expression for a single column then takes on the simpler form

P(S)=3!∏αnα!(n+3)!=3!n!(n+3)!∏αnα!n!,
MathType@MTEF@5@5@+=feaafiart1ev1aaatCvAUfKttLearuWrP9MDH5MBPbIqV92AaeXatLxBI9gBaebbnrfifHhDYfgasaacH8akY=wiFfYdH8Gipec8Eeeu0xXdbba9frFj0=OqFfea0dXdd9vqai=hGuQ8kuc9pgc9s8qqaq=dirpe0xb9q8qiLsFr0=vr0=vr0dc8meaabaqaciaacaGaaeqabaqabeGadaaakeaacqWGqbaucqGGOaakcqWGtbWucqGGPaqkcqGH9aqpdaWcaaqaaiabiodaZiabcgcaHmaarababaGaemOBa42aaSbaaSqaaGGaciab=f7aHbqabaGccqGGHaqiaSqaaiab=f7aHbqab0Gaey4dIunaaOqaaiabcIcaOiabd6gaUjabgUcaRiabiodaZiabcMcaPiabcgcaHaaacqGH9aqpdaWcaaqaaiabiodaZiabcgcaHiabd6gaUjabcgcaHaqaaiabcIcaOiabd6gaUjabgUcaRiabiodaZiabcMcaPiabcgcaHaaadaWcaaqaamaarababaGaemOBa42aaSbaaSqaaiab=f7aHbqabaGccqGGHaqiaSqaaiab=f7aHbqab0Gaey4dIunaaOqaaiabd6gaUjabcgcaHaaacqGGSaalaaa@5610@

where we used that Γ(*x *+ 1) = *x*! for integer *x*. The second equality on the right is to clarify that *P*(*S*) can be written as the product of two factors. The first of these factors, 3!*n*!/(*n *+ 3)!, is the inverse of the binomial coefficient (n+33)
 MathType@MTEF@5@5@+=feaafiart1ev1aaatCvAUfKttLearuWrP9MDH5MBPbIqV92AaeXatLxBI9gBaebbnrfifHhDYfgasaacH8akY=wiFfYdH8Gipec8Eeeu0xXdbba9frFj0=OqFfea0dXdd9vqai=hGuQ8kuc9pgc9s8qqaq=dirpe0xb9q8qiLsFr0=vr0=vr0dc8meaabaqaciaacaGaaeqabaqabeGadaaakeaadaqadaqaauaabeqaceaaaeaacqWGUbGBcqGHRaWkcqaIZaWmaeaacqaIZaWmaaaacaGLOaGaayzkaaaaaa@3271@. This binomial coefficient corresponds to the number of different sets of counts {*n*_*α*_} that are possible. That is, it counts the number of vectors of integers (*n*_*a*_, *n*_*c*_, *n*_*g*_, *n*_*t*_) such that ∑_*α *_*n*_*α *_= *n*.

The second factor in equation (51), ∏_*α *_*n*_*α*_!/*n*!, is the inverse of the multinomial coefficient *n*!/(∏_*α *_*n*_*α*_!) which gives the number of different ways that *n *objects can be distributed over 4 boxes such that *n*_*a *_objects are in the first box, *n*_*c *_in the second, *n*_*g *_in the third, and *n*_*t *_in the fourth. Thus, the probability *P*(*S*) for a column of *n *bases is inversely proportional to the number of ways in which the counts {*n*_*α*_} of this column can be realized. In summary, there are 4^*n *^possible outcomes for the *n *bases in the column. The probability distribution *P*(*S*) assigns a probability to each of these that is precisely inversely proportional to the number of the 4^*n *^outcomes that lead to the counts {*n*_*α*_}. As a result, the total probability to obtain an outcome with counts {*n*_*α*_} is *constant *for all (n+33)
 MathType@MTEF@5@5@+=feaafiart1ev1aaatCvAUfKttLearuWrP9MDH5MBPbIqV92AaeXatLxBI9gBaebbnrfifHhDYfgasaacH8akY=wiFfYdH8Gipec8Eeeu0xXdbba9frFj0=OqFfea0dXdd9vqai=hGuQ8kuc9pgc9s8qqaq=dirpe0xb9q8qiLsFr0=vr0=vr0dc8meaabaqaciaacaGaaeqabaqabeGadaaakeaadaqadaqaauaabeqaceaaaeaacqWGUbGBcqGHRaWkcqaIZaWmaeaacqaIZaWmaaaacaGLOaGaayzkaaaaaa@3271@ possible counts (because we have to sum *P*(*S*) over all possible outcomes that lead to the same set of counts).

For large *n *we can approximate the multinomial coefficient using Stirling's approximation to find

∏αnα!n!≈e−nH({nα}),
MathType@MTEF@5@5@+=feaafiart1ev1aaatCvAUfKttLearuWrP9MDH5MBPbIqV92AaeXatLxBI9gBaebbnrfifHhDYfgasaacH8akY=wiFfYdH8Gipec8Eeeu0xXdbba9frFj0=OqFfea0dXdd9vqai=hGuQ8kuc9pgc9s8qqaq=dirpe0xb9q8qiLsFr0=vr0=vr0dc8meaabaqaciaacaGaaeqabaqabeGadaaakeaadaWcaaqaamaarababaGaemOBa42aaSbaaSqaaGGaciab=f7aHbqabaGccqGGHaqiaSqaaiab=f7aHbqab0Gaey4dIunaaOqaaiabd6gaUjabcgcaHaaacqGHijYUcqWGLbqzdaahaaWcbeqaaiabgkHiTiabd6gaUjabdIeaijabcIcaOiabcUha7jabd6gaUnaaBaaameaacqWFXoqyaeqaaSGaeiyFa0NaeiykaKcaaOGaeiilaWcaaa@45DB@

where *H*({*n*_*α*_}) is the entropy of the distribution *n*_*α*_/*n*:

H({nα})=−∑αnαnlog⁡(nαn).
 MathType@MTEF@5@5@+=feaafiart1ev1aaatCvAUfKttLearuWrP9MDH5MBPbIqV92AaeXatLxBI9gBaebbnrfifHhDYfgasaacH8akY=wiFfYdH8Gipec8Eeeu0xXdbba9frFj0=OqFfea0dXdd9vqai=hGuQ8kuc9pgc9s8qqaq=dirpe0xb9q8qiLsFr0=vr0=vr0dc8meaabaqaciaacaGaaeqabaqabeGadaaakeaacqWGibascqGGOaakcqGG7bWEcqWGUbGBdaWgaaWcbaacciGae8xSdegabeaakiabc2ha9jabcMcaPiabg2da9iabgkHiTmaaqafabaWaaSaaaeaacqWGUbGBdaWgaaWcbaGae8xSdegabeaaaOqaaiabd6gaUbaaaSqaaiab=f7aHbqab0GaeyyeIuoakiGbcYgaSjabc+gaVjabcEgaNnaabmaabaWaaSaaaeaacqWGUbGBdaWgaaWcbaGae8xSdegabeaaaOqaaiabd6gaUbaaaiaawIcacaGLPaaacqGGUaGlaaa@4B51@

Thus, the probability *P*(*S*) is largest for sets of sequences whose base distributions have lowest entropy.

#### Back to motif sampling

We now return to our motif sampling calculations. Using (50) and (48) we obtain *P*(*D*|*i*) in terms of the counts nαk
 MathType@MTEF@5@5@+=feaafiart1ev1aaatCvAUfKttLearuWrP9MDH5MBPbIqV92AaeXatLxBI9gBaebbnrfifHhDYfgasaacH8akY=wiFfYdH8Gipec8Eeeu0xXdbba9frFj0=OqFfea0dXdd9vqai=hGuQ8kuc9pgc9s8qqaq=dirpe0xb9q8qiLsFr0=vr0=vr0dc8meaabaqaciaacaGaaeqabaqabeGadaaakeaacqWGUbGBdaqhaaWcbaacciGae8xSdegabaGaem4AaSgaaaaa@3143@(*S*_*i*_) and *n*_*α*_(*B*_*i*_). Finally, using a uniform prior over hypotheses *i*, the posterior *P*(*i|D*) becomes simply

P(i|D)=P(D|i)∑jP(D|j),
 MathType@MTEF@5@5@+=feaafiart1ev1aaatCvAUfKttLearuWrP9MDH5MBPbIqV92AaeXatLxBI9gBaebbnrfifHhDYfgasaacH8akY=wiFfYdH8Gipec8Eeeu0xXdbba9frFj0=OqFfea0dXdd9vqai=hGuQ8kuc9pgc9s8qqaq=dirpe0xb9q8qiLsFr0=vr0=vr0dc8meaabaqaciaacaGaaeqabaqabeGadaaakeaacqWGqbaucqGGOaakcqWGPbqAcqGG8baFcqWGebarcqGGPaqkcqGH9aqpdaWcaaqaaiabdcfaqjabcIcaOiabdseaejabcYha8jabdMgaPjabcMcaPaqaamaaqababaGaemiuaaLaeiikaGIaemiraqKaeiiFaWNaemOAaOMaeiykaKcaleaacqWGQbGAaeqaniabggHiLdaaaOGaeiilaWcaaa@4643@

where the sum in the denominator is over all possible assignments *j *= (*j*_1_,..., *j*_*n*_) for the positions of the binding sites.

Ideally we would now either find the configuration of site positions i_* _that maximizes *P*(*i*|*D*), or we would for each position *i*_*k *_calculate the posterior probability *P*(*i*_*k*_|*D*) that a site occurs at position *i*_*k *_in sequence *k*, which is formally given by

P(ik|D)=∑i1,...,ik−1,ik+1,...,inP(i|D).
 MathType@MTEF@5@5@+=feaafiart1ev1aaatCvAUfKttLearuWrP9MDH5MBPbIqV92AaeXatLxBI9gBaebbnrfifHhDYfgasaacH8akY=wiFfYdH8Gipec8Eeeu0xXdbba9frFj0=OqFfea0dXdd9vqai=hGuQ8kuc9pgc9s8qqaq=dirpe0xb9q8qiLsFr0=vr0=vr0dc8meaabaqaciaacaGaaeqabaqabeGadaaakeaacqWGqbaucqGGOaakcqWGPbqAdaWgaaWcbaGaem4AaSgabeaakiabcYha8jabdseaejabcMcaPiabg2da9maaqafabaGaemiuaaLaeiikaGIaemyAaKMaeiiFaWNaemiraqKaeiykaKcaleaacqWGPbqAdaWgaaadbaGaeGymaedabeaaliabcYcaSiabc6caUiabc6caUiabc6caUiabcYcaSiabdMgaPnaaBaaameaacqWGRbWAcqGHsislcqaIXaqmaeqaaSGaeiilaWIaemyAaK2aaSbaaWqaaiabdUgaRjabgUcaRiabigdaXaqabaWccqGGSaalcqGGUaGlcqGGUaGlcqGGUaGlcqGGSaalcqWGPbqAdaWgaaadbaGaemOBa4gabeaaaSqab0GaeyyeIuoakiabc6caUaaa@58AC@

**Figure 4:**
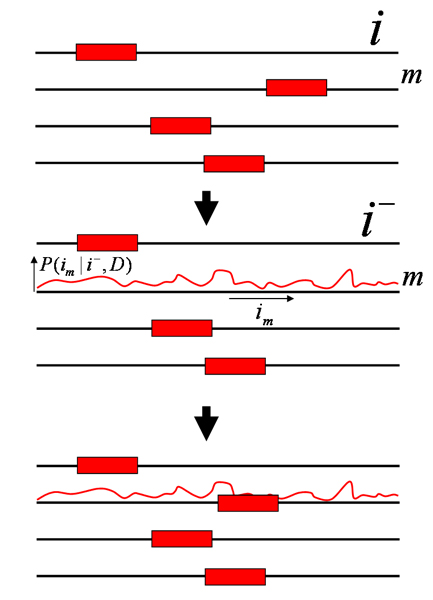
**Illustration of the steps of the Gibbs sampling algorithm. **The red profile indicates the posterior probability *P*(*i*_*m*_|*D*, *i*^-^) and in the last step a new position is sampled from this distribution.

Unfortunately, since *P*(*i*|*D*) is a complicated nonlinear function of the base counts nαk
 MathType@MTEF@5@5@+=feaafiart1ev1aaatCvAUfKttLearuWrP9MDH5MBPbIqV92AaeXatLxBI9gBaebbnrfifHhDYfgasaacH8akY=wiFfYdH8Gipec8Eeeu0xXdbba9frFj0=OqFfea0dXdd9vqai=hGuQ8kuc9pgc9s8qqaq=dirpe0xb9q8qiLsFr0=vr0=vr0dc8meaabaqaciaacaGaaeqabaqabeGadaaakeaacqWGUbGBdaqhaaWcbaacciGae8xSdegabaGaem4AaSgaaaaa@3143@(*S*_*i*_) and *n*_*α*_(*B*_*i*_) we cannot separate it easily into contributions from the different hypothesized sites in *i *and there is generally no way to calculate sums like (55) other than explicitly summing over all (*L *- *l *+ 1)^*n*-1 ^states. To find site configurations *i *with high *P*(*i*|*D*) researchers have in general resorted to Markov chain Monte-Carlo techniques for sampling the distribution *P*(*i*|*S*)[19]. The most commonly used way of sampling the distribution *P*(*i*|*S*) is through so-called *Gibbs sampling *[20] and consists of iterations of the following steps, which are illustrated in Fig. 4

1. Randomly select one of the *n *sequences with uniform probability.

2. If sequence number *m *was selected, remove the segment *s *located at position *i*_*m *_from the set of sites *S*_*i *_of the current configuration. Denote this set of (*n *- 1) sequences as Si−
 MathType@MTEF@5@5@+=feaafiart1ev1aaatCvAUfKttLearuWrP9MDH5MBPbIqV92AaeXatLxBI9gBaebbnrfifHhDYfgasaacH8akY=wiFfYdH8Gipec8Eeeu0xXdbba9frFj0=OqFfea0dXdd9vqai=hGuQ8kuc9pgc9s8qqaq=dirpe0xb9q8qiLsFr0=vr0=vr0dc8meaabaqaciaacaGaaeqabaqabeGadaaakeaacqWGtbWudaqhaaWcbaGaemyAaKgabaGaeyOeI0caaaaa@3050@ and the new configuration as *i*^-^.

3. For every position *i*_*m *_= 0 through *i*_*m *_= *L *- *l *denote the new configuration that results from placing the site at *i*_*m *_in sequence *m *as (*i*^-^, *i*_*m*_) and calculate *P*(*D*|*i*^-^, *i*_*m*_).

4. Select a new configuration by sampling the position of the site in sequence *m *according to the probability distribution

P(im|D,i−)=P(D|i−,im)∑jm=0L−lP(D|i−,jm).
 MathType@MTEF@5@5@+=feaafiart1ev1aaatCvAUfKttLearuWrP9MDH5MBPbIqV92AaeXatLxBI9gBaebbnrfifHhDYfgasaacH8akY=wiFfYdH8Gipec8Eeeu0xXdbba9frFj0=OqFfea0dXdd9vqai=hGuQ8kuc9pgc9s8qqaq=dirpe0xb9q8qiLsFr0=vr0=vr0dc8meaabaqaciaacaGaaeqabaqabeGadaaakeaacqWGqbaucqGGOaakcqWGPbqAdaWgaaWcbaGaemyBa0gabeaakiabcYha8jabdseaejabcYcaSiabdMgaPnaaCaaaleqabaGaeyOeI0caaOGaeiykaKIaeyypa0ZaaSaaaeaacqWGqbaucqGGOaakcqWGebarcqGG8baFcqWGPbqAdaahaaWcbeqaaiabgkHiTaaakiabcYcaSiabdMgaPnaaBaaaleaacqWGTbqBaeqaaOGaeiykaKcabaWaaabmaeaacqWGqbaucqGGOaakcqWGebarcqGG8baFcqWGPbqAdaahaaWcbeqaaiabgkHiTaaakiabcYcaSiabdQgaQnaaBaaaleaacqWGTbqBaeqaaOGaeiykaKcaleaacqWGQbGAdaWgaaadbaGaemyBa0gabeaaliabg2da9iabicdaWaqaaiabdYeamjabgkHiTiabdYgaSbqdcqGHris5aaaakiabc6caUaaa@5C4C@

using (48) and (50) one finds that this probability is proportional to

P(im|D,i−)∝∏k=1lns(im+k)k(Si−)+γikbs(im+k)(n−1+γk),
 MathType@MTEF@5@5@+=feaafiart1ev1aaatCvAUfKttLearuWrP9MDH5MBPbIqV92AaeXatLxBI9gBaebbnrfifHhDYfgasaacH8akY=wiFfYdH8Gipec8Eeeu0xXdbba9frFj0=OqFfea0dXdd9vqai=hGuQ8kuc9pgc9s8qqaq=dirpe0xb9q8qiLsFr0=vr0=vr0dc8meaabaqaciaacaGaaeqabaqabeGadaaakeaacqWGqbaucqGGOaakcqWGPbqAdaWgaaWcbaGaemyBa0gabeaakiabcYha8jabdseaejabcYcaSiabdMgaPnaaCaaaleqabaGaeyOeI0caaOGaeiykaKIaeyyhIu7aaebCaeaadaWcaaqaaiabd6gaUnaaDaaaleaacqWGZbWCcqGGOaakcqWGPbqAdaWgaaadbaGaemyBa0gabeaaliabgUcaRiabdUgaRjabcMcaPaqaaiabdUgaRbaakiabcIcaOiabdofatnaaDaaaleaacqWGPbqAaeaacqGHsislaaGccqGGPaqkcqGHRaWkiiGacqWFZoWzdaqhaaWcbaGaemyAaKgabaGaem4AaSgaaaGcbaGaemOyai2aaSbaaSqaaiabdohaZjabcIcaOiabdMgaPnaaBaaameaacqWGTbqBaeqaaSGaey4kaSIaem4AaSMaeiykaKcabeaakiabcIcaOiabd6gaUjabgkHiTiabigdaXiabgUcaRiab=n7aNnaaCaaaleqabaGaem4AaSgaaOGaeiykaKcaaaWcbaGaem4AaSMaeyypa0JaeGymaedabaGaemiBaWganiabg+GivdGccqGGSaalaaa@6AE9@

where *s*(*i*_*m *_+ *k*) is the base that occurs at position *i*_*m *_+ *k *in sequence *m*. Note that this expression is precisely the ratio between the probability *P*(*S*[*i*m,L]|Si) of the site at *i*_*m *_deriving from the same WM as the others in Si−
 MathType@MTEF@5@5@+=feaafiart1ev1aaatCvAUfKttLearuWrP9MDH5MBPbIqV92AaeXatLxBI9gBaebbnrfifHhDYfgasaacH8akY=wiFfYdH8Gipec8Eeeu0xXdbba9frFj0=OqFfea0dXdd9vqai=hGuQ8kuc9pgc9s8qqaq=dirpe0xb9q8qiLsFr0=vr0=vr0dc8meaabaqaciaacaGaaeqabaqabeGadaaakeaacqWGtbWudaqhaaWcbaGaemyAaKgabaGaeyOeI0caaaaa@3050@, i.e. as in equation (34), and the probability P(s[im,l]|b)
 MathType@MTEF@5@5@+=feaafiart1ev1aaatCvAUfKttLearuWrP9MDH5MBPbIqV92AaeXatLxBI9gBaebbnrfifHhDYfgasaacH8akY=wiFfYdH8Gipec8Eeeu0xXdbba9frFj0=OqFfea0dXdd9vqai=hGuQ8kuc9pgc9s8qqaq=dirpe0xb9q8qiLsFr0=vr0=vr0dc8meaabaqaciaacaGaaeqabaqabeGadaaakeaacqWGqbaucqGGOaakcqWGZbWCdaWgaaWcbaGaei4waSLaemyAaK2aaSbaaWqaaiabd2gaTbqabaWccqGGSaalcqWGSbaBcqGGDbqxaeqaaOGaeiiFaWNaemOyaiMaeiykaKcaaa@3BB0@ of this segment under the background, i.e.

P(im|D,i−)∝P(s[im,l]|Si−)P(s[im,l]|b).
 MathType@MTEF@5@5@+=feaafiart1ev1aaatCvAUfKttLearuWrP9MDH5MBPbIqV92AaeXatLxBI9gBaebbnrfifHhDYfgasaacH8akY=wiFfYdH8Gipec8Eeeu0xXdbba9frFj0=OqFfea0dXdd9vqai=hGuQ8kuc9pgc9s8qqaq=dirpe0xb9q8qiLsFr0=vr0=vr0dc8meaabaqaciaacaGaaeqabaqabeGadaaakeaacqWGqbaucqGGOaakcqWGPbqAdaWgaaWcbaGaemyBa0gabeaakiabcYha8jabdseaejabcYcaSiabdMgaPnaaCaaaleqabaGaeyOeI0caaOGaeiykaKIaeyyhIu7aaSaaaeaacqWGqbaucqGGOaakcqWGZbWCdaWgaaWcbaGaei4waSLaemyAaK2aaSbaaWqaaiabd2gaTbqabaWccqGGSaalcqWGSbaBcqGGDbqxaeqaaOGaeiiFaWNaem4uam1aa0baaSqaaiabdMgaPbqaaiabgkHiTaaakiabcMcaPaqaaiabdcfaqjabcIcaOiabdohaZnaaBaaaleaacqGGBbWwcqWGPbqAdaWgaaadbaGaemyBa0gabeaaliabcYcaSiabdYgaSjabc2faDbqabaGccqGG8baFcqWGIbGycqGGPaqkaaGaeiOla4caaa@5B48@

By iterating these steps one can sample the entire distribution *P*(*i*|*D*) and, for example, estimate the posterior probability *P*(*i*_*m*_|*D*) that a site occurs at position *i*_*m *_in sequence *m*, i.e. by the fraction of time a site occurs at *i*_*m *_during sampling. The probabilities *P*(*i*_*m*_|*D*) rigorously quantify the evidence in *D *that a site occurs at position *i*_*m*_. Thus, whenever *P*(*i*_*m*_|*D*) is large we can be confident that a site does occur at *i*_*m*_.

To make a single prediction for the set of regulatory sites in *D *one searches for the configuration i_* _that maximizes *P*(*i*|*D*). In some approaches, e.g. [21], this is done simply by keeping track of the highest probability configuration that was observed during sampling. However, more accurate determination of the optimal configuration *i *can be obtained through *simulated annealing *[22]. One introduces a parameter *β *and instead of sampling from *P*(*i*|*D*) one samples from a probability distribution which is proportional to *P*(*i*|*D*)^*β*^. At the start of the search *β *is set to a small number and then *β *is slowly increased with time. As *β *increases more weight will be put on configurations with high probability and eventually the sampler will 'freeze' into a state with locally optimal probability *P*(*i*|*D*). Provided the annealing is done slowly enough the optimum will correspond to the globally optimal state. This is for example the approach taken by the PhyloGibbs algorithm [23].

Once an optimal state i_* _is found through simulated annealing one can of course use normal sampling, i.e. with *β *= 1, to obtain the posterior probabilities of the sites in i_*_. Given the optimal configuration i_* _one can of course also report the expected WM given this configuration, which has components

〈wαk〉=nαk(Si*)+γαkn+γk.
 MathType@MTEF@5@5@+=feaafiart1ev1aaatCvAUfKttLearuWrP9MDH5MBPbIqV92AaeXatLxBI9gBaebbnrfifHhDYfgasaacH8akY=wiFfYdH8Gipec8Eeeu0xXdbba9frFj0=OqFfea0dXdd9vqai=hGuQ8kuc9pgc9s8qqaq=dirpe0xb9q8qiLsFr0=vr0=vr0dc8meaabaqaciaacaGaaeqabaqabeGadaaakeaacqGHPms4cqWG3bWDdaqhaaWcbaacciGae8xSdegabaGaem4AaSgaaOGaeyOkJeVaeyypa0ZaaSaaaeaacqWGUbGBdaqhaaWcbaGae8xSdegabaGaem4AaSgaaOGaeiikaGIaem4uam1aaSbaaSqaaiabdMgaPnaaBaaameaacqGGQaGkaeqaaaWcbeaakiabcMcaPiabgUcaRiab=n7aNnaaDaaaleaacqWFXoqyaeaacqWGRbWAaaaakeaacqWGUbGBcqGHRaWkcqWFZoWzdaahaaWcbeqaaiabdUgaRbaaaaGccqGGUaGlaaa@4C2A@

**Figure 5:**
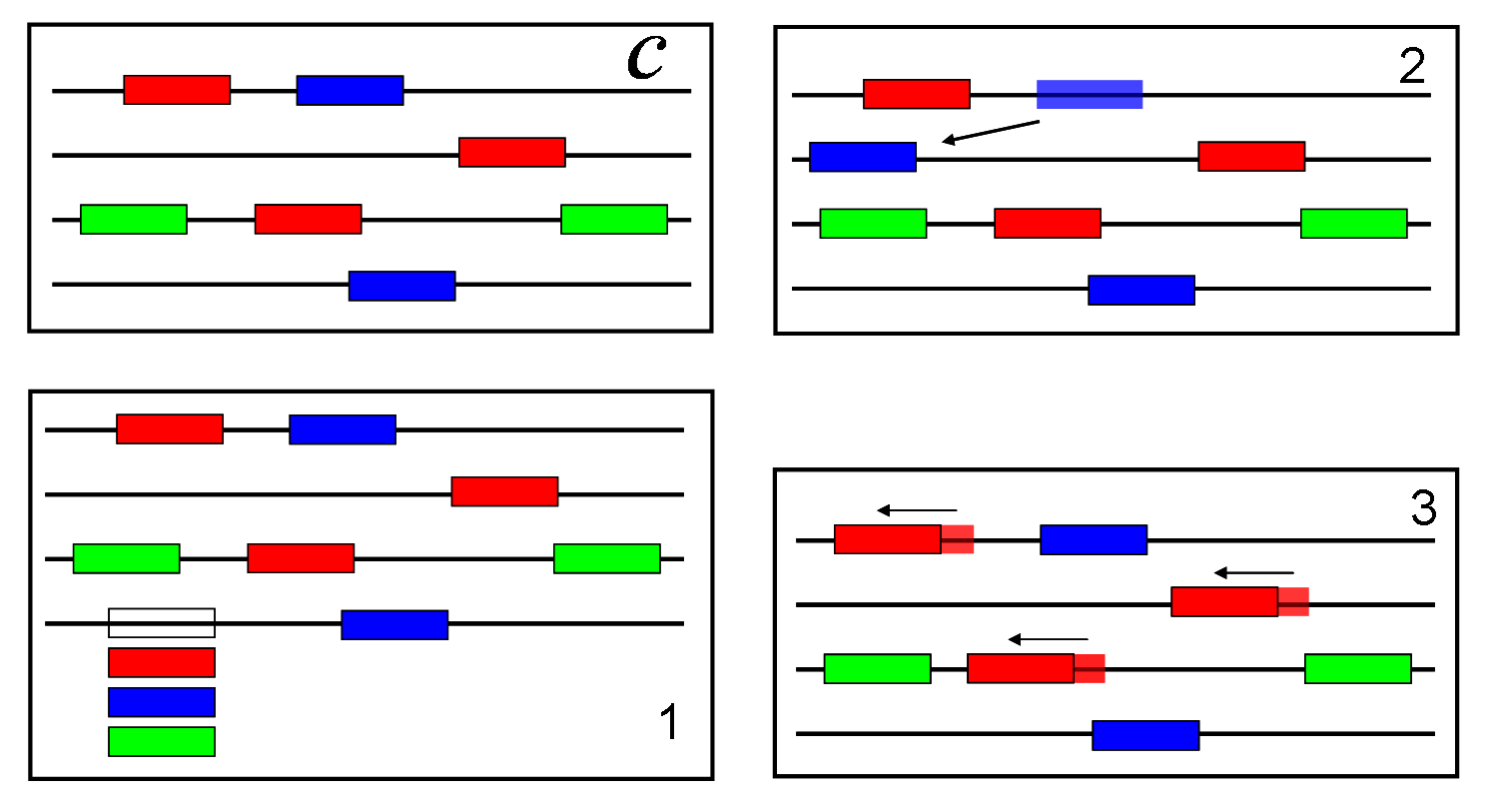
**Illustration of a general configuration with varying site numbers for multiple motifs (upper left) and examples of moves used to sample all possible configurations.** In 1 a randomly chosen segment is 'recolored', leaving it either blank (background), coloring with any of the existing motifs, or coloring it with a new color (new motif). In 2 a colored segment is chosen at random and moved to another location. In 3 all segments in a motif are shifted by the same amount.

Instead of assuming that there is precisely one site in each of the *n *sequences we can of course also sample much more general configurations *c*. Most generally, one could allow varying numbers of sites for multiple WMs. The top left panel in figure 5 shows such a general configuration with sites for 3 different motifs (red, blue, and green). If we assume the same kind of priors as we used in section "Finding clusters of binding sites: regulatory modules" then the prior probability for a particular configuration *c*, which has *n*(*w*, *c*) sites for WM *w *and *n*(*b, c*) bases in background, is proportional to

P(c|{π})∝[(πbg)n(b,c)]∏w(πw)n(w,c).
 MathType@MTEF@5@5@+=feaafiart1ev1aaatCvAUfKttLearuWrP9MDH5MBPbIqV92AaeXatLxBI9gBaebbnrfifHhDYfgasaacH8akY=wiFfYdH8Gipec8Eeeu0xXdbba9frFj0=OqFfea0dXdd9vqai=hGuQ8kuc9pgc9s8qqaq=dirpe0xb9q8qiLsFr0=vr0=vr0dc8meaabaqaciaacaGaaeqabaqabeGadaaakeaacqWGqbaucqGGOaakcqWGJbWycqGG8baFcqGG7bWEiiGacqWFapaCcqGG9bqFcqGGPaqkcqGHDisTdaWadaqaaiabcIcaOiab=b8aWnaaBaaaleaaieaacqGFIbGycqGFNbWzaeqaaOGaeiykaKYaaWbaaSqabeaacqWGUbGBcqGGOaakcqWGIbGycqGGSaalcqWGJbWycqGGPaqkaaaakiaawUfacaGLDbaadaqeqbqaaiabcIcaOiab=b8aWnaaBaaaleaacqWG3bWDaeqaaOGaeiykaKYaaWbaaSqabeaacqWGUbGBcqGGOaakcqWG3bWDcqGGSaalcqWGJbWycqGGPaqkaaaabaGaem4DaChabeqdcqGHpis1aOGaeiOla4caaa@5806@

If we denote the set of sites for WM *w *in configuration *c *by *S*_*w *_and the set of background nucleotides as *B*(*c*) we obtain for the likelihood of the data given the configuration

P(D|c)=[∏σ∈B(c)bσ]∏wP(Sw),
 MathType@MTEF@5@5@+=feaafiart1ev1aaatCvAUfKttLearuWrP9MDH5MBPbIqV92AaeXatLxBI9gBaebbnrfifHhDYfgasaacH8akY=wiFfYdH8Gipec8Eeeu0xXdbba9frFj0=OqFfea0dXdd9vqai=hGuQ8kuc9pgc9s8qqaq=dirpe0xb9q8qiLsFr0=vr0=vr0dc8meaabaqaciaacaGaaeqabaqabeGadaaakeaacqWGqbaucqGGOaakcqWGebarcqGG8baFcqWGJbWycqGGPaqkcqGH9aqpdaWadaqaamaarafabaGaemOyai2aaSbaaSqaaGGaciab=n8aZbqabaaabaGae83WdmNaeyicI4SaemOqaiKaeiikaGIaem4yamMaeiykaKcabeqdcqGHpis1aaGccaGLBbGaayzxaaWaaebuaeaacqWGqbaucqGGOaakcqWGtbWudaWgaaWcbaGaem4DaChabeaakiabcMcaPaWcbaGaem4DaChabeqdcqGHpis1aOGaeiilaWcaaa@4D2D@

where for each group of sites the probability *P*(*S*_*w*_) is given in complete analogy with (50) by

P(Sw)=∏k=1l[Γ(γk)Γ(n(w)+γk)∏αΓ(nαk(sw)+γαk)Γ(γαk)],
 MathType@MTEF@5@5@+=feaafiart1ev1aaatCvAUfKttLearuWrP9MDH5MBPbIqV92AaeXatLxBI9gBaebbnrfifHhDYfgasaacH8akY=wiFfYdH8Gipec8Eeeu0xXdbba9frFj0=OqFfea0dXdd9vqai=hGuQ8kuc9pgc9s8qqaq=dirpe0xb9q8qiLsFr0=vr0=vr0dc8meaabaqaciaacaGaaeqabaqabeGadaaakeaacqWGqbaucqGGOaakcqWGtbWudaWgaaWcbaGaem4DaChabeaakiabcMcaPiabg2da9maarahabaWaamWaaeaadaWcaaqaaiabfo5ahjabcIcaOGGaciab=n7aNnaaCaaaleqabaGaem4AaSgaaOGaeiykaKcabaGaeu4KdCKaeiikaGIaemOBa4MaeiikaGIaem4DaCNaeiykaKIaey4kaSIae83SdC2aaWbaaSqabeaacqWGRbWAaaGccqGGPaqkaaWaaebuaeaadaWcaaqaaiabfo5ahjabcIcaOiabd6gaUnaaDaaaleaacqWFXoqyaeaacqWGRbWAaaGccqGGOaakcqWGZbWCdaWgaaWcbaGaem4DaChabeaakiabcMcaPiabgUcaRiab=n7aNnaaDaaaleaacqWFXoqyaeaacqWGRbWAaaGccqGGPaqkaeaacqqHtoWrcqGGOaakcqWFZoWzdaqhaaWcbaGae8xSdegabaGaem4AaSgaaOGaeiykaKcaaaWcbaGae8xSdegabeqdcqGHpis1aaGccaGLBbGaayzxaaaaleaacqWGRbWAcqGH9aqpcqaIXaqmaeaacqWGSbaBa0Gaey4dIunakiabcYcaSaaa@6D3F@

where *n*(*w*) is the total number of sites in group *S*_*w *_and nαk
 MathType@MTEF@5@5@+=feaafiart1ev1aaatCvAUfKttLearuWrP9MDH5MBPbIqV92AaeXatLxBI9gBaebbnrfifHhDYfgasaacH8akY=wiFfYdH8Gipec8Eeeu0xXdbba9frFj0=OqFfea0dXdd9vqai=hGuQ8kuc9pgc9s8qqaq=dirpe0xb9q8qiLsFr0=vr0=vr0dc8meaabaqaciaacaGaaeqabaqabeGadaaakeaacqWGUbGBdaqhaaWcbaacciGae8xSdegabaGaem4AaSgaaaaa@3143@(*S*_*w*_) is the number of times base *α *occurs at position *k *of the sites in *S*_*w*_.

The posterior probability *P*(*c*|*D*) of a configuration is simply proportional to the product of (60) and (61), i.e. *P*(*c*|*D*) ∝ *P*(*D*|*c*)*P*(*c*|{*π*}). To sample from the posterior probability *P*(*c*|*D*) over all possible configurations we need a more extensive set of 'moves' then the one described in the Gibbs sampler above. This can be done in a number of ways [24]. One possibility is to pick a sequence at random, to remove all sites currently located in it, and to sample from all ways of putting a new set of sites in, see [25] for details. The set of moves implemented by the PhyloGibbs algorithm [23] is illustrated in Fig. 5. These moves are:

**1. Resampling a segment: **Pick a sequence *m *at random and a random position *i*_*m *_in it. Check if there is a site overlapping the region from *i*_*m *_+ 1 to *i*_*m *_+ *l *in the current configuration *c*. If so, do nothing, i.e. move from *c *to *c*. If the region is free (or a site occurs precisely at *i*_*m *_+ 1 through *i*_*m *_+ *l*) calculate the probabilities *P*(*c'*|*D*) for all configurations *c' *that are obtained by putting a site for any of the WMs *w *at *i*_*m*_, including putting no site at all or putting a site for a new motif. Finally, sample one of these configurations *c' *with probability proportional to *P*(*c'*|*D*).

**2. Moving a site: **Pick one of the sites occurring in *c *and remove it creating configuration *c*^-^. Find all sequence segments *s *of length *l *in *c*^- ^that are not overlapping any site. Calculate the probability *P*(*c'*|*D*) for all configurations that can be obtained by placing a new site for the same WM at any of the free segments *s*. Sample one of these configurations *c' *in proportion to *P*(*c'*|*D*).

**3. Shifting a site group: **Pick one of the sets of sites *S*_*w *_at random. Check how far the sites in *S*_*w *_can be shifted to the left and right without colliding with other sites in the current configuration *c*. Denote these maximal shifts by *l*_max _and *r*_max_. For every shift *h *between *h *= *l*_*max *_and *h *= *r*_*max *_calculate the probability *P*(*c'*|*D*) of the configuration that would result if all sites in *S*_*w *_were shifted by an amount *h*. Sample one of the configurations *c' *in proportion to probability *P*(*c'*|*D*).

One of the main advantages of the motif sampling approach over EM algorithms is that it is much less likely to get stuck in local optima. In particular, one can sample multiple motifs without becoming trapped in bad local optima. Another advantage is that one can obtain rigorous posterior probabilities for sites appearing at different positions which allows for a more reliable separation of trustworthy predictions from spurious ones (see [23]). As for the single motif, i.e. our discussion below equation (50), we can here also use 'informative' priors for each of the motifs. That is, if we have a set motifs for which known sites are available we can use the base counts in these sites as 'pseudocounts' γαk
 MathType@MTEF@5@5@+=feaafiart1ev1aaatCvAUfKttLearuWrP9MDH5MBPbIqV92AaeXatLxBI9gBaebbnrfifHhDYfgasaacH8akY=wiFfYdH8Gipec8Eeeu0xXdbba9frFj0=OqFfea0dXdd9vqai=hGuQ8kuc9pgc9s8qqaq=dirpe0xb9q8qiLsFr0=vr0=vr0dc8meaabaqaciaacaGaaeqabaqabeGadaaakeaaiiGacqWFZoWzdaqhaaWcbaGae8xSdegabaGaem4AaSgaaaaa@3180@ of priors for corresponding motifs in the binding sites configurations. That is, apart from inferring multiple new motifs, we can use informative priors to discover new sites for known motifs at the same time. This can be especially useful when we are trying to find a new motif in a set of sequences that also contains sites for a number of known motifs. If we were to search this data for a single motif then it is quite likely that the search would return one of the known motifs. By searching for multiple motifs at the same time and using informative priors for each of the known motifs we can make sure that known sites will automatically associate with the known motifs, and that the remaining motifs are indeed new motifs. Finally, under the sampling approach one can use arbitrarily complicated priors *P*(*c*) on configurations, including priors that demand that certain combinations of sites occur at certain specified distances of each other, in particular orientations, etcetera. In the EM approach such complex priors would typically cause the dynamic programming solution to summing over all configurations to break down.

The main disadvantage of the motif sampling approach is of course speed. To obtain accurate statistics one needs to sample for a long time, and the time necessary grows with the product of the size of the data-set *D*, the total number of sites, and the number of motifs. In contrast, the dynamic programming approaches outlined in section "Finding WM matches" allow for efficient computation of sums over all possible configurations even for very large input data, allowing one to search very large sequences for matches to sets of WMs, which is computationally infeasible with motif sampling algorithms.

As mentioned already, motif sampling was introduced more than a decade ago [20]. Since then a significant number of algorithms has been developed including [21,26-28], and probably many more. The PhyloGibbs algorithm [23] introduces several extensions such as simulated annealing to find the configuration with maximal probability, simultaneously sampling multiple motifs, and taking the phylogenetic relationships between the sequences into account (discussed below).

## Clustering sites and motifs

There are several situations in which we may want to cluster sets of binding sites. This demand for instance arises whenever we have obtained a set of sequence segments that are thought to each have regulatory function, without knowing the specific function of any of the segments. For example, several researchers have used so-called 'phylogenetic footprinting', the identification of short overly conserved segments in alignments of orthologous intergenic regions from related genomes, to gather large collections of putative regulatory sites [29-32]. It is reasonable to assume that most of these short segments contain a regulatory site for some regulatory factor, but we do not know which sites are sites for the same factor nor how many different regulatory factors are represented in data.

Formally, given a dataset *D *of sequence segments, we want to partition this dataset into subsets such that all segments within a subset contain a regulatory site for a common regulatory factor, and different subsets correspond to different regulatory factors. In addition, we want to multiply align all the segments within each subset. Thus, for this problem the set of hypotheses is all possible ways in which the set *D *can be partitioned into subsets, and all possible ways in which the sequences in each subset can be multiply aligned. Let us denote possible configurations by *C*. Each configuration *C *consists of a set of subsets *c *∈ *C *that each consist of a collection of sequences from *D*. The union of these subsets *c *of course equals *D*. In addition *C *specifies, for each subset c, an alignment *S*_*c *_of sequence segments that are taken from the sequences in *c*. For simplicity we will assume that all these sequence segments are of fixed length *l *in all subsets. That is, *C *specifies a partition of the sequences in *D *into subsets *c*, and it specifies where in each of the sequences the regulatory site of length *l *occurs, thereby specifying length-*l *alignments *S*_*c *_for each subset *c*. We now want to calculate the probability *P*(*D*|*C*) of the data given a configuration *C*. We can generally separate *P*(*D*|*C*) into a contribution of the sites (those segments from the sequences in hypothesized regulatory sites) and the bases outside these segments that are scored according to a background model.

*P*(*D*|*C*)*= P*(*D*_*sites*_|*C*)*P*(*D*_*bg*_|*C*).

For simplicity we will use a background model that assigns a probability 1/4 to each base (extensions to more complex background models are straight forward). In that case the contribution *P*(*D*_bg_|*C*) is constant, i.e. does not depend on *C *and we just consider *P*(*D*_sites_|*C*). This probability can be written as a product of independent contributions from each subset

P(Dsites|C)=∏c∈CP(Sc),
 MathType@MTEF@5@5@+=feaafiart1ev1aaatCvAUfKttLearuWrP9MDH5MBPbIqV92AaeXatLxBI9gBaebbnrfifHhDYfgasaacH8akY=wiFfYdH8Gipec8Eeeu0xXdbba9frFj0=OqFfea0dXdd9vqai=hGuQ8kuc9pgc9s8qqaq=dirpe0xb9q8qiLsFr0=vr0=vr0dc8meaabaqaciaacaGaaeqabaqabeGadaaakeaacqWGqbaucqGGOaakcqWGebardaWgaaWcbaacbaGae83CamNae8xAaKMae8hDaqNae8xzauMae83CamhabeaakiabcYha8jabdoeadjabcMcaPiabg2da9maarafabaGaemiuaaLaeiikaGIaem4uam1aaSbaaSqaaiabdogaJbqabaGccqGGPaqkaSqaaiabdogaJjabgIGiolabdoeadbqab0Gaey4dIunakiabcYcaSaaa@47C2@

where *S*_*c *_is the alignment of sites in subset *c*. The probability *P*(*S*_*c*_) is just the probability that all sequence segments in *S*_*c *_derive from a common WM. The probability *P*(*S*_*c*_) is simply given by replacing *S*_*i *_with *S*_*c *_in the right-hand side of equation (50).

To obtain the posterior probability

P(C|D)=P(D|C)P(C)∑C′P(D|C′)P(C′)
 MathType@MTEF@5@5@+=feaafiart1ev1aaatCvAUfKttLearuWrP9MDH5MBPbIqV92AaeXatLxBI9gBaebbnrfifHhDYfgasaacH8akY=wiFfYdH8Gipec8Eeeu0xXdbba9frFj0=OqFfea0dXdd9vqai=hGuQ8kuc9pgc9s8qqaq=dirpe0xb9q8qiLsFr0=vr0=vr0dc8meaabaqaciaacaGaaeqabaqabeGadaaakeaacqWGqbaucqGGOaakcqWGdbWqcqGG8baFcqWGebarcqGGPaqkcqGH9aqpdaWcaaqaaiabdcfaqjabcIcaOiabdseaejabcYha8jabdoeadjabcMcaPiabdcfaqjabcIcaOiabdoeadjabcMcaPaqaamaaqababaGaemiuaaLaeiikaGIaemiraqKaeiiFaWNafm4qamKbauaacqGGPaqkcqWGqbaucqGGOaakcuWGdbWqgaqbaiabcMcaPaWcbaGafm4qamKbauaaaeqaniabggHiLdaaaaaa@4C1D@

we also need a prior *P*(*C*) over partitions. The simplest prior is of course to assign a uniform prior *P*(*C*) = constant. Note however that, a uniform prior over partitions may correspond to a very peaked prior with respect to the number of clusters. That is, given a dataset with 100 sequences there are astronomically more partitions of the data into, say, 30 subsets than there are partitions of the data into, say, 2 subsets. If one wants a uniform prior over the number of clusters one needs to assign a probability *P*(*C*) ∝ 1/S|C||D|
 MathType@MTEF@5@5@+=feaafiart1ev1aaatCvAUfKttLearuWrP9MDH5MBPbIqV92AaeXatLxBI9gBaebbnrfifHhDYfgasaacH8akY=wiFfYdH8Gipec8Eeeu0xXdbba9frFj0=OqFfea0dXdd9vqai=hGuQ8kuc9pgc9s8qqaq=dirpe0xb9q8qiLsFr0=vr0=vr0dc8meaabaqaciaacaGaaeqabaqabeGadaaakeaacqWGtbWudaqhaaWcbaGaeiiFaWNaem4qamKaeiiFaWhabaGaeiiFaWNaemiraqKaeiiFaWhaaaaa@3628@, where |*D*| is the total number of sequences in *D*, |*C*| is the number of subsets in *C*, and S|C||D|
 MathType@MTEF@5@5@+=feaafiart1ev1aaatCvAUfKttLearuWrP9MDH5MBPbIqV92AaeXatLxBI9gBaebbnrfifHhDYfgasaacH8akY=wiFfYdH8Gipec8Eeeu0xXdbba9frFj0=OqFfea0dXdd9vqai=hGuQ8kuc9pgc9s8qqaq=dirpe0xb9q8qiLsFr0=vr0=vr0dc8meaabaqaciaacaGaaeqabaqabeGadaaakeaacqWGtbWudaqhaaWcbaGaeiiFaWNaem4qamKaeiiFaWhabaGaeiiFaWNaemiraqKaeiiFaWhaaaaa@3628@ is the number of possible partitions of |*D*| objects into |*C*| subsets, which is called a Stirling number of the second kind [33]. Note that with this prior a particular configuration *C *with, say, 2 subsets will have a much higher a priori probability than a configuration with, say, 30 subsets. That is, it is impossible to be a priori completely ignorant about partitions in general *and *about the number of subsets at the same time. Again there is no easy way to find the configuration *C *with maximal posterior probability. A fast procedure for determining a state *C *with high posterior probability is through hierarchical clustering. One starts out with each sequence in *D *forming a subset on its own. For every pair of sequences *s*, and *s' *one then calculates the probability of the configuration *C*(*s*, *s'*, *i*, *i'*) that is obtained when the subsets *s *and *s' *are joined into a cluster, putting the hypothesized sites at positions *i *and *i' *respectively. We then find the combination (*s*, *s'*, *i*, *i*) with maximal *P*(*C*(*s*, *s'*, *i*, *i'*)|*D*) and create the corresponding state *C*(*s*, *s'*, *i*, *i'*). This procedure is repeated, i.e. at each iteration two subsets are fused so as to maximize *P*(*C*|*D*). The iteration stops when there is no more subset merger that would increase *P*(*C*|*D*). The great disadvantage of this procedure is that it generally leads to highly suboptimal local optima in *P*(*C*|*D*).

A better alternative is to use Markov chain Monte-Carlo sampling and simulated annealing. A simple and effective move-set is as follows

1. Select one of the sequences in *D *at random and remove it from its current subset thereby creating a configuration *C*^-^.

2. For each of the subsets in *C*^- ^consider the configuration *C*(*c*, *i*) when the removed sequence *s *is put into subset *c *and the length-*l *site is *s *is started at position *i*. Also consider the configuration *C*(0) which is obtained by putting the sequence *s *in a subset of its own. Calculate *P*(*C*|*D*) for all these configurations and sample one of the configurations in proportion to these probabilities.

**Figure 6:**
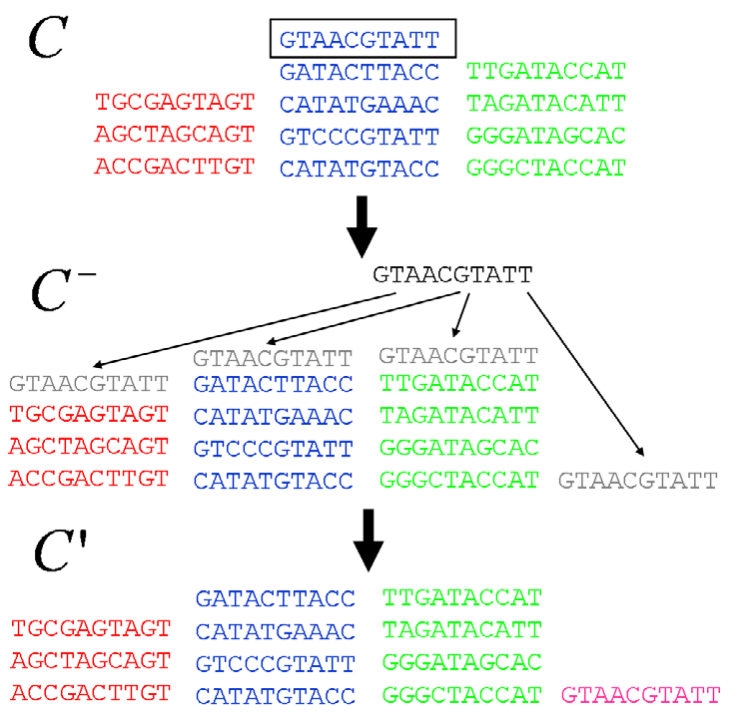
**Illustration of the move-set for binding site clustering.** Starting from a configuration *C *with three clusters, the top sequence in the blue cluster is chosen for resampling. It is removed from its cluster to produce configuration *C*^-^. Probabilities are then calculated for all configurations that would be obtained by inserting the sequence into any of the clusters or a new cluster (gray sequences), and finally one of these (*C'*) is sampled. In this example the sequence was placed in a new cluster. For illustration purposes we have assumed all sequences in *D *have precisely the length *l *of the hypothesized site, so that each sequence can only be aligned in one way with any cluster. In general the sequences in *D *will be longer than *l *and one would also sample over all ways that the sequence can be aligned with each of the clusters.

These steps are illustrated in Fig. 6.

By repeating these two steps one can sample from the posterior distribution *P*(*C*|*D*) over all possible configurations. Through simulated annealing, i.e. sampling from *P*(*C*|*D*)_*^β ^*_and slowly increasing *β*, one can attempt to locate the configuration C* which globally maximizes *P*(*C*|*D*). The PROCSE software [34] implements such a Markov chain Monte-Carlo scheme for simultaneously clustering and aligning sets of sequences that are thought to contain regulatory sites and it has been used to predict regulons in bacteria genome-wide. It has also been used to automatically curate sets of experimentally determined binding sites [35]. PROCSE first determines a 'reference configuration' C* through simulated annealing and then performs another sampling run, i.e. with *β *= 1, to determine the posterior probabilities of the clusters that occur in the reference state C*.

An almost identical procedure as just described can be used to cluster motifs or arbitrary combinations of motifs and sequences. Application of different motif finding algorithms to the same dataset, or application of the same algorithm to related datasets, often results in sets of inferred motifs that show clear commonalities. One is thus often interested in analyzing sets of motifs to identify which motifs are really different, and which motifs might represent a common underlying WM.

As we have seen in section "Finding WM matches" all our information about a motif, i.e. a WM *w*, can be represented by counts nαk
 MathType@MTEF@5@5@+=feaafiart1ev1aaatCvAUfKttLearuWrP9MDH5MBPbIqV92AaeXatLxBI9gBaebbnrfifHhDYfgasaacH8akY=wiFfYdH8Gipec8Eeeu0xXdbba9frFj0=OqFfea0dXdd9vqai=hGuQ8kuc9pgc9s8qqaq=dirpe0xb9q8qiLsFr0=vr0=vr0dc8meaabaqaciaacaGaaeqabaqabeGadaaakeaacqWGUbGBdaqhaaWcbaacciGae8xSdegabaGaem4AaSgaaaaa@3143@ that represent the number of observations of base *α *at position *k *of the sites. So more generally, we will assume that when we are given 'a motif' this information can always be represented by a set of counts nαk
 MathType@MTEF@5@5@+=feaafiart1ev1aaatCvAUfKttLearuWrP9MDH5MBPbIqV92AaeXatLxBI9gBaebbnrfifHhDYfgasaacH8akY=wiFfYdH8Gipec8Eeeu0xXdbba9frFj0=OqFfea0dXdd9vqai=hGuQ8kuc9pgc9s8qqaq=dirpe0xb9q8qiLsFr0=vr0=vr0dc8meaabaqaciaacaGaaeqabaqabeGadaaakeaacqWGUbGBdaqhaaWcbaacciGae8xSdegabaGaem4AaSgaaaaa@3143@. For example, when we are given WM components wαk
 MathType@MTEF@5@5@+=feaafiart1ev1aaatCvAUfKttLearuWrP9MDH5MBPbIqV92AaeXatLxBI9gBaebbnrfifHhDYfgasaacH8akY=wiFfYdH8Gipec8Eeeu0xXdbba9frFj0=OqFfea0dXdd9vqai=hGuQ8kuc9pgc9s8qqaq=dirpe0xb9q8qiLsFr0=vr0=vr0dc8meaabaqaciaacaGaaeqabaqabeGadaaakeaacqWG3bWDdaqhaaWcbaacciGae8xSdegabaGaem4AaSgaaaaa@3155@ then we transform this into a set of counts nαk
 MathType@MTEF@5@5@+=feaafiart1ev1aaatCvAUfKttLearuWrP9MDH5MBPbIqV92AaeXatLxBI9gBaebbnrfifHhDYfgasaacH8akY=wiFfYdH8Gipec8Eeeu0xXdbba9frFj0=OqFfea0dXdd9vqai=hGuQ8kuc9pgc9s8qqaq=dirpe0xb9q8qiLsFr0=vr0=vr0dc8meaabaqaciaacaGaaeqabaqabeGadaaakeaacqWGUbGBdaqhaaWcbaacciGae8xSdegabaGaem4AaSgaaaaa@3143@ by specifying the pseudocounts γαk
 MathType@MTEF@5@5@+=feaafiart1ev1aaatCvAUfKttLearuWrP9MDH5MBPbIqV92AaeXatLxBI9gBaebbnrfifHhDYfgasaacH8akY=wiFfYdH8Gipec8Eeeu0xXdbba9frFj0=OqFfea0dXdd9vqai=hGuQ8kuc9pgc9s8qqaq=dirpe0xb9q8qiLsFr0=vr0=vr0dc8meaabaqaciaacaGaaeqabaqabeGadaaakeaaiiGacqWFZoWzdaqhaaWcbaGae8xSdegabaGaem4AaSgaaaaa@3180@ of a prior, and the effective total number of observations *n *on which the wαk
 MathType@MTEF@5@5@+=feaafiart1ev1aaatCvAUfKttLearuWrP9MDH5MBPbIqV92AaeXatLxBI9gBaebbnrfifHhDYfgasaacH8akY=wiFfYdH8Gipec8Eeeu0xXdbba9frFj0=OqFfea0dXdd9vqai=hGuQ8kuc9pgc9s8qqaq=dirpe0xb9q8qiLsFr0=vr0=vr0dc8meaabaqaciaacaGaaeqabaqabeGadaaakeaacqWG3bWDdaqhaaWcbaacciGae8xSdegabaGaem4AaSgaaaaa@3155@ are based:

nαk=wαk(n+γk)−γαk.
 MathType@MTEF@5@5@+=feaafiart1ev1aaatCvAUfKttLearuWrP9MDH5MBPbIqV92AaeXatLxBI9gBaebbnrfifHhDYfgasaacH8akY=wiFfYdH8Gipec8Eeeu0xXdbba9frFj0=OqFfea0dXdd9vqai=hGuQ8kuc9pgc9s8qqaq=dirpe0xb9q8qiLsFr0=vr0=vr0dc8meaabaqaciaacaGaaeqabaqabeGadaaakeaacqWGUbGBdaqhaaWcbaacciGae8xSdegabaGaem4AaSgaaOGaeyypa0Jaem4DaC3aa0baaSqaaiab=f7aHbqaaiabdUgaRbaakiabcIcaOiabd6gaUjabgUcaRiab=n7aNnaaCaaaleqabaGaem4AaSgaaOGaeiykaKIaeyOeI0Iae83SdC2aa0baaSqaaiab=f7aHbqaaiabdUgaRbaakiabc6caUaaa@44CE@

Without loss of generality we can thus think of these counts nαk
 MathType@MTEF@5@5@+=feaafiart1ev1aaatCvAUfKttLearuWrP9MDH5MBPbIqV92AaeXatLxBI9gBaebbnrfifHhDYfgasaacH8akY=wiFfYdH8Gipec8Eeeu0xXdbba9frFj0=OqFfea0dXdd9vqai=hGuQ8kuc9pgc9s8qqaq=dirpe0xb9q8qiLsFr0=vr0=vr0dc8meaabaqaciaacaGaaeqabaqabeGadaaakeaacqWGUbGBdaqhaaWcbaacciGae8xSdegabaGaem4AaSgaaaaa@3143@ as deriving from an alignment *S *of sites for the motif. That is, we can generally specify our knowledge about a 'motif' by specifying an alignment *S *of sites drawn from the motif WM.

Such alignments *S *can be clustered and aligned with each other completely analogously to the procedure just described for single sequences. That is, one can think of an alignment *S *as a set of individual sequences *s *that have already been clustered and aligned with each other. When multiple such alignments *S *are mutually aligned and clustered into a larger alignment *S*_*c *_then we calculate the probability *P*(*S*_*c*_) that all sequences in *S*_*c *_derive from a common WM exactly like we did before, i.e. equation (49). Thus, the only difference between clustering and aligning single sequences, and clustering and aligning 'motifs' is that motifs are represented by sets of multiply aligned sequences, and that these motif alignments are so to speak 'glued together' in that these sequences will never be repartitioned during the sampling. The PROCSE software also allows for such pre-clustered and pre-aligned sequences to be submitted as input. In this way arbitrary combinations of single sequences and motifs can be aligned and clustered simultaneously.

## Incorporating phylogeny

In all our approaches so far we have assumed that different sequences that contain binding sites can be considered independent samples from a WM *w*. In addition, the motif finding approaches that we discussed all presume that one is given sets of sequences that are likely to contain sites for common regulatory factors. In many cases researchers use independent biological evidence, such as expression data, to collect such sets of sequences that appear 'co-regulated' [27,36]. Apart from expression data, more recently ChIP-on-chip techniques have been used to collect sets of sequences that appear to be bound by a common regulatory factor, see e.g. [37,38].

Another possibility is to collect sets of orthologous intergenic regions from related species. It is often reasonable to assume that many of the regulatory sites occurring in the ancestor of these species have been maintained and are shared by all or most of the descendants. Therefore, orthologous intergenic sequences can generally be expected to contain sites for common regulatory factors. However, in contrast to sites in collections of upstream regions of genes from a single species, these sites cannot be considered *independent *samples from a common WM. That is, the orthologous sites are related evolutionary, and their sequences will therefore generally be more correlated than independent samples from a WM. Therefore, to correctly analyze orthologous intergenic regions we need to take the phylogenetic relationships of the species into account.

### Binding site evolution

Let us consider a single position in a regulatory site whose WM has components *w*_*α *_at that position. We now want to calculate the probabilities *P*_*αβ*_(*w*, *t*) that over an evolutionary time *t *this position in the site evolves from base *β *to base *α*.

There is a long history of such models for the evolution of amino acids, e.g. see [39,40]. For our application to nucleotide evolution a general treatment of this problem was given by the model of Halpern and Bruno [41]. The rate *u*_*αβ *_at which base *β *is substituted by base *α *during evolution is written as the product of an instantaneous rate of mutation *μ*_*αβ *_from *β *to *α*, and the probability *f*_*αβ *_that a mutation from *β *to *α *will be fixed in the population (which depends on selection), i.e.

*u*_*αβ *_= *f*_*αβ*_*μ*_*αβ*_.

Under this general model the probabilities *P*(*α*|*β*, *w*, *t*) are the solution of the differential equations

dPαβ(w,t)dt=∑γ≠α[uαγPγβ(w,t) −uγαPαβ(w,t)].
 MathType@MTEF@5@5@+=feaafiart1ev1aaatCvAUfKttLearuWrP9MDH5MBPbIqV92AaeXatLxBI9gBaebbnrfifHhDYfgasaacH8akY=wiFfYdH8Gipec8Eeeu0xXdbba9frFj0=OqFfea0dXdd9vqai=hGuQ8kuc9pgc9s8qqaq=dirpe0xb9q8qiLsFr0=vr0=vr0dc8meaabaqaciaacaGaaeqabaqabeGadaaakeaadaWcaaqaaiabdsgaKjabdcfaqnaaBaaaleaaiiGacqWFXoqycqWFYoGyaeqaaOGaeiikaGIaem4DaCNaeiilaWIaemiDaqNaeiykaKcabaGaemizaqMaemiDaqhaaiabg2da9maaqafabaGaei4waSLaemyDau3aaSbaaSqaaiab=f7aHjab=n7aNbqabaGccqWGqbaudaWgaaWcbaGae83SdCMae8NSdigabeaakiabcIcaOiabdEha3jabcYcaSiabdsha0jabcMcaPiabbccaGiabgkHiTiabdwha1naaBaaaleaacqWFZoWzcqWFXoqyaeqaaOGaemiuaa1aaSbaaSqaaiab=f7aHjab=j7aIbqabaGccqGGOaakcqWG3bWDcqGGSaalcqWG0baDcqGGPaqkcqGGDbqxaSqaaiab=n7aNjabgcMi5kab=f7aHbqab0GaeyyeIuoakiabc6caUaaa@6628@

Note that in the limit of long time the probabilities *P*_*αβ*_(*w*, *t*) become independent of time, i.e. memory of the start state is lost, and by the definition of the WM components the probabilities *P*_*αβ*_(*w*, *t*) limit to *w*_*α*_, i.e.

lim⁡t→∞Pαβ(w,t)=wα.
 MathType@MTEF@5@5@+=feaafiart1ev1aaatCvAUfKttLearuWrP9MDH5MBPbIqV92AaeXatLxBI9gBaebbnrfifHhDYfgasaacH8akY=wiFfYdH8Gipec8Eeeu0xXdbba9frFj0=OqFfea0dXdd9vqai=hGuQ8kuc9pgc9s8qqaq=dirpe0xb9q8qiLsFr0=vr0=vr0dc8meaabaqaciaacaGaaeqabaqabeGadaaakeaadaWfqaqaaiGbcYgaSjabcMgaPjabc2gaTbWcbaGaemiDaqNaeyOKH4QaeyOhIukabeaakiabdcfaqnaaBaaaleaaiiGacqWFXoqycqWFYoGyaeqaaOGaeiikaGIaem4DaCNaeiilaWIaemiDaqNaeiykaKIaeyypa0Jaem4DaC3aaSbaaSqaaiab=f7aHbqabaGccqGGUaGlaaa@4528@

Assuming that the rates *μ*_*αβ *_are given one can then solve [41] for the substitution rates *u*_*αβ *_that will lead to the limit distribution (69):

uαβ=μαβlog⁡[μβαwαμαβwβ]1−μαβwβμβαwα.
 MathType@MTEF@5@5@+=feaafiart1ev1aaatCvAUfKttLearuWrP9MDH5MBPbIqV92AaeXatLxBI9gBaebbnrfifHhDYfgasaacH8akY=wiFfYdH8Gipec8Eeeu0xXdbba9frFj0=OqFfea0dXdd9vqai=hGuQ8kuc9pgc9s8qqaq=dirpe0xb9q8qiLsFr0=vr0=vr0dc8meaabaqaciaacaGaaeqabaqabeGadaaakeaacqWG1bqDdaWgaaWcbaacciGae8xSdeMae8NSdigabeaakiabg2da9iab=X7aTnaaBaaaleaacqWFXoqycqWFYoGyaeqaaOWaaSaaaeaacyGGSbaBcqGGVbWBcqGGNbWzdaWadaqaamaalaaabaGae8hVd02aaSbaaSqaaiab=j7aIjab=f7aHbqabaGccqWG3bWDdaWgaaWcbaGae8xSdegabeaaaOqaaiab=X7aTnaaBaaaleaacqWFXoqycqWFYoGyaeqaaOGaem4DaC3aaSbaaSqaaiab=j7aIbqabaaaaaGccaGLBbGaayzxaaaabaGaeGymaeJaeyOeI0YaaSaaaeaacqWF8oqBdaWgaaWcbaGae8xSdeMae8NSdigabeaakiabdEha3naaBaaaleaacqWFYoGyaeqaaaGcbaGae8hVd02aaSbaaSqaaiab=j7aIjab=f7aHbqabaGccqWG3bWDdaWgaaWcbaGae8xSdegabeaaaaaaaOGaeiOla4caaa@624F@

To solve equation (68) we note that it can be written as a matrix equation. Define the rate matrix **U **through

Uαβ=uαβ−δαβ∑γuγα.
 MathType@MTEF@5@5@+=feaafiart1ev1aaatCvAUfKttLearuWrP9MDH5MBPbIqV92AaeXatLxBI9gBaebbnrfifHhDYfgasaacH8akY=wiFfYdH8Gipec8Eeeu0xXdbba9frFj0=OqFfea0dXdd9vqai=hGuQ8kuc9pgc9s8qqaq=dirpe0xb9q8qiLsFr0=vr0=vr0dc8meaabaqaciaacaGaaeqabaqabeGadaaakeaacqWGvbqvdaWgaaWcbaacciGae8xSdeMae8NSdigabeaakiabg2da9iabdwha1naaBaaaleaacqWFXoqycqWFYoGyaeqaaOGaeyOeI0Iae8hTdq2aaSbaaSqaaiab=f7aHjab=j7aIbqabaGcdaaeqbqaaiabdwha1naaBaaaleaacqWFZoWzcqWFXoqyaeqaaaqaaiab=n7aNbqab0GaeyyeIuoakiabc6caUaaa@46B8@

In terms of this matrix **U **equation (68) becomes

dP(t)dt=U⋅P(t),
 MathType@MTEF@5@5@+=feaafiart1ev1aaatCvAUfKttLearuWrP9MDH5MBPbIqV92AaeXatLxBI9gBaebbnrfifHhDYfgasaacH8akY=wiFfYdH8Gipec8Eeeu0xXdbba9frFj0=OqFfea0dXdd9vqai=hGuQ8kuc9pgc9s8qqaq=dirpe0xb9q8qiLsFr0=vr0=vr0dc8meaabaqaciaacaGaaeqabaqabeGadaaakeaadaWcaaqaaiabdsgaKHqabiab=bfaqjabcIcaOiabdsha0jabcMcaPaqaaiabdsgaKjabdsha0baacqGH9aqpcqWFvbqvcqGHflY1cqWFqbaucqGGOaakcqWG0baDcqGGPaqkcqGGSaalaaa@3EC8@

with matrix **P**(*t*) having components *P*_*αβ*_(*t*). Using the boundary condition *P*_*αβ*_(0) = *δ*_*αβ *_the solution is given by

*P*_*αβ *_(*t*) = (*e*^**U***t*^)_*αβ *_.

In this general model one thus solves for *P*_*αβ*_(*w*, *t*) by first determining **U **using equation (70), and determining its eigenvalues and eigenvectors. However, note that in general the solution is a complicated function of the WM components *w*_*α *_which is not easily amenable to further analysis.

To allow more analytic flexibility we have developed a simpler model of the evolution of binding sites [23,42] that assumes that all mutations are introduced at the same rate, i.e. *μ*_*αβ *_= *μ*, and that the probability of fixation *f*_*αβ *_depends only on the target base *α* i.e. *f*_*αβ *_= *w*_*α*_. Under these assumption the differential equations become

dPαβ(w,t)dt=μ∑γ≠α[wαPγβ(w,t)−wγPαβ(w,t)]=μwα−μPαβ(w,t)
 MathType@MTEF@5@5@+=feaafiart1ev1aaatCvAUfKttLearuWrP9MDH5MBPbIqV92AaeXatLxBI9gBaebbnrfifHhDYfgasaacH8akY=wiFfYdH8Gipec8Eeeu0xXdbba9frFj0=OqFfea0dXdd9vqai=hGuQ8kuc9pgc9s8qqaq=dirpe0xb9q8qiLsFr0=vr0=vr0dc8meaabaqaciaacaGaaeqabaqabeGadaaakeaadaWcaaqaaiabdsgaKjabdcfaqnaaBaaaleaaiiGacqWFXoqycqWFYoGyaeqaaOGaeiikaGIaem4DaCNaeiilaWIaemiDaqNaeiykaKcabaGaemizaqMaemiDaqhaaiabg2da9iab=X7aTnaaqafabaGaei4waSLaem4DaC3aaSbaaSqaaiab=f7aHbqabaGccqWGqbaudaWgaaWcbaGae83SdCMae8NSdigabeaakiabcIcaOiabdEha3jabcYcaSiabdsha0jabcMcaPiabgkHiTiabdEha3naaBaaaleaacqWFZoWzaeqaaOGaemiuaa1aaSbaaSqaaiab=f7aHjab=j7aIbqabaGccqGGOaakcqWG3bWDcqGGSaalcqWG0baDcqGGPaqkcqGGDbqxcqGH9aqpcqWF8oqBcqWG3bWDdaWgaaWcbaGae8xSdegabeaakiabgkHiTiab=X7aTjabdcfaqnaaBaaaleaacqWFXoqycqWFYoGyaeqaaOGaeiikaGIaem4DaCNaeiilaWIaemiDaqNaeiykaKcaleaacqWFZoWzcqGHGjsUcqWFXoqyaeqaniabggHiLdaaaa@759B@

This equation can be easily solved to give

*P*_*αβ*_(*w*, *t*) = *δ*_*αβ*_*e*^-*μt *^+ *w*_*α*_(1- *e*^-*μt*^).

Note that *e*^-*μt *^is the probability that no mutations have taken place during time *t*. We call this no-mutation-probability the *proximity q *= *e*^-*μt *^between the ancestor and the descendant [23]. In terms of the proximity the solution becomes

*P*_*αβ*_(*w*, *q*) = *δ*_*αβ*_*q *+ (1 - *q*)*w*_*α*_.

This expression has a nice simple interpretation. With probability *q *no mutations have taken place in going from *β *to *α *and the bases are identical. With probability (1 - *q*) one or more mutations took place and the probability that one then ends up with base *α *is simply the WM component *w*_*α*_.

### Probability of an orthologous set of bases

Assume that we have a set of orthologous intergenic regions and assume that we know the phylogenetic tree *T *that relates the species from which the regions derive. Consider now a set of orthologous bases *S *from these intergenic regions. That is, the bases in *S *have evolved from a common ancestor base in the common ancestor of the species according to the tree *T*. We now calculate the probability *P*(*S*|*T, w*) that, when evolving from a common ancestor under one of the evolutionary models just discussed, and according to the given phylogenetic tree *T*, the set of bases *S *will result at the leafs of the tree.

Note that the set *S *only specifies the bases at the leafs of the tree *T*, i.e. the bases at the internal nodes are unknown. If we also knew all the bases at the internal nodes we could calculate *P*(*S*|*T*, *w*) simply by multiplying the probabilities *P*_*αβ*_(*w*, *t*) for each branch, i.e.

**Figure 7:**
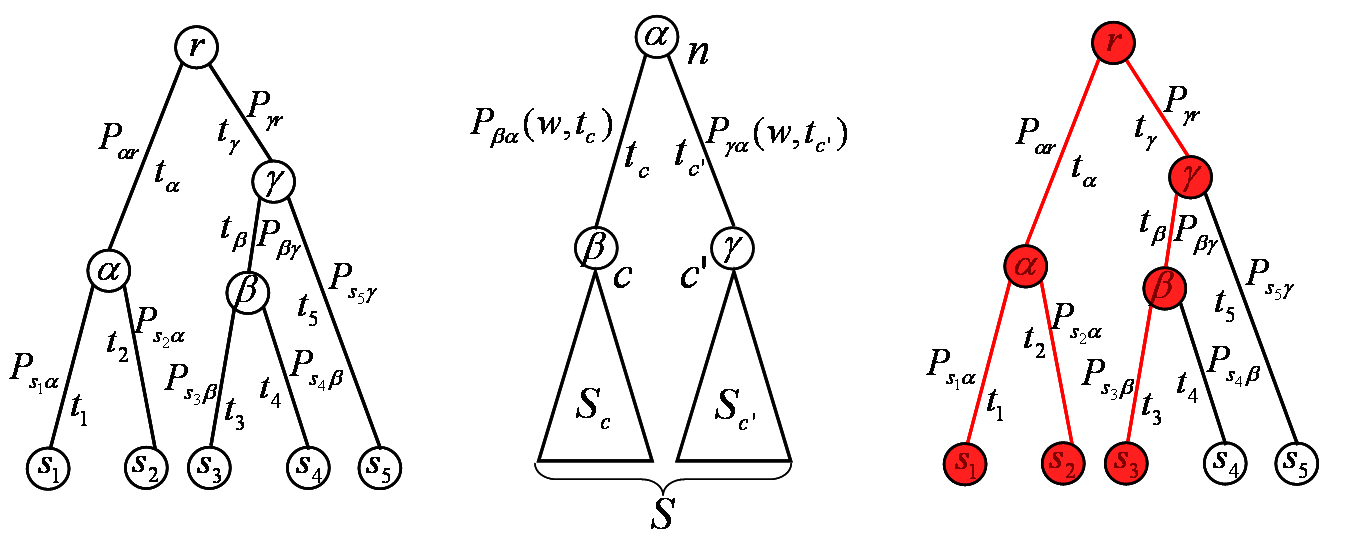
**The evolution of a set of orthologous bases along a phylogenetic tree.** In the left panel the expression (77) is illustrated. For notational simplicity we write *P*_*αβ *_for *P*_*αβ*_(*w*, *t*). The middle panel illustrates the recurion relations (78) with *c *and *c' *the children of node *n*, *S*_*c *_the set of bases in *S *that descend from *c *and *S*_*c' *_the set of bases in *S *that descend from *c'*. The right panel shows expression (77) for a more complex selection pattern with branches evolving according to the WM in red, and those evolving to the background in black.

P(S|T,w)=∏nPsnsa(n)(w,tn),
 MathType@MTEF@5@5@+=feaafiart1ev1aaatCvAUfKttLearuWrP9MDH5MBPbIqV92AaeXatLxBI9gBaebbnrfifHhDYfgasaacH8akY=wiFfYdH8Gipec8Eeeu0xXdbba9frFj0=OqFfea0dXdd9vqai=hGuQ8kuc9pgc9s8qqaq=dirpe0xb9q8qiLsFr0=vr0=vr0dc8meaabaqaciaacaGaaeqabaqabeGadaaakeaacqWGqbaucqGGOaakcqWGtbWucqGG8baFcqWGubavcqGGSaalcqWG3bWDcqGGPaqkcqGH9aqpdaqeqbqaaiabdcfaqnaaBaaaleaacqWGZbWCdaWgaaadbaGaemOBa4gabeaaliabdohaZnaaBaaameaacqWGHbqycqGGOaakcqWGUbGBcqGGPaqkaeqaaaWcbeaakiabcIcaOiabdEha3jabcYcaSiabdsha0naaBaaaleaacqWGUbGBaeqaaOGaeiykaKcaleaacqWGUbGBaeqaniabg+GivdGccqGGSaalaaa@4CAE@

where the product is over all nodes *n*, *s*_*n *_is the base at node *n*, *a*(*n*) is the ancestor of node *n*, and *t*_*n *_is the length of the branch from *a*(*n*) to *n*. This is illustrated in the left panel of Fig. 7.

However, as we do not know the identities of the bases at the internal nodes, we thus have to sum over all possibilities. This can be done using a dynamic programming scheme first presented by Felsenstein [43]. We denote by *D*_*α*_(*n*, *w*) the probability to observe all bases of *S *that are descendants of node *n *of the tree given that node *n *has base *α*. For nodes *n *that are leafs, i.e. bases of *S*, we of course have *D*_*α*_(*n*, *w*) = *δ*_*αsn*_. We can determine *D*_*α*_(*n*, *w*) for all nodes using the following recursion relation

Dα(n,w)=∏m∈c(n)[∑βPαβ(w,tm)Dβ(m,w)].
 MathType@MTEF@5@5@+=feaafiart1ev1aaatCvAUfKttLearuWrP9MDH5MBPbIqV92AaeXatLxBI9gBaebbnrfifHhDYfgasaacH8akY=wiFfYdH8Gipec8Eeeu0xXdbba9frFj0=OqFfea0dXdd9vqai=hGuQ8kuc9pgc9s8qqaq=dirpe0xb9q8qiLsFr0=vr0=vr0dc8meaabaqaciaacaGaaeqabaqabeGadaaakeaacqWGebardaWgaaWcbaacciGae8xSdegabeaakiabcIcaOiabd6gaUjabcYcaSiabdEha3jabcMcaPiabg2da9maarafabaWaamWaaeaadaaeqbqaaiabdcfaqnaaBaaaleaacqWFXoqycqWFYoGyaeqaaOGaeiikaGIaem4DaCNaeiilaWIaemiDaq3aaSbaaSqaaiabd2gaTbqabaGccqGGPaqkcqWGebardaWgaaWcbaGae8NSdigabeaakiabcIcaOiabd2gaTjabcYcaSiabdEha3jabcMcaPaWcbaGae8NSdigabeqdcqGHris5aaGccaGLBbGaayzxaaaaleaacqWGTbqBcqGHiiIZcqWGJbWycqGGOaakcqWGUbGBcqGGPaqkaeqaniabg+GivdGccqGGUaGlaaa@5A0C@

where *c*(*n*) is the set of children of node *n*, and *t*_*m *_is the length of the branch connecting *m *to its parent *n*. This basic recursion is illustrated in the middle panel of Fig. 7. Starting from the leafs we can use (78) to calculate *D*_*α*_(*n*, *w*) for all nodes up to the root of the tree. Finally the probability *P*(*S*|*T, w*) for the whole tree is obtained by summing over the bases of the root node *r*, noting that the prior probability that root *r *has base *α *is *w*_*α*_. This gives

P(S|T,w)=∑αwαDα(r,w).
 MathType@MTEF@5@5@+=feaafiart1ev1aaatCvAUfKttLearuWrP9MDH5MBPbIqV92AaeXatLxBI9gBaebbnrfifHhDYfgasaacH8akY=wiFfYdH8Gipec8Eeeu0xXdbba9frFj0=OqFfea0dXdd9vqai=hGuQ8kuc9pgc9s8qqaq=dirpe0xb9q8qiLsFr0=vr0=vr0dc8meaabaqaciaacaGaaeqabaqabeGadaaakeaacqWGqbaucqGGOaakcqWGtbWucqGG8baFcqWGubavcqGGSaalcqWG3bWDcqGGPaqkcqGH9aqpdaaeqbqaaiabdEha3naaBaaaleaaiiGacqWFXoqyaeqaaOGaemiraq0aaSbaaSqaaiab=f7aHbqabaGccqGGOaakcqWGYbGCcqGGSaalcqWG3bWDcqGGPaqkaSqaaiab=f7aHbqab0GaeyyeIuoakiabc6caUaaa@4719@

In complete analogy we can calculate the probability *P*(*S*|*T*, *b*) of the column of bases *S *assuming that they evolved under a background model *b*. which is given by background probabilities *b*_*α*_. To obtain *P*(*S*|*T, b*) we just replace *P*_*αβ*_(*w*, *t*) with *P*_*αβ*_(*b, t*) for each branch of the tree in equation (78) and replace *w*_*α *_with *b*_*α *_in (79). Finally, we can also easily accommodate cases in which the regulatory site has been maintained in some but not all species. That is, we can have some branches of the tree *T *evolve according to the background model *b *whereas other branches evolve according to the WM column *w*, simply by using *P*_*αβ*_(*w*, *t*) for each branch evolving according to the WM, and using *P*_*αβ*_(*b*, *t*) for each branch evolving according to the background. An example of such a more complicated 'selection pattern' is shown in the right panel of Fig. 7.

### Finding sites and modules in multiple alignments

To apply the probabilities *P*(*S*|*T*, *w*) and *P*(*S*|*T*, *b*) to a set of orthologous intergenic regions we of course first have to identify which sets of bases in these sequences form orthologous groups. That is, we have to produce a multiple alignment of the orthologous intergenic regions. Given a multiple alignment we can then assume that every column of the alignment corresponds to a set of orthologous bases. The problem of producing accurate multiple alignments of non-coding sequences is extremely challenging and is beyond the scope of this article. There are now a number of algorithms available that focus specifically on alignment of non-coding DNA [44-46], although our personal experience is that consistency based methods [47,48] and evolutionary explicit progressive alignment [49] often outperform these methods significantly. From this point on we will assume that a global multiple alignment of the orthologous intergenic regions is given and that we can assume that vertically aligned bases in this alignment are orthologous.

We can use the probabilities *P*(*S*|*T*, *w*) and *P*(*S*|*T*, *b*) that we derived above to extend the formalism of sections "Finding WM matches" and "Finding clusters of binding sites: regulatory modules" to multiple alignments. The simplest way of doing this is to take one of the sequences in the multiple alignment as a *reference *sequence and to consider all binding site configurations for this reference sequence. This is often natural since in many cases we are really only interested in finding regulatory sites in one particular species and it is thus natural to take this species as a reference.

Let *s*_[*i*,*l*] _denote a segment of length *l *in this reference sequence, and let *S*_[*i*,*l*] _denote the corresponding block in the multiple alignment. To calculate the probability that a regulatory site occurs at *s*_[*i*,*l*] _we will now calculate the probabilities of observing the alignment segment *S*_[*i*,*l*] _under different assumptions for the selection that was operating at each branch of the tree *T *relating the species in the alignment. The simplest assumptions about the selection are that either all sequences in *S*_[*i*,*l*] _evolved according to the background model, i.e. using expression *P*(*S*|*T*, *b*) for each column *S *in *S*_[*i*,*l*]_, or that all sequences evolved according to WM *w*, i.e. using *P*(*S*|*T*, *w*) for each column *S *in *S*_[*i*,*l*]_. Many algorithms [23,50,51] in fact restrict themselves to these two possibilities. However, there are many other possibilities. If there are *B *branches in the tree then there are in principle 2^*B *^possible ways of assigning selection to the branches, i.e. either WM *w *or background *b *for each branch. Formally, to calculate the probability that a regulatory site occurs at *s*_[*i*,*l*] _we would want to consider all 2^*B *- 1 ^'selection patterns' *σ *for which *s*_[*i*,*l*] _is under selection of the WM *w*. We would want to assign prior probabilities *P*(*σ*) to all 2^*B *^possible selection patterns *σ*, and calculate the probabilities *P*(*S*_[*i*,*l*]_|*T*, *σ*) for each. Finally, by summing *P*(*S*_[*i*,*l*]_|*T*,*σ*)*P*(*σ*) over all selection patterns for which *s*_[*i*,*l*] _is under selection of the WM *w *one would obtain the total probability of the data *S*_[*i*,*l*] _under the assumption that a regulatory site occurs at *s*[*i*,*l*]. Unfortunately, there is no simple way of determining a reasonable distribution *P*(*σ*) and the sum would generally involve a large number of terms. This author is not aware of any algorithm that currently implements this general scheme.

**Figure 8:**
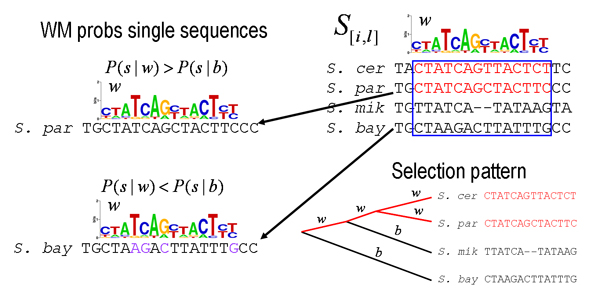
**Probability for an alignment block assuming a site occurs in the reference sequence.** In the top right an alignment segment S_[*i*,*l*] _is shown for the species *S. cerevisiae *(the reference), *S. paradoxus*, *S. mikatae*, and *S. bayanus*. First we check which sequences are gaplessly aligned with the reference. In this case *S. mikatae *contains a gap and the background model is assigned to this sequence. The reference has the WM model assigned by default (indicated in red). In the left the probabilities of the sequences from *S. paradoxus *and *S. bayanus *are compared with the WM (shown as a logo). It turns out the *S. paradoxus *sequence scores better for the WM than for background but the *S. bayanus *sequence scores better to background than to the WM, because of some mismatches to the WM consensus (bases in purple). Finally, on the bottom right the phylogenetic tree is indicated with the branches that evolve according to the WM in red, and those evolving according to the background in black.

In the MotEvo algorithm [35] a single selection pattern σ_* _is chosen that best fits the alignment and the sequences in it. Note first that, since WMs have a fixed width, a site in the reference species can only occur in another species if the corresponding segment in that species is gaplessly aligned with the site in the reference species. Therefore, we first check which of the other sequences in *S*_[*i*,*l*] _are gaplessly aligned with the reference sequence and which are not. For those sequences not gaplessly aligned with the reference we assign the background evolution model to the branches leading to these sequences. For each of the other sequences *s *in *S*_[*i*,*l*] _we calculate the probability *P*(*s*|*w*) of the sequence under the WM *w*, and the probability *P*(*s*|*b*) of the sequence under the background model *b*. Whenever *P*(*s*|*w*) > *P*(*s*|*b*) we assign the WM model to the branch leading to *s*, and for all others we assign the background model. Finally, we assume that an internal node evolved according to the WM if any of its descendants do. This defines a unique selection pattern *σ *for *S*_[*i*,*l*] _and we calculate *P*(*S*|*T*, *w*) using this selection pattern. The procedure is illustrated in Fig. 8.

We also calculate *P*(*S*_[*i*,*l*]_|*T*, *b*) assuming all branches evolved according to background. Finally, if we assign a prior probability *π *that a site occurs at *s*_[*i*,*l*]_ the posterior probability *P*(site|*S*_[*i*,*l*]_) that the reference species has a functional site at *i *becomes

P(site|S[i,l])=P(S[i,l]|T,w)πP(S[i,l]|T,w)π+P(S[i,l]|T,b)(1−π).
 MathType@MTEF@5@5@+=feaafiart1ev1aaatCvAUfKttLearuWrP9MDH5MBPbIqV92AaeXatLxBI9gBaebbnrfifHhDYfgasaacH8akY=wiFfYdH8Gipec8Eeeu0xXdbba9frFj0=OqFfea0dXdd9vqai=hGuQ8kuc9pgc9s8qqaq=dirpe0xb9q8qiLsFr0=vr0=vr0dc8meaabaqaciaacaGaaeqabaqabeGadaaakeaacqWGqbaucqGGOaakieaacqWFZbWCcqWFPbqAcqWF0baDcqWFLbqzcqGG8baFcqWGtbWudaWgaaWcbaGaei4waSLaemyAaKMaeiilaWIaemiBaWMaeiyxa0fabeaakiabcMcaPiabg2da9maalaaabaGaemiuaaLaeiikaGIaem4uam1aaSbaaSqaaiabcUfaBjabdMgaPjabcYcaSiabdYgaSjabc2faDbqabaGccqGG8baFcqWGubavcqGGSaalcqWG3bWDcqGGPaqkiiGacqGFapaCaeaacqWGqbaucqGGOaakcqWGtbWudaWgaaWcbaGaei4waSLaemyAaKMaeiilaWIaemiBaWMaeiyxa0fabeaakiabcYha8jabdsfaujabcYcaSiabdEha3jabcMcaPiab+b8aWjabgUcaRiabdcfaqjabcIcaOiabdofatnaaBaaaleaacqGGBbWwcqWGPbqAcqGGSaalcqWGSbaBcqGGDbqxaeqaaOGaeiiFaWNaemivaqLaeiilaWIaemOyaiMaeiykaKIaeiikaGIaeGymaeJaeyOeI0Iae4hWdaNaeiykaKcaaiabc6caUaaa@77AD@

This is essentially the expression used by the MotEvo algorithm [35] to find regulatory sites. The MONKEY algorithm finds regulatory sites in a very similar manner. Instead of the simple evolutionary model (76) MONKEY uses the more general Halpern/Bruno model (70). However, MONKEY does not consider the possibility that the site is conserved in some but not all of the aligned species, i.e. it assumes that either all branches of the tree evolve according to the WM, or all branches evolve according to background.

Instead of looking at one sequence segment at a time, we can of course also use this formalism to calculate sums of the probabilities of all possible binding site configurations as in section "Finding WM matches". Instead of calculating the probability *P*(*s*_[*i*,*l*]_|*w*) of a single sequence segment under the WM we instead calculate the probability *P*(*S*_[*i*,*l*]_|*w*) of the ungapped alignment block at that location using the procedure just outlined. That is, for every segment *s*_[*i*,*l*] _we find which other sequences are ungapped at the segment and choose which of these are evolving according to the WM based on the probabilities of the individual sequence segments under the WM. The generalization of equation (20) is then simply

Fn=Fn−1P(S[n−1,1]|T,b)πbg+∑wFn−lwP(S[n−lw,lw]|T,w)πw.
 MathType@MTEF@5@5@+=feaafiart1ev1aaatCvAUfKttLearuWrP9MDH5MBPbIqV92AaeXatLxBI9gBaebbnrfifHhDYfgasaacH8akY=wiFfYdH8Gipec8Eeeu0xXdbba9frFj0=OqFfea0dXdd9vqai=hGuQ8kuc9pgc9s8qqaq=dirpe0xb9q8qiLsFr0=vr0=vr0dc8meaabaqaciaacaGaaeqabaqabeGadaaakeaacqWGgbGrdaWgaaWcbaGaemOBa4gabeaakiabg2da9iabdAeagnaaBaaaleaacqWGUbGBcqGHsislcqaIXaqmaeqaaOGaemiuaaLaeiikaGIaem4uam1aaSbaaSqaaiabcUfaBjabd6gaUjabgkHiTiabigdaXiabcYcaSiabigdaXiabc2faDbqabaGccqGG8baFcqWGubavcqGGSaalcqWGIbGycqGGPaqkiiGacqWFapaCdaWgaaWcbaacbaGae4NyaiMae43zaCgabeaakiabgUcaRmaaqafabaGaemOray0aaSbaaSqaaiabd6gaUjabgkHiTiabdYgaSnaaBaaameaacqWG3bWDaeqaaaWcbeaakiabdcfaqjabcIcaOiabdofatnaaBaaaleaacqGGBbWwcqWGUbGBcqGHsislcqWGSbaBdaWgaaadbaGaem4DaChabeaaliabcYcaSiabdYgaSnaaBaaameaacqWG3bWDaeqaaSGaeiyxa0fabeaakiabcYha8jabdsfaujabcYcaSiabdEha3jabcMcaPiab=b8aWnaaBaaaleaacqWG3bWDaeqaaaqaaiabdEha3bqab0GaeyyeIuoakiabc6caUaaa@6EBD@

Note that position *n *here always refers to the *n*th base in the reference sequence.

Finally, using this formalism we can of course also search for regulatory modules in multiple alignments in complete analogy with the equations in section "Finding clusters of binding sites: regulatory modules".

This procedure has been implemented for two-species alignments in the Stubb algorithm [42]. Applying the Stubb algorithm to predict developmental regulatory modules in *Drosophila *it was shown in [52] that using two-species alignments improves predictions of the locations of regulatory modules over the single species algorithms.

### Motif finding incorporating phylogeny

In section "Motif finding" we discussed two approaches to motif finding, one based on maximizing the probability *P*(*D*|*w*) of the data given a WM *w *using expectation maximization, and one using Markov chain Monte-Carlo sampling to find the site configuration *c *that maximizes the posterior *P*(*c*|*D*). These methods can also be extended in a straightforward way to multiple alignments and we now discuss these in turn.

#### Motif EM incorporating phylogeny

The PhyME algorithm implements an extension of the MEME algorithm to multiple alignments of orthologous intergenic sequences from related species. It uses a reference species and considers all configurations of binding sites that can be assigned to the reference species in the same way as discussed in the previous section, i.e. it uses equation (81) to calculate the overall likelihood *P*(*D*|*w*) of the alignment given the WM *w*. The evolutionary model that is used by PhyME to score ungapped alignment blocks *P*(*S*_[*i*,*l*]_|*w*) is precisely the simplified model of equation (76). However, like MONKEY and in contrast to MotEvo, PhyME assumes that either all branches in the tree evolved according to the WM model, or that all evolved according to background.

To maximize *P*(*D*|*w*) with respect to the WM PhyMe needs to solve, for each column *k *in the WM, the equations

dP(D|w)dwαk=constant ∀α.
 MathType@MTEF@5@5@+=feaafiart1ev1aaatCvAUfKttLearuWrP9MDH5MBPbIqV92AaeXatLxBI9gBaebbnrfifHhDYfgasaacH8akY=wiFfYdH8Gipec8Eeeu0xXdbba9frFj0=OqFfea0dXdd9vqai=hGuQ8kuc9pgc9s8qqaq=dirpe0xb9q8qiLsFr0=vr0=vr0dc8meaabaqaciaacaGaaeqabaqabeGadaaakeaadaWcaaqaaiabdsgaKjabdcfaqjabcIcaOiabdseaejabcYha8jabdEha3jabcMcaPaqaaiabdsgaKjabdEha3naaDaaaleaaiiGacqWFXoqyaeaacqWGRbWAaaaaaOGaeyypa0dcbaGae43yamMae43Ba8Mae4NBa4Mae43CamNae4hDaqNae4xyaeMae4NBa4Mae4hDaqNaeeiiaaIaeyiaIiIae8xSdeMae8Nla4caaa@4B09@

Note that for the single sequence case, the derivative of *P*(*s*|*w*) with respect to the WM components wαk
 MathType@MTEF@5@5@+=feaafiart1ev1aaatCvAUfKttLearuWrP9MDH5MBPbIqV92AaeXatLxBI9gBaebbnrfifHhDYfgasaacH8akY=wiFfYdH8Gipec8Eeeu0xXdbba9frFj0=OqFfea0dXdd9vqai=hGuQ8kuc9pgc9s8qqaq=dirpe0xb9q8qiLsFr0=vr0=vr0dc8meaabaqaciaacaGaaeqabaqabeGadaaakeaacqWG3bWDdaqhaaWcbaacciGae8xSdegabaGaem4AaSgaaaaa@3155@, was very simple, i.e. see (43). In contrast,the derivative *dP*(*S*_[*i*,*l*]_*T*, *w*)/*d*wαk
 MathType@MTEF@5@5@+=feaafiart1ev1aaatCvAUfKttLearuWrP9MDH5MBPbIqV92AaeXatLxBI9gBaebbnrfifHhDYfgasaacH8akY=wiFfYdH8Gipec8Eeeu0xXdbba9frFj0=OqFfea0dXdd9vqai=hGuQ8kuc9pgc9s8qqaq=dirpe0xb9q8qiLsFr0=vr0=vr0dc8meaabaqaciaacaGaaeqabaqabeGadaaakeaacqWG3bWDdaqhaaWcbaacciGae8xSdegabaGaem4AaSgaaaaa@3155@ is a much more complicated function of the WM components wαk
 MathType@MTEF@5@5@+=feaafiart1ev1aaatCvAUfKttLearuWrP9MDH5MBPbIqV92AaeXatLxBI9gBaebbnrfifHhDYfgasaacH8akY=wiFfYdH8Gipec8Eeeu0xXdbba9frFj0=OqFfea0dXdd9vqai=hGuQ8kuc9pgc9s8qqaq=dirpe0xb9q8qiLsFr0=vr0=vr0dc8meaabaqaciaacaGaaeqabaqabeGadaaakeaacqWG3bWDdaqhaaWcbaacciGae8xSdegabaGaem4AaSgaaaaa@3155@ which needs to be calculated recursively just as *P*(*S*_[*i*,*l*]_|*T*, *w*) itself. Here it becomes particularly advantageous that in the simplified model (76) the probability *P*_*αβ*_(*w*,*t*) is such a simple function of the WM components. We do not discuss the mathematical details of solving (82) here except for mentioning the fact that it involves an iterative procedure similar to EM that leads to a local optimum in *P*(*D*|*w*).

#### Motif sampling incorporating phylogeny

We now discuss extending the motif sampling approach of section "Motif sampling" to alignments of phylogenetically related sequences. Remember that in the motif sampling approach, instead of summing over all possible binding site configurations to calculate the probability *P*(*D*|*w*) conditioned on the WM, we condition on a particular binding site configuration *c *and calculate the probability *P*(*D*|*c*) by integrating over all possible WMs *w*.

**Figure 9:**
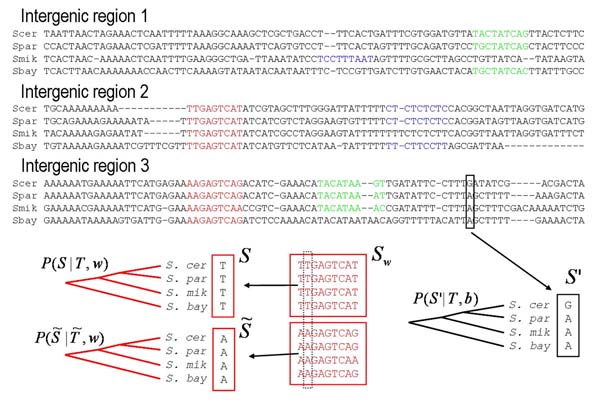
**An input data-set consisting of the multiple alignments of 3 sets of orthologous intergenic regions from *S. cerevisiae, S. paradoxus, S. mikatae*, and *S. bayanus*.** A binding site configuration *c *with sites for three motifs (red, green, and blue) is indicated. Note that each site is extended over all sequences that are locally gaplessly aligned. Most columns in the data are scored according to the background model in this configuration. On the lower right one example of an aligment column *S' *that is scored according to the background is shown. On the lower left the alignment *S*_*w *_of sequences assigned to the red motif *w *is shown. A single column from this alignment consists of two independent columns, *S *and S˜
 MathType@MTEF@5@5@+=feaafiart1ev1aaatCvAUfKttLearuWrP9MDH5MBPbIqV92AaeXatLxBI9gBaebbnrfifHhDYfgasaacH8akY=wiFfYdH8Gipec8Eeeu0xXdbba9frFj0=OqFfea0dXdd9vqai=hGuQ8kuc9pgc9s8qqaq=dirpe0xb9q8qiLsFr0=vr0=vr0dc8meaabaqaciaacaGaaeqabaqabeGadaaakeaacuWGtbWugaacaaaa@2DEA@, that derive from the multiple alignments of intergenic regions 2 and 3 respectively. The trees on the left show that under this configuration, the columns *S *and S˜
 MathType@MTEF@5@5@+=feaafiart1ev1aaatCvAUfKttLearuWrP9MDH5MBPbIqV92AaeXatLxBI9gBaebbnrfifHhDYfgasaacH8akY=wiFfYdH8Gipec8Eeeu0xXdbba9frFj0=OqFfea0dXdd9vqai=hGuQ8kuc9pgc9s8qqaq=dirpe0xb9q8qiLsFr0=vr0=vr0dc8meaabaqaciaacaGaaeqabaqabeGadaaakeaacuWGtbWugaacaaaa@2DEA@ are both assumed to have evolved according to the same WM *w*, as indicated by the red branches on their phylogenetic trees *T *and T˜
 MathType@MTEF@5@5@+=feaafiart1ev1aaatCvAUfKttLearuWrP9MDH5MBPbIqV92AaeXatLxBI9gBaebbnrfifHhDYfgasaacH8akY=wiFfYdH8Gipec8Eeeu0xXdbba9frFj0=OqFfea0dXdd9vqai=hGuQ8kuc9pgc9s8qqaq=dirpe0xb9q8qiLsFr0=vr0=vr0dc8meaabaqaciaacaGaaeqabaqabeGadaaakeaacuWGubavgaacaaaa@2DEC@.

Instead of a set of single sequences the input will now generally consist of a set of multiple alignments of orthologous non-coding sequences or a combination of multiple alignments and single sequences. As in section "Motif sampling" we want to consider all possible configurations *c *of binding sites that can be assigned to the input data *D*, and calculate the probability of the data *P*(*D*|*c*) for each possible configuration. Whereas for single sequences the space of all possible configurations existed simply of all ways in which sets of non-overlapping windows can be assigned to the sequences, i.e. see Fig. 2, for multiple alignments the situation is a bit more complicated and illustrated in Fig. 9.

Above we assumed that a reference sequence *s *is given for each multiple alignment and that the set of binding ste configurations for the alignment is simply the set of all binding site configurations for the reference species. In the PhyloGibbs algorithm [23] there is no reference sequence and each sequence in the multiple alignment is treated the same. A site can be hypothesized to occur at any position of any of the sequences. By definition the algorithm assumes that, whenever a site occurs in one species, it will also occur in all other species that are gaplessly aligned with it at that location. That is, sites are automatically extended to all species that are mutually gaplessly aligned at that position, see Fig. 9. The algorithm makes sure to only allow configurations in which none of the sites overlap.

Next we need to calculate *P*(*D*|*c*) for every possible such configuration *c*. This probability *P*(*D*|*c*) is given by an equation essentially identical to equation (61). However, instead of single background bases *σ *with probability *b*_*σ *_we will now have alignment columns *S *with probability *P*(*S*|*T*, *b*) as calculated in section "Probability of an orthologous set of bases". The set of sequences *S*_*w *_assigned to a WM *w *will now generally consist of several ungapped segments from the multiple alignments, i.e. alignment blocks, and possibly some single sequences as well, see Fig. 9. The probability *P*(*S*_*w*_) will again be an integral over all possible WMs but the integrand in this case will be considerably more complicated. For simplicity let's focus on a single column from the set *S*_*w *_of sequence segments and alignment blocks. For simplicity assume that this column from *S*_*w *_contains two independent columns *S*, and S˜
 MathType@MTEF@5@5@+=feaafiart1ev1aaatCvAUfKttLearuWrP9MDH5MBPbIqV92AaeXatLxBI9gBaebbnrfifHhDYfgasaacH8akY=wiFfYdH8Gipec8Eeeu0xXdbba9frFj0=OqFfea0dXdd9vqai=hGuQ8kuc9pgc9s8qqaq=dirpe0xb9q8qiLsFr0=vr0=vr0dc8meaabaqaciaacaGaaeqabaqabeGadaaakeaacuWGtbWugaacaaaa@2DEA@ from the multiple alignments, see Fig. 9. The probability *P*(*S*_*w*_) would then be formally given by

P(Sw)=∫P(S|T,w)P(S˜|T˜,w)P(w)dw,
 MathType@MTEF@5@5@+=feaafiart1ev1aaatCvAUfKttLearuWrP9MDH5MBPbIqV92AaeXatLxBI9gBaebbnrfifHhDYfgasaacH8akY=wiFfYdH8Gipec8Eeeu0xXdbba9frFj0=OqFfea0dXdd9vqai=hGuQ8kuc9pgc9s8qqaq=dirpe0xb9q8qiLsFr0=vr0=vr0dc8meaabaqaciaacaGaaeqabaqabeGadaaakeaacqWGqbaucqGGOaakcqWGtbWudaWgaaWcbaGaem4DaChabeaakiabcMcaPiabg2da9maapeaabaGaemiuaaLaeiikaGIaem4uamLaeiiFaWNaemivaqLaeiilaWIaem4DaCNaeiykaKcaleqabeqdcqGHRiI8aOGaemiuaaLaeiikaGIafm4uamLbaGaacqGG8baFcuWGubavgaacaiabcYcaSiabdEha3jabcMcaPiabdcfaqjabcIcaOiabdEha3jabcMcaPiabdsgaKjabdEha3jabcYcaSaaa@4FAA@

where *T *is the phylogenetic tree of alignment column *S*, T˜
 MathType@MTEF@5@5@+=feaafiart1ev1aaatCvAUfKttLearuWrP9MDH5MBPbIqV92AaeXatLxBI9gBaebbnrfifHhDYfgasaacH8akY=wiFfYdH8Gipec8Eeeu0xXdbba9frFj0=OqFfea0dXdd9vqai=hGuQ8kuc9pgc9s8qqaq=dirpe0xb9q8qiLsFr0=vr0=vr0dc8meaabaqaciaacaGaaeqabaqabeGadaaakeaacuWGubavgaacaaaa@2DEC@ the phylogenetic tree of alignment column S˜
 MathType@MTEF@5@5@+=feaafiart1ev1aaatCvAUfKttLearuWrP9MDH5MBPbIqV92AaeXatLxBI9gBaebbnrfifHhDYfgasaacH8akY=wiFfYdH8Gipec8Eeeu0xXdbba9frFj0=OqFfea0dXdd9vqai=hGuQ8kuc9pgc9s8qqaq=dirpe0xb9q8qiLsFr0=vr0=vr0dc8meaabaqaciaacaGaaeqabaqabeGadaaakeaacuWGtbWugaacaaaa@2DEA@, and the expressions *P*(*S*|*T*,*w*) and *P*(S˜
 MathType@MTEF@5@5@+=feaafiart1ev1aaatCvAUfKttLearuWrP9MDH5MBPbIqV92AaeXatLxBI9gBaebbnrfifHhDYfgasaacH8akY=wiFfYdH8Gipec8Eeeu0xXdbba9frFj0=OqFfea0dXdd9vqai=hGuQ8kuc9pgc9s8qqaq=dirpe0xb9q8qiLsFr0=vr0=vr0dc8meaabaqaciaacaGaaeqabaqabeGadaaakeaacuWGtbWugaacaaaa@2DEA@|T˜
 MathType@MTEF@5@5@+=feaafiart1ev1aaatCvAUfKttLearuWrP9MDH5MBPbIqV92AaeXatLxBI9gBaebbnrfifHhDYfgasaacH8akY=wiFfYdH8Gipec8Eeeu0xXdbba9frFj0=OqFfea0dXdd9vqai=hGuQ8kuc9pgc9s8qqaq=dirpe0xb9q8qiLsFr0=vr0=vr0dc8meaabaqaciaacaGaaeqabaqabeGadaaakeaacuWGubavgaacaaaa@2DEC@,*w*)  are given as in equations (78) and (79). To calculate the integral notice that, formally, the expression *P*(*S*|*T*,*w*) is a polynomial in the WM components of the following form

P(S|T,w)=∑kck∏α(wα)mαk
 MathType@MTEF@5@5@+=feaafiart1ev1aaatCvAUfKttLearuWrP9MDH5MBPbIqV92AaeXatLxBI9gBaebbnrfifHhDYfgasaacH8akY=wiFfYdH8Gipec8Eeeu0xXdbba9frFj0=OqFfea0dXdd9vqai=hGuQ8kuc9pgc9s8qqaq=dirpe0xb9q8qiLsFr0=vr0=vr0dc8meaabaqaciaacaGaaeqabaqabeGadaaakeaacqWGqbaucqGGOaakcqWGtbWucqGG8baFcqWGubavcqGGSaalcqWG3bWDcqGGPaqkcqGH9aqpdaaeqbqaaiabdogaJnaaBaaaleaacqWGRbWAaeqaaOWaaebuaeaacqGGOaakcqWG3bWDdaWgaaWcbaacciGae8xSdegabeaakiabcMcaPmaaCaaaleqabaGaemyBa02aa0baaWqaaiab=f7aHbqaaiabdUgaRbaaaaaaleaacqWFXoqyaeqaniabg+GivdaaleaacqWGRbWAaeqaniabggHiLdaaaa@4A92@

where the prefactors *c*_*k *_depend on the branch lengths in the tree and the mαk
 MathType@MTEF@5@5@+=feaafiart1ev1aaatCvAUfKttLearuWrP9MDH5MBPbIqV92AaeXatLxBI9gBaebbnrfifHhDYfgasaacH8akY=wiFfYdH8Gipec8Eeeu0xXdbba9frFj0=OqFfea0dXdd9vqai=hGuQ8kuc9pgc9s8qqaq=dirpe0xb9q8qiLsFr0=vr0=vr0dc8meaabaqaciaacaGaaeqabaqabeGadaaakeaacqWGTbqBdaqhaaWcbaacciGae8xSdegabaGaem4AaSgaaaaa@3141@ are sets of integers. The expression *P*(S˜
 MathType@MTEF@5@5@+=feaafiart1ev1aaatCvAUfKttLearuWrP9MDH5MBPbIqV92AaeXatLxBI9gBaebbnrfifHhDYfgasaacH8akY=wiFfYdH8Gipec8Eeeu0xXdbba9frFj0=OqFfea0dXdd9vqai=hGuQ8kuc9pgc9s8qqaq=dirpe0xb9q8qiLsFr0=vr0=vr0dc8meaabaqaciaacaGaaeqabaqabeGadaaakeaacuWGtbWugaacaaaa@2DEA@|T˜
 MathType@MTEF@5@5@+=feaafiart1ev1aaatCvAUfKttLearuWrP9MDH5MBPbIqV92AaeXatLxBI9gBaebbnrfifHhDYfgasaacH8akY=wiFfYdH8Gipec8Eeeu0xXdbba9frFj0=OqFfea0dXdd9vqai=hGuQ8kuc9pgc9s8qqaq=dirpe0xb9q8qiLsFr0=vr0=vr0dc8meaabaqaciaacaGaaeqabaqabeGadaaakeaacuWGubavgaacaaaa@2DEC@,*w*) can of course also be written in this form. Denote its prefactors c˜k˜
 MathType@MTEF@5@5@+=feaafiart1ev1aaatCvAUfKttLearuWrP9MDH5MBPbIqV92AaeXatLxBI9gBaebbnrfifHhDYfgasaacH8akY=wiFfYdH8Gipec8Eeeu0xXdbba9frFj0=OqFfea0dXdd9vqai=hGuQ8kuc9pgc9s8qqaq=dirpe0xb9q8qiLsFr0=vr0=vr0dc8meaabaqaciaacaGaaeqabaqabeGadaaakeaacuWGJbWygaacamaaBaaaleaacuWGRbWAgaacaaqabaaaaa@2FA4@, and its exponents m˜αk
 MathType@MTEF@5@5@+=feaafiart1ev1aaatCvAUfKttLearuWrP9MDH5MBPbIqV92AaeXatLxBI9gBaebbnrfifHhDYfgasaacH8akY=wiFfYdH8Gipec8Eeeu0xXdbba9frFj0=OqFfea0dXdd9vqai=hGuQ8kuc9pgc9s8qqaq=dirpe0xb9q8qiLsFr0=vr0=vr0dc8meaabaqaciaacaGaaeqabaqabeGadaaakeaacuWGTbqBgaacamaaDaaaleaaiiGacqWFXoqyaeaacqWGRbWAaaaaaa@3150@. Using this the integral can be rewritten as

P(Sw)=∑k,k˜ckc˜k˜∫∏α(wα)mαk+m˜αk˜+γα−1dw.
 MathType@MTEF@5@5@+=feaafiart1ev1aaatCvAUfKttLearuWrP9MDH5MBPbIqV92AaeXatLxBI9gBaebbnrfifHhDYfgasaacH8akY=wiFfYdH8Gipec8Eeeu0xXdbba9frFj0=OqFfea0dXdd9vqai=hGuQ8kuc9pgc9s8qqaq=dirpe0xb9q8qiLsFr0=vr0=vr0dc8meaabaqaciaacaGaaeqabaqabeGadaaakeaacqWGqbaucqGGOaakcqWGtbWudaWgaaWcbaGaem4DaChabeaakiabcMcaPiabg2da9maaqafabaGaem4yam2aaSbaaSqaaiabdUgaRbqabaGccuWGJbWygaacamaaBaaaleaacuWGRbWAgaacaaqabaaabaGaem4AaSMaeiilaWIafm4AaSMbaGaaaeqaniabggHiLdGcdaWdbaqaamaarafabaGaeiikaGIaem4DaC3aaSbaaSqaaGGaciab=f7aHbqabaGccqGGPaqkaSqaaiab=f7aHbqab0Gaey4dIunakmaaCaaaleqabaGaemyBa02aa0baaWqaaiab=f7aHbqaaiabdUgaRbaaliabgUcaRiqbd2gaTzaaiaWaa0baaWqaaiab=f7aHbqaaiqbdUgaRzaaiaaaaSGaey4kaSIae83SdC2aaSbaaWqaaiab=f7aHbqabaWccqGHsislcqaIXaqmaaaabeqab0Gaey4kIipakiabdsgaKjabdEha3jabc6caUaaa@5DFF@

Note that each monomial term of the form ∏α(wα)mαk+m˜αk˜+γα−1
MathType@MTEF@5@5@+=feaafiart1ev1aaatCvAUfKttLearuWrP9MDH5MBPbIqV92AaeXatLxBI9gBaebbnrfifHhDYfgasaacH8akY=wiFfYdH8Gipec8Eeeu0xXdbba9frFj0=OqFfea0dXdd9vqai=hGuQ8kuc9pgc9s8qqaq=dirpe0xb9q8qiLsFr0=vr0=vr0dc8meaabaqaciaacaGaaeqabaqabeGadaaakeaadaqeqaqaaiabcIcaOiabdEha3naaBaaaleaaiiGacqWFXoqyaeqaaOGaeiykaKYaaWbaaSqabeaacqWGTbqBdaqhaaadbaGae8xSdegabaGaem4AaSgaaSGaey4kaSIafmyBa0MbaGaadaqhaaadbaGae8xSdegabaGafm4AaSMbaGaaaaWccqGHRaWkcqWFZoWzdaWgaaadbaGae8xSdegabeaaliabgkHiTiabigdaXaaaaeaacqWFXoqyaeqaniabg+Givdaaaa@459C@ can be easily integrated using the general expression (31). We then obtain for the integral

P(Sw)=∑k,k˜ckc˜k˜Γ(γ)Γ(mk+m˜k˜+γ)∏αΓ(mαk+m˜αk˜+γα)Γ(γα).
 MathType@MTEF@5@5@+=feaafiart1ev1aaatCvAUfKttLearuWrP9MDH5MBPbIqV92AaeXatLxBI9gBaebbnrfifHhDYfgasaacH8akY=wiFfYdH8Gipec8Eeeu0xXdbba9frFj0=OqFfea0dXdd9vqai=hGuQ8kuc9pgc9s8qqaq=dirpe0xb9q8qiLsFr0=vr0=vr0dc8meaabaqaciaacaGaaeqabaqabeGadaaakeaacqWGqbaucqGGOaakcqWGtbWudaWgaaWcbaGaem4DaChabeaakiabcMcaPiabg2da9maaqafabaGaem4yam2aaSbaaSqaaiabdUgaRbqabaGccuWGJbWygaacamaaBaaaleaacuWGRbWAgaacaaqabaGcdaWcaaqaaiabfo5ahjabcIcaOGGaciab=n7aNjabcMcaPaqaaiabfo5ahjabcIcaOiabd2gaTnaaCaaaleqabaGaem4AaSgaaOGaey4kaSIafmyBa0MbaGaadaahaaWcbeqaaiqbdUgaRzaaiaaaaOGaey4kaSIae83SdCMaeiykaKcaamaarafabaWaaSaaaeaacqqHtoWrcqGGOaakcqWGTbqBdaqhaaWcbaGae8xSdegabaGaem4AaSgaaOGaey4kaSIafmyBa0MbaGaadaqhaaWcbaGae8xSdegabaGafm4AaSMbaGaaaaGccqGHRaWkcqWFZoWzdaWgaaWcbaGae8xSdegabeaakiabcMcaPaqaaiabfo5ahjabcIcaOiab=n7aNnaaBaaaleaacqWFXoqyaeqaaOGaeiykaKcaaaWcbaGae8xSdegabeqdcqGHpis1aaWcbaGaem4AaSMaeiilaWIafm4AaSMbaGaaaeqaniabggHiLdGccqGGUaGlaaa@6D51@

So in principle we can analytically determine the value of the integral *P*(*S*_*w*_) in this way. However, the number of terms in the above sum grows exponentially both with the number of sequences in each alignment and, more importantly, with the number of alignments under the integral. That is, if the configuration *c *contains 10 multiple alignment segments for WM *w*, then even if there were only 10 terms for each alignment column *P*(*S*|*T*, *w*), there would still be 10^10 ^terms in total. In practice we thus have to resort to approximations of the above integral. The approach that is taken in the PhyloGibbs algorithm is to approximate the expression *P*(*S*|*T*, *w*) with a monomial for each alignment column, i.e.

P(S|T,w)≈c∏α(wα)xα,
 MathType@MTEF@5@5@+=feaafiart1ev1aaatCvAUfKttLearuWrP9MDH5MBPbIqV92AaeXatLxBI9gBaebbnrfifHhDYfgasaacH8akY=wiFfYdH8Gipec8Eeeu0xXdbba9frFj0=OqFfea0dXdd9vqai=hGuQ8kuc9pgc9s8qqaq=dirpe0xb9q8qiLsFr0=vr0=vr0dc8meaabaqaciaacaGaaeqabaqabeGadaaakeaacqWGqbaucqGGOaakcqWGtbWucqGG8baFcqWGubavcqGGSaalcqWG3bWDcqGGPaqkcqGHijYUcqWGJbWydaqeqbqaaiabcIcaOiabdEha3naaBaaaleaaiiGacqWFXoqyaeqaaOGaeiykaKYaaWbaaSqabeaacqWG4baEdaWgaaadbaGae8xSdegabeaaaaaaleaacqWFXoqyaeqaniabg+GivdGccqGGSaalaaa@45C6@

where the *x*_*α *_may be non-integer. The prefactor *c *and the exponents *x*_*α *_are set such that the first moments of the approximation match those of *P*(*S*|*T*, *w*). That is, we demand that

c∫∏α(wα)xαdw=∫P(S|T,w)dw.
 MathType@MTEF@5@5@+=feaafiart1ev1aaatCvAUfKttLearuWrP9MDH5MBPbIqV92AaeXatLxBI9gBaebbnrfifHhDYfgasaacH8akY=wiFfYdH8Gipec8Eeeu0xXdbba9frFj0=OqFfea0dXdd9vqai=hGuQ8kuc9pgc9s8qqaq=dirpe0xb9q8qiLsFr0=vr0=vr0dc8meaabaqaciaacaGaaeqabaqabeGadaaakeaacqWGJbWydaWdbaqaamaarafabaGaeiikaGIaem4DaC3aaSbaaSqaaGGaciab=f7aHbqabaGccqGGPaqkdaahaaWcbeqaaiabdIha4naaBaaameaacqWFXoqyaeqaaaaaaSqaaiab=f7aHbqab0Gaey4dIunakiabdsgaKjabdEha3jabg2da9maapeaabaGaemiuaaLaeiikaGIaem4uamLaeiiFaWNaemivaqLaeiilaWIaem4DaCNaeiykaKIaemizaqMaem4DaChaleqabeqdcqGHRiI8aaWcbeqab0Gaey4kIipakiabc6caUaaa@4EAF@

and

c∫wβ∏α(wα)xαdw=∫wβP(S|T,w)dw
 MathType@MTEF@5@5@+=feaafiart1ev1aaatCvAUfKttLearuWrP9MDH5MBPbIqV92AaeXatLxBI9gBaebbnrfifHhDYfgasaacH8akY=wiFfYdH8Gipec8Eeeu0xXdbba9frFj0=OqFfea0dXdd9vqai=hGuQ8kuc9pgc9s8qqaq=dirpe0xb9q8qiLsFr0=vr0=vr0dc8meaabaqaciaacaGaaeqabaqabeGadaaakeaacqWGJbWydaWdbaqaaiabdEha3naaBaaaleaaiiGacqWFYoGyaeqaaOWaaebuaeaacqGGOaakcqWG3bWDdaWgaaWcbaGae8xSdegabeaakiabcMcaPmaaCaaaleqabaGaemiEaG3aaSbaaWqaaiab=f7aHbqabaaaaaWcbaGae8xSdegabeqdcqGHpis1aOGaemizaqMaem4DaCNaeyypa0Zaa8qaaeaacqWG3bWDdaWgaaWcbaGae8NSdigabeaakiabdcfaqjabcIcaOiabdofatjabcYha8jabdsfaujabcYcaSiabdEha3jabcMcaPiabdsgaKjabdEha3bWcbeqab0Gaey4kIipaaSqabeqaniabgUIiYdaaaa@5453@

for all *β*. As shown in [23] this fixes *c *and the relative sizes of the *x*_*α *_but leaves ∑_*α *_*x*_*α *_still free. The absolute magnitude of the *x*_*α *_we set so as to approximate the second moments, i.e. such that

c∫wβwγ∏α(wα)xαdw≈∫wβwγP(S|T,w)dw.
 MathType@MTEF@5@5@+=feaafiart1ev1aaatCvAUfKttLearuWrP9MDH5MBPbIqV92AaeXatLxBI9gBaebbnrfifHhDYfgasaacH8akY=wiFfYdH8Gipec8Eeeu0xXdbba9frFj0=OqFfea0dXdd9vqai=hGuQ8kuc9pgc9s8qqaq=dirpe0xb9q8qiLsFr0=vr0=vr0dc8meaabaqaciaacaGaaeqabaqabeGadaaakeaacqWGJbWydaWdbaqaaiabdEha3naaBaaaleaaiiGacqWFYoGyaeqaaOGaem4DaC3aaSbaaSqaaiab=n7aNbqabaGcdaqeqbqaaiabcIcaOiabdEha3naaBaaaleaacqWFXoqyaeqaaOGaeiykaKYaaWbaaSqabeaacqWG4baEdaWgaaadbaGae8xSdegabeaaaaaaleaacqWFXoqyaeqaniabg+GivdGccqWGKbazcqWG3bWDcqGHijYUdaWdbaqaaiabdEha3naaBaaaleaacqWFYoGyaeqaaOGaem4DaC3aaSbaaSqaaiab=n7aNbqabaGccqWGqbaucqGGOaakcqWGtbWucqGG8baFcqWGubavcqGGSaalcqWG3bWDcqGGPaqkcqWGKbazcqWG3bWDaSqabeqaniabgUIiYdaaleqabeqdcqGHRiI8aOGaeiOla4caaa@5C8A@

for all combinations of *β *and *γ*. With these approximations the integral for *P*(*S*_*w*_) becomes simply

P(Sw)=cc˜Γ(γ)Γ(x+x˜+γ)∏αΓ(xα+x˜α+γα)Γ(γα),
 MathType@MTEF@5@5@+=feaafiart1ev1aaatCvAUfKttLearuWrP9MDH5MBPbIqV92AaeXatLxBI9gBaebbnrfifHhDYfgasaacH8akY=wiFfYdH8Gipec8Eeeu0xXdbba9frFj0=OqFfea0dXdd9vqai=hGuQ8kuc9pgc9s8qqaq=dirpe0xb9q8qiLsFr0=vr0=vr0dc8meaabaqaciaacaGaaeqabaqabeGadaaakeaacqWGqbaucqGGOaakcqWGtbWudaWgaaWcbaGaem4DaChabeaakiabcMcaPiabg2da9iabdogaJjqbdogaJzaaiaWaaSaaaeaacqqHtoWrcqGGOaakiiGacqWFZoWzcqGGPaqkaeaacqqHtoWrcqGGOaakcqWG4baEcqGHRaWkcuWG4baEgaacaiabgUcaRiab=n7aNjabcMcaPaaadaqeqbqaamaalaaabaGaeu4KdCKaeiikaGIaemiEaG3aaSbaaSqaaiab=f7aHbqabaGccqGHRaWkcuWG4baEgaacamaaBaaaleaacqWFXoqyaeqaaOGaey4kaSIae83SdC2aaSbaaSqaaiab=f7aHbqabaGccqGGPaqkaeaacqqHtoWrcqGGOaakcqWFZoWzdaWgaaWcbaGae8xSdegabeaakiabcMcaPaaaaSqaaiab=f7aHbqab0Gaey4dIunakiabcYcaSaaa@5E92@

where *x *= ∑_*α *_*x*_*α *_and the variables with a tilde are those of the approximation to *P*(S˜
 MathType@MTEF@5@5@+=feaafiart1ev1aaatCvAUfKttLearuWrP9MDH5MBPbIqV92AaeXatLxBI9gBaebbnrfifHhDYfgasaacH8akY=wiFfYdH8Gipec8Eeeu0xXdbba9frFj0=OqFfea0dXdd9vqai=hGuQ8kuc9pgc9s8qqaq=dirpe0xb9q8qiLsFr0=vr0=vr0dc8meaabaqaciaacaGaaeqabaqabeGadaaakeaacuWGtbWugaacaaaa@2DEA@|T˜
 MathType@MTEF@5@5@+=feaafiart1ev1aaatCvAUfKttLearuWrP9MDH5MBPbIqV92AaeXatLxBI9gBaebbnrfifHhDYfgasaacH8akY=wiFfYdH8Gipec8Eeeu0xXdbba9frFj0=OqFfea0dXdd9vqai=hGuQ8kuc9pgc9s8qqaq=dirpe0xb9q8qiLsFr0=vr0=vr0dc8meaabaqaciaacaGaaeqabaqabeGadaaakeaacuWGubavgaacaaaa@2DEC@, *w*). The crucial point of this approximation procedure is that, at the start of the algorithm, we can determine these approximations, i.e. the values of the *x*_*α*_, for every multiple alignment column *S *that occurs in the input data once and store the results. We thus replace the complex expression *P*(*S*|*T*, *w*) with the simple expression c∏α(wα)xα
MathType@MTEF@5@5@+=feaafiart1ev1aaatCvAUfKttLearuWrP9MDH5MBPbIqV92AaeXatLxBI9gBaebbnrfifHhDYfgasaacH8akY=wiFfYdH8Gipec8Eeeu0xXdbba9frFj0=OqFfea0dXdd9vqai=hGuQ8kuc9pgc9s8qqaq=dirpe0xb9q8qiLsFr0=vr0=vr0dc8meaabaqaciaacaGaaeqabaqabeGadaaakeaacqWGJbWydaqeqaqaaiabcIcaOiabdEha3naaBaaaleaaiiGacqWFXoqyaeqaaOGaeiykaKYaaWbaaSqabeaacqWG4baEdaWgaaadbaGae8xSdegabeaaaaaaleaacqWFXoqyaeqaniabg+Givdaaaa@39D9@ for each alignment column *S*. After that, when we are sampling different configurations, the expression *P*(*S*_*w*_) can be as efficiently calculated as for single sequences. That is, we can simply use equation (62), where nαk
 MathType@MTEF@5@5@+=feaafiart1ev1aaatCvAUfKttLearuWrP9MDH5MBPbIqV92AaeXatLxBI9gBaebbnrfifHhDYfgasaacH8akY=wiFfYdH8Gipec8Eeeu0xXdbba9frFj0=OqFfea0dXdd9vqai=hGuQ8kuc9pgc9s8qqaq=dirpe0xb9q8qiLsFr0=vr0=vr0dc8meaabaqaciaacaGaaeqabaqabeGadaaakeaacqWGUbGBdaqhaaWcbaacciGae8xSdegabaGaem4AaSgaaaaa@3143@(*S*_*w*_) is now the sum over the *x*_*α *_of all the alignment segments that occur in *S*_*w*_.

For the prior over configurations *P*(*c*) PhyloGibbs uses the same priors (60) as for configurations over single sequences. PhyloGibbs uses Markov chain Monte-Carlo sampling to sample the space of all binding site configurations. The move-set employed when sampling binding site configurations in multiple alignments is essentially the same as the move-set for binding site configurations in single sequences illustrated in Fig. 5. The only difference is that 'sites' now typically extend over multiple aligned sequences, as illustrated in Fig. 9. Simulated annealing is used to find a configuration c_* _that maximizes the posterior probability *P*(*c*|*D*). Finally, a further sampling run is used to calculate the posterior probabilities of the sites in configuration c_*_. PhyloGibbs reports both the configuration c_* _and the inferred WMs of the motifs in c_*_, as well as posterior probabilities for all sites occurring in c_*_. In [23] we demonstrate the performance of PhyloGibbs on synthetic data, on individual multiple alignments of orthologous intergenic regions from yeast, and on sets of multiple alignments of intergenic regions from yeast that are bound by a common regulatory factor [38]. These tests show that taking phylogeny into account significantly improves the performance in motif finding.

Finally, it is important to distinguish the motif finding methods that rigorously incorporate phylogeny by probabilistically modeling the evolution of binding sites, such as the PhyME and PhyloGibbs algorithms just discussed, from more *ad hoc *algorithms that use comparative genomic information in various ways in motif finding. This includes for example methods that simply identify significantly conserved sequence segments in multiple alignments, [30-32]. These conserved segments can then be post-processed to search for over-represented motifs. In other approaches, e.g. [29,53], orthologous upstream regions are searched in the same way as set of upstream regions of co-regulated genes from a single species would be searched, i.e. ignoring the evolutionary relationships between the sequences. In other algorithms [54,55] one only takes the topology of the phylogenetic tree into account and searches for length-*l *segments that occur in all orthologous sequences, such that the minimal number of mutations necessary to relate the length-*l *segments, i.e. the parsimony score, is under some prespecified cut-off. Another approach is to first search for significantly conserved segments in orthologous intergenic regions, and to then multiply align conserved segments from the upstream regions of co-regulated genes. This approach is taken by the PhyloCon algorithm [56] which, in spite of its name, ignores the phylogenetic relations between the species.

The biggest challenge for incorporating comparative genomic information in motif finding that is currently outstanding is the treatment of the multiple alignment. It is clear that errors in the multiple alignment can have very deleterious effects on the performance of algorithms such as PhyME, PhyloGibbs, and MotEvo. Ideally one would simultaneously search the space of all multiple alignments and all binding site configurations. However, this space is very large and it is currently unclear if and how it can be effectively searched, especially for large data-sets.
